# Supplementary material for: Water-mediated deracemization of a bisporphyrin helicate assisted by diastereoselective encapsulation of chiral guests
Source: Nat Commun. 2019 Mar 29;10:1457. doi: 10.1038/s41467-019-09443-z (PMC6441078; doi:10.1038/s41467-019-09443-z)
Supplement: Supplementary file 1 — Supplementary Information [file 41467_2019_9443_MOESM1_ESM.pdf]

Supplementary Information

Water-mediated deracemization of a bisporphyrin helicate assisted by diastereoselective encapsulation of chiral guests

Yashima *et al.*

Supplementary Figures

Kinetics and Thermodynamics of Water-Mediated Racemization of (*M*)-$\mathbf{1}_{\mathrm{TBA2}}$ and Its Inclusion Complex with G1.

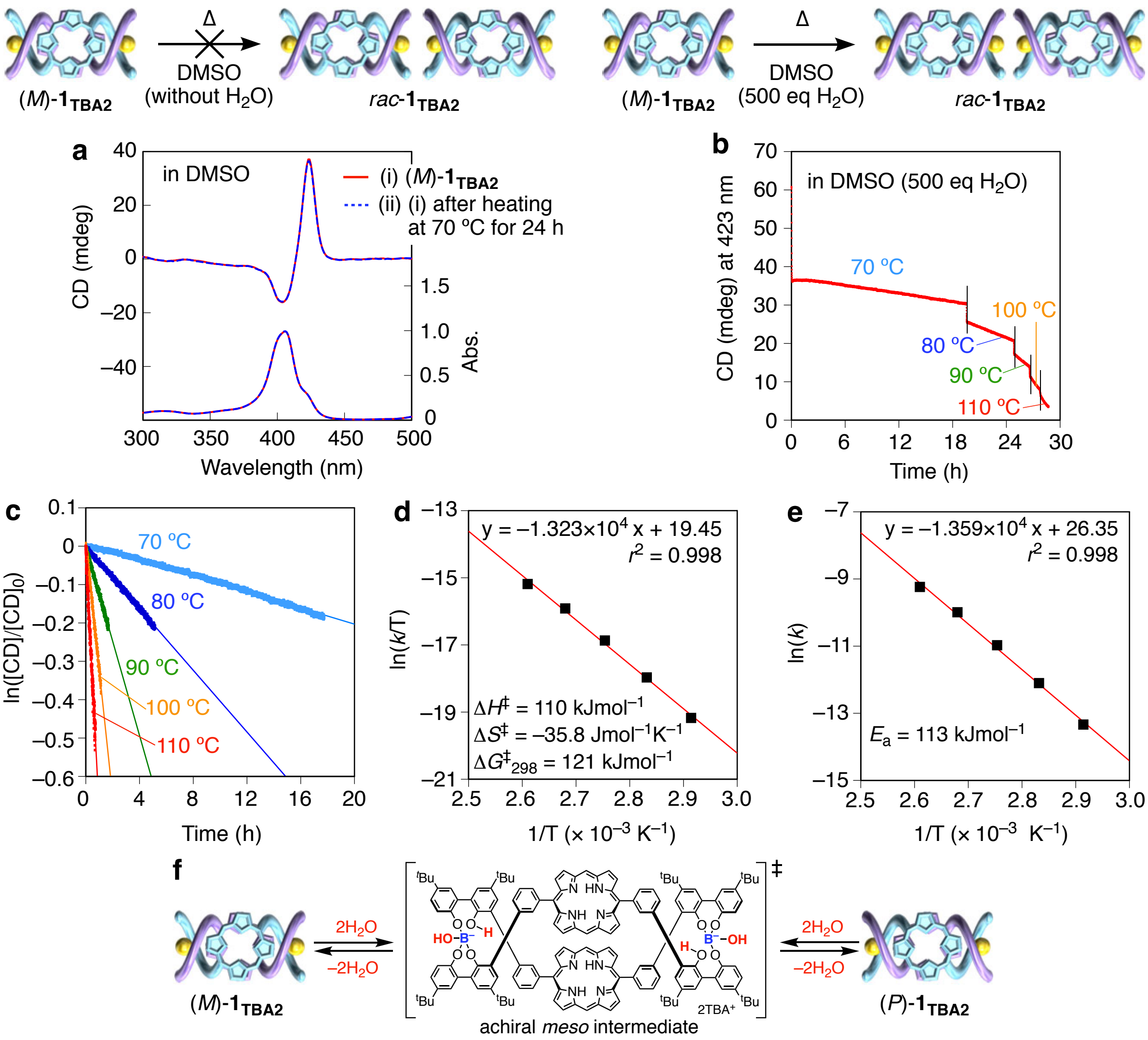

Supplementary Figure 1 | Kinetics and thermodynamics of water-mediated racemization of (*M*)-$\mathbf{1}_{\mathrm{TBA2}}$. (**a**) CD and absorption spectra (anhydrous DMSO, 2.0 μM, 25 °C) of (*M*)-$\mathbf{1}_{\mathrm{TBA2}}$ before (i) and after (ii) heating at 80 °C for 24 h in the presence of MS-4A. (**b**,**c**) Plots of the time-dependent CD intensity changes at 423 nm of (*M*)-$\mathbf{1}_{\mathrm{TBA2}}$ (2.0 μM) at 70, 80, 90, 100 and 110 °C in DMSO containing 1.8 ppm H_2O: [H_2O]/[(*M*)-$\mathbf{1}_{\mathrm{TBA2}}$] = ca. 500. The time-dependent CD measurement was performed individually three times, because the kinetic and thermodynamic parameters for the water-mediated racemization of the helicate was sensitive to a subtle change in water content in a DMSO solution of (*M*)-$\mathbf{1}_{\mathrm{TBA2}}$. One of the three experimental results is shown. (**d**) Eyring and (**e**) Arrhenius plots for the racemization of (*M*)-$\mathbf{1}_{\mathrm{TBA2}}$ in DMSO containing 1.8 ppm H_2O: [H_2O]/[$\mathbf{1}_{\mathrm{TBA2}}$] = ca. 500. The obtained kinetic and thermodynamic parameters are summarized in Supplementary Table 1. (**f**) Schematic representation of a possible pathway for the water-mediated racemization of $\mathbf{1}_{\mathrm{TBA2}}$ through simultaneous cleavages of one of the four B–O bonds at each spiroborate group followed by reformation of the spiroborated (*P*)- or (*M*)-helicate. Source data of (**b-e**) are provided as a Source Data file.

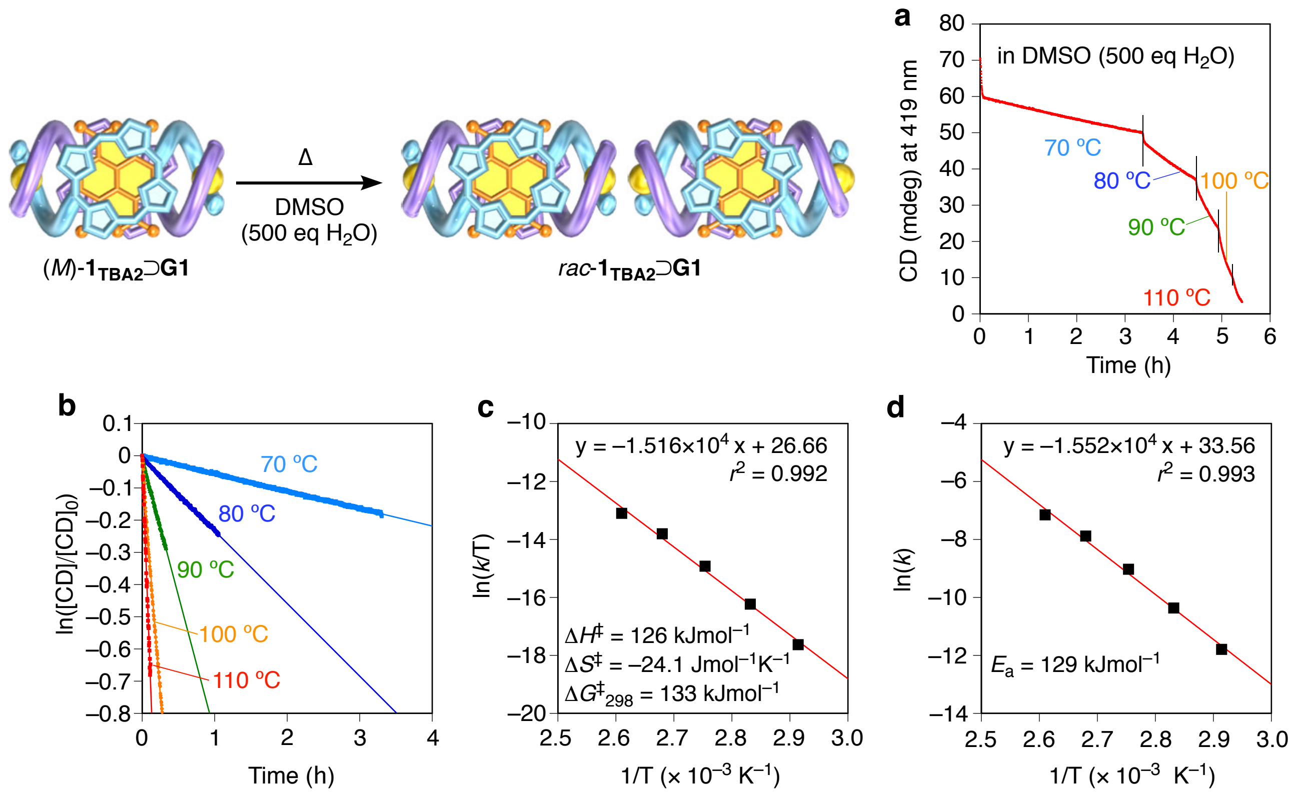

Supplementary Figure 2 | Kinetics and thermodynamics of water-mediated racemization of the inclusion complex (*M*)-$\mathbf{1_{TBA2}}$⊃G1. (**a**,**b**) Plots of the time-dependent CD intensity changes at 419 nm of (*M*)-$\mathbf{1_{TBA2}}$ (2.0 μM) in the presence of 10 equivalents of **G1** at 70, 80, 90, 100 and 110 °C in DMSO containing 1.8 ppm H_2O: [H_2O]/[(*M*)-$\mathbf{1_{TBA2}}$] = ca. 500. The time-dependent CD measurement was performed individually three times, because the kinetic and thermodynamic parameters for the water-mediated racemization of the helicate was sensitive to a subtle change in water content in a DMSO solution of (*M*)-$\mathbf{1_{TBA2}}$. One of the three experimental results is shown. (**c**) Eyring and (**d**) Arrhenius plots for the racemization of (*M*)-$\mathbf{1_{TBA2}}$ in the presence of 10 equivalents of **G1** in DMSO containing 1.8 ppm H_2O: [H_2O]/[(*M*)-$\mathbf{1_{TBA2}}$] = ca. 500. The obtained kinetic and thermodynamic parameters are summarized in Supplementary Table 1. Source data are provided as a Source Data file.

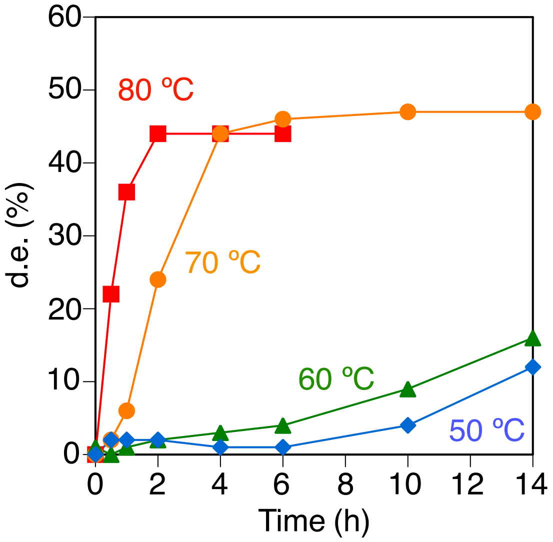

Supplementary Figure 3 | Deracemization of *rac*-$\mathbf{1}_{\mathbf{Na2}}$ upon inclusion complexation with (*S*)-G2 in DMSO-*d*$_6$ at various temperatures. Plots of d.e. of *derac*-$\mathbf{1}_{\mathbf{Na2}}$⊃(*S*)-**G2** versus time. Deracemization of *rac*-$\mathbf{1}_{\mathbf{Na2}}$ was performed upon heating of a mixture of *rac*-$\mathbf{1}_{\mathbf{Na2}}$ (0.50 mM) with (*S*)-**G2** ([(*S*)-**G2**]/[$\mathbf{1}_{\mathbf{Na2}}$] = 3) in DMSO-*d*$_6$ at 50–80 ºC. [H_2O]/[(*M*)-$\mathbf{1}_{\mathbf{TBA2}}$] = ca. 13-18. Source data are provided as a Source Data file.

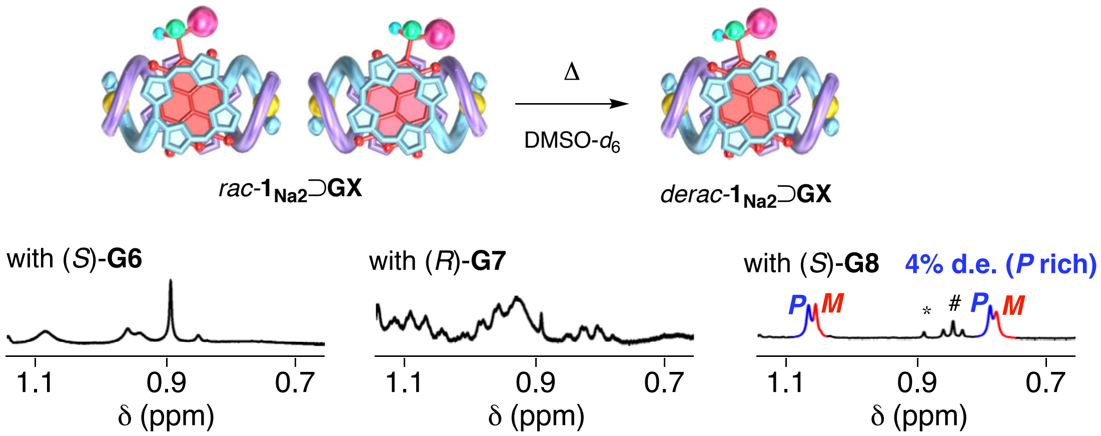

Supplementary Figure 4 | Deracemization of *rac*-$\mathbf{1}_{\mathbf{Na2}}$ upon inclusion complexation with various enantiopure guests in DMSO-*d*$_6$. Partial ^{1}H NMR spectra of mixtures of *rac*-$\mathbf{1}_{\mathbf{Na2}}$ (0.50 mM) with (*S*)-**G6**, (*R*)-**G7** and (*S*)-**G8** ([guest]/[$\mathbf{1}_{\mathbf{Na2}}$] = 3) in DMSO-*d*$_6$ measured at ambient temperature after heating at 80 °C for 24-48 h. [H_2O]/[$\mathbf{1}_{\mathbf{Na2}}$] = ca. 5-150. # and * denote the protons from (*S*)-**G8** and unknown impurities, respectively. For the assignment of (*P*)- and (*M*)-helicity, see the text. The d.e. values of *derac*-$\mathbf{1}_{\mathbf{Na2}}$⊃(*S*)-**G6** and *derac*-$\mathbf{1}_{\mathbf{Na2}}$⊃(*R*)-**G7** could not be determined, because of their complicated ^{1}H NMR spectra.

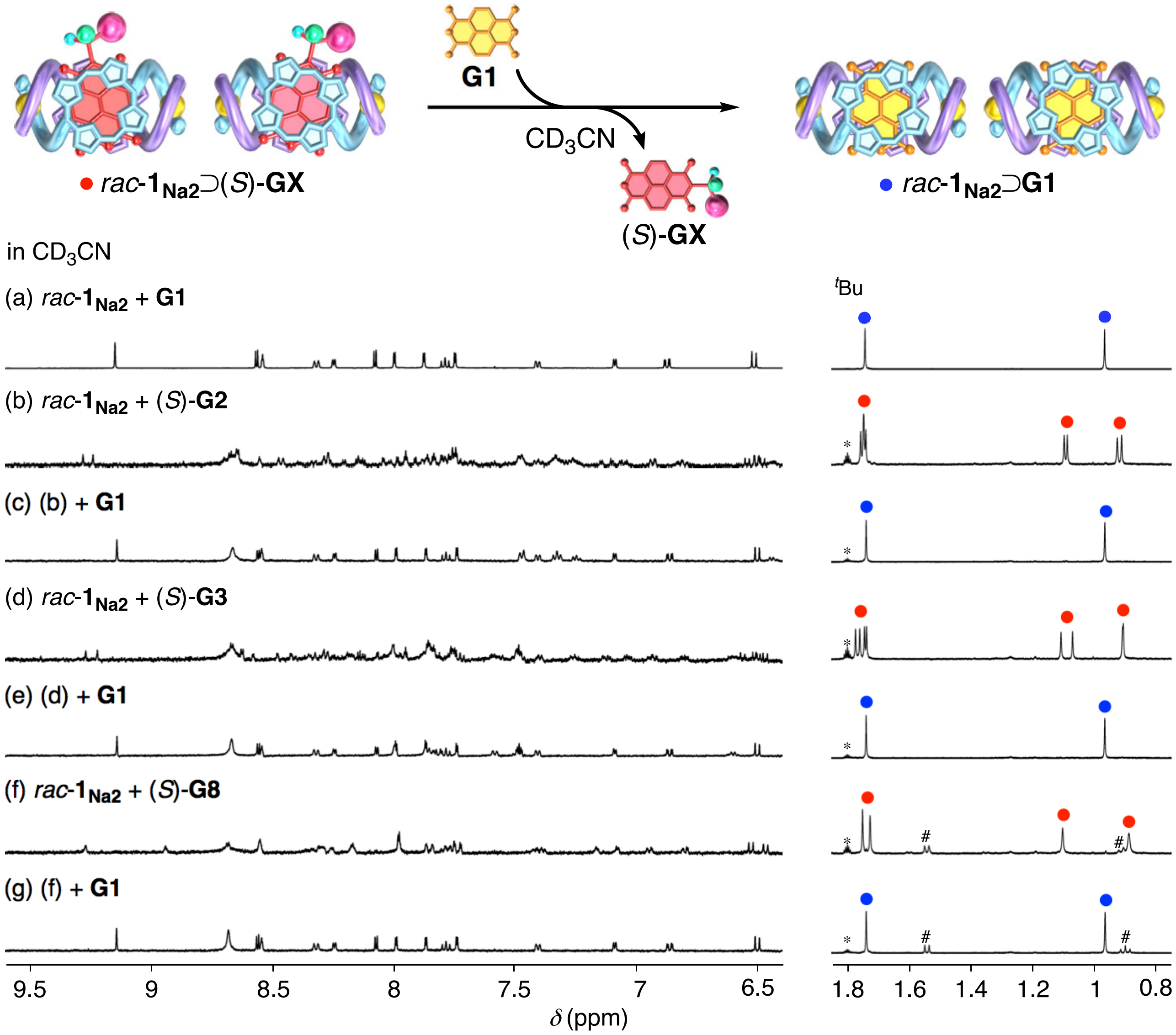

Supplementary Figure 5 | Replacement of chiral guests complexed with *rac*-$\mathbf{1}_{\mathrm{Na2}}$ by achiral guest G1. Partial ^{1}H NMR spectra (500 MHz, 0.40 mM, ambient temperature) of *rac*-$\mathbf{1}_{\mathrm{Na2}}$ in the presence of 1 equivalent of **G1** (a), 3 equivalents of (*S*)-**G2** (b), (b) + 1 equivalent of **G1** (c), 3 equivalents of (*S*)-**G3** (d), (d) + 1 equivalent of **G1** (e), 3 equivalents of (*S*)-**G8** (f) and (f) + 1 equivalent of **G1** (g) in CD_3CN. * and # denote the protons from ^{13}C satellite signal of CD_2HCN and (*S*)-**G8**, respectively.

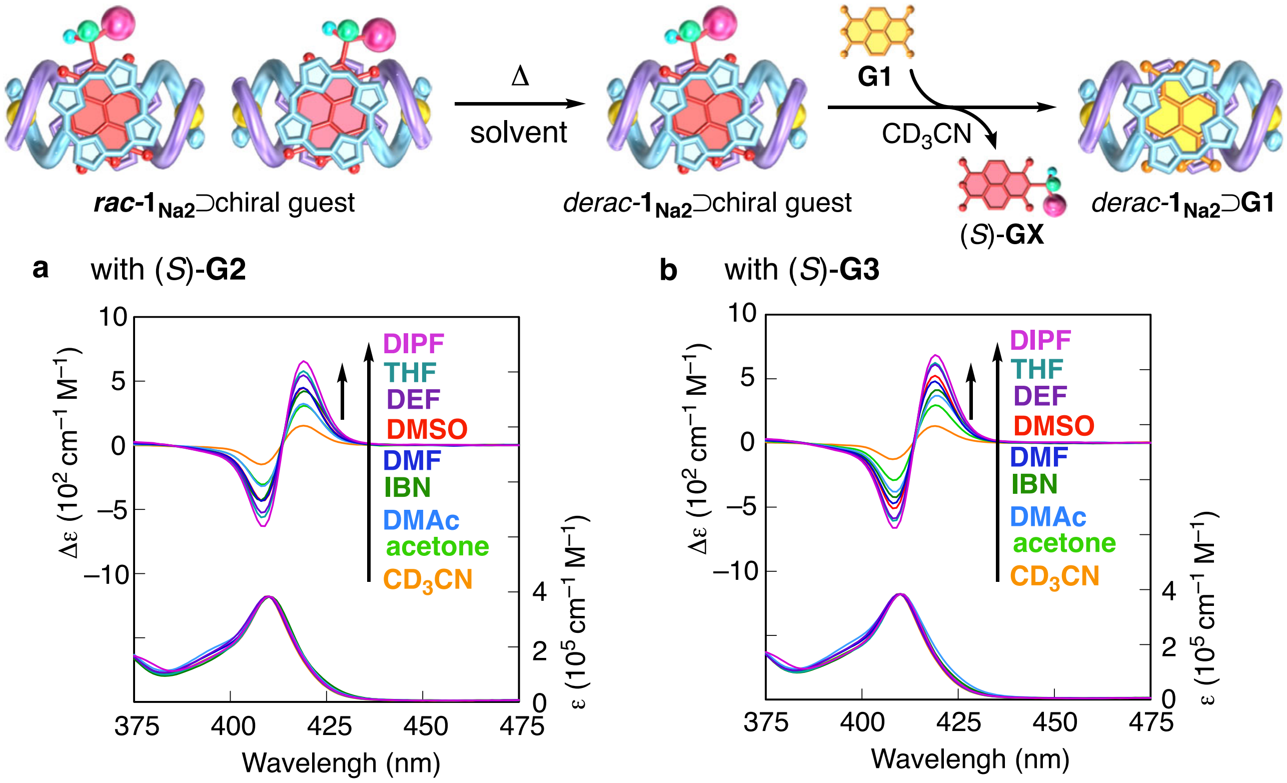

Supplementary Figure 6 | Deracemization of *rac*-$\mathbf{1}_{\mathbf{Na2}}$ upon inclusion complexation with (*S*)-G2 and (*S*)-G3 in various solvents. CD and absorption spectra of $\mathbf{1}_{\mathbf{Na2}}$ (0.20 μM) in the presence of 3 equivalents of achiral **G1** measured in CH_3CN/X (X = various solvents; ca. 29/1, v/v) at 25 °C after deracemization of *rac*-$\mathbf{1}_{\mathbf{Na2}}$ upon inclusion complexation with (*S*)-**G2** (**a**) and (*S*)-**G3** (**b**) ([guest]/[$\mathbf{1}_{\mathbf{Na2}}$] = 3) in various solvents at 80 ºC for 24-266 h. [H_2O]/[$\mathbf{1}_{\mathbf{Na2}}$] = ca. 5-75. The CD measurements were performed according to the following procedures; solutions of *rac*-$\mathbf{1}_{\mathbf{Na2}}$ in the presence of (*S*)-**G2** or (*S*)-**G3** in various solvents ([guest]/[$\mathbf{1}_{\mathbf{Na2}}$] = 3) were heated to 80 °C until reaching an equilibrium state. The solutions were then cooled to ambient temperature and diluted thirtyfold with a CH_3CN solution containing 3 equivalents of achiral **G1**, which completely replaced the (*S*)-**G2** and (*S*)-**G3** complexed with the *derac*-$\mathbf{1}_{\mathbf{Na2}}$, before CD and absorption measurements.

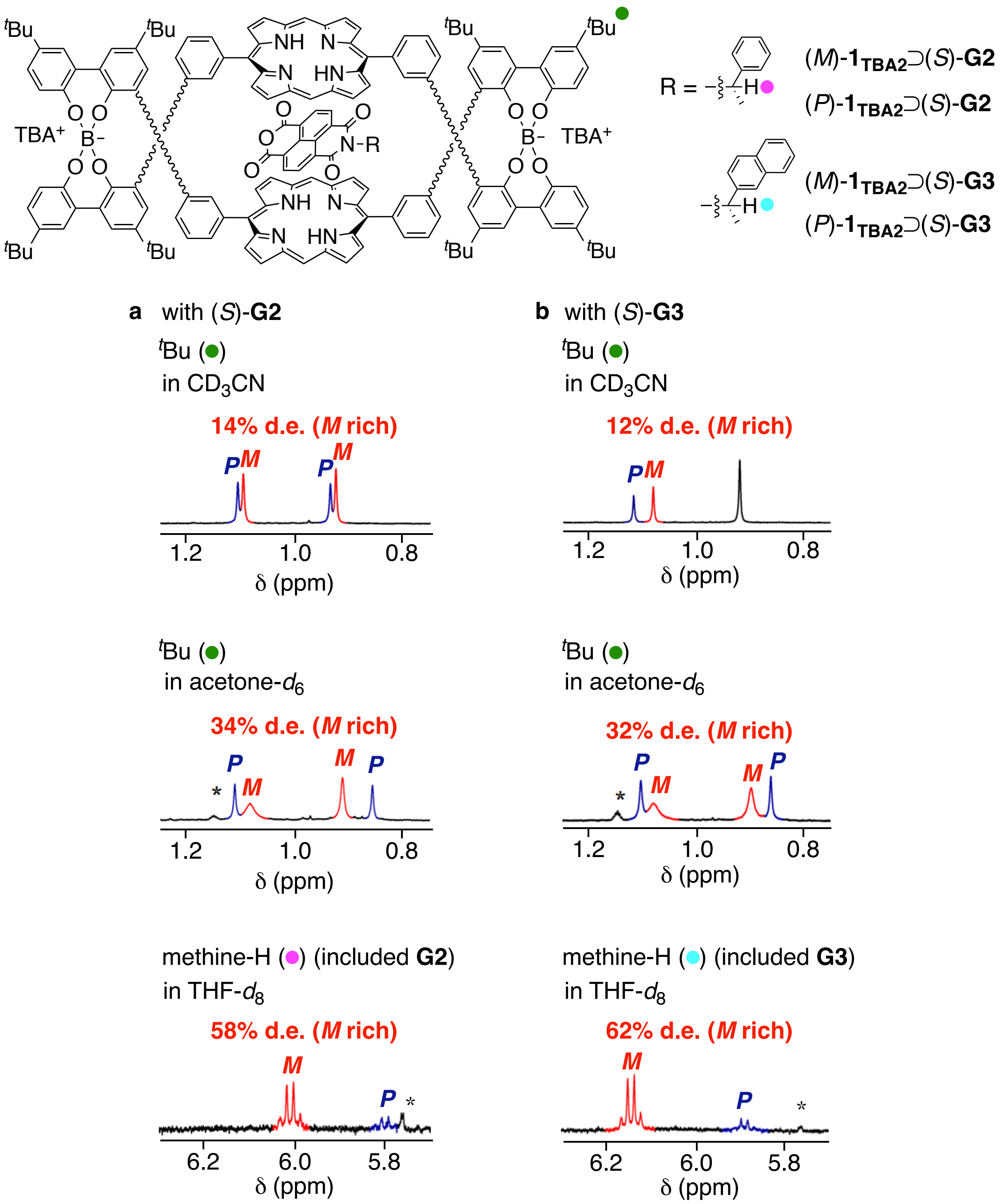

Supplementary Figure 7 | Deracemization of *rac*-$\mathbf{1}_{\mathbf{Na2}}$ upon inclusion complexation with (*S*)-G2 and (*S*)-G3 in various solvents. Partial ^{1}H NMR spectra of mixtures of $\mathbf{1}_{\mathbf{Na2}}$ (0.50 mM) with (*S*)-**G2** (**a**) and (*S*)-**G3** (**b**) ([guest]/[$\mathbf{1}_{\mathbf{Na2}}$] = 3) in CD_3CN, acetone-d_6 and THF-d_8 measured at ambient temperature after heating at 80 °C for 26-266 h. [H_2O]/[(*M*)-$\mathbf{1}_{\mathbf{TBA2}}$] = ca. 5-20. * denotes the protons from unknown impurities.

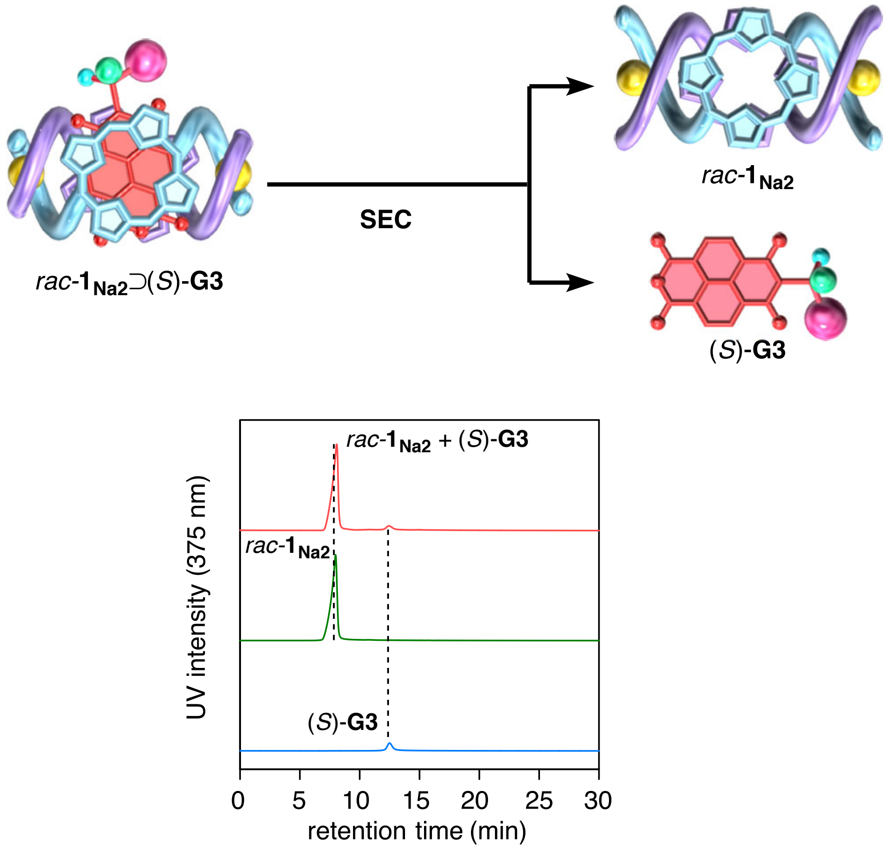

Supplementary Figure 8 | Isolation of *rac*-1_{Na2} and (*S*)-G3 from their inclusion complex *rac*-1_{Na2}⊃(*S*)-G3 by SEC. SEC chromatogram of the separation of the *rac*-$\mathbf{1}_{Na2}$⊃(*S*)-**G3** (top) and those of *rac*-$\mathbf{1}_{Na2}$ (middle) and (*S*)-**G3** (bottom). SEC conditions: column, TSKgel α 2500 (TOSOH, 0.78 (i.d.) × 30 cm); eluent, DMF; flow rate, 0.6 mL/min.

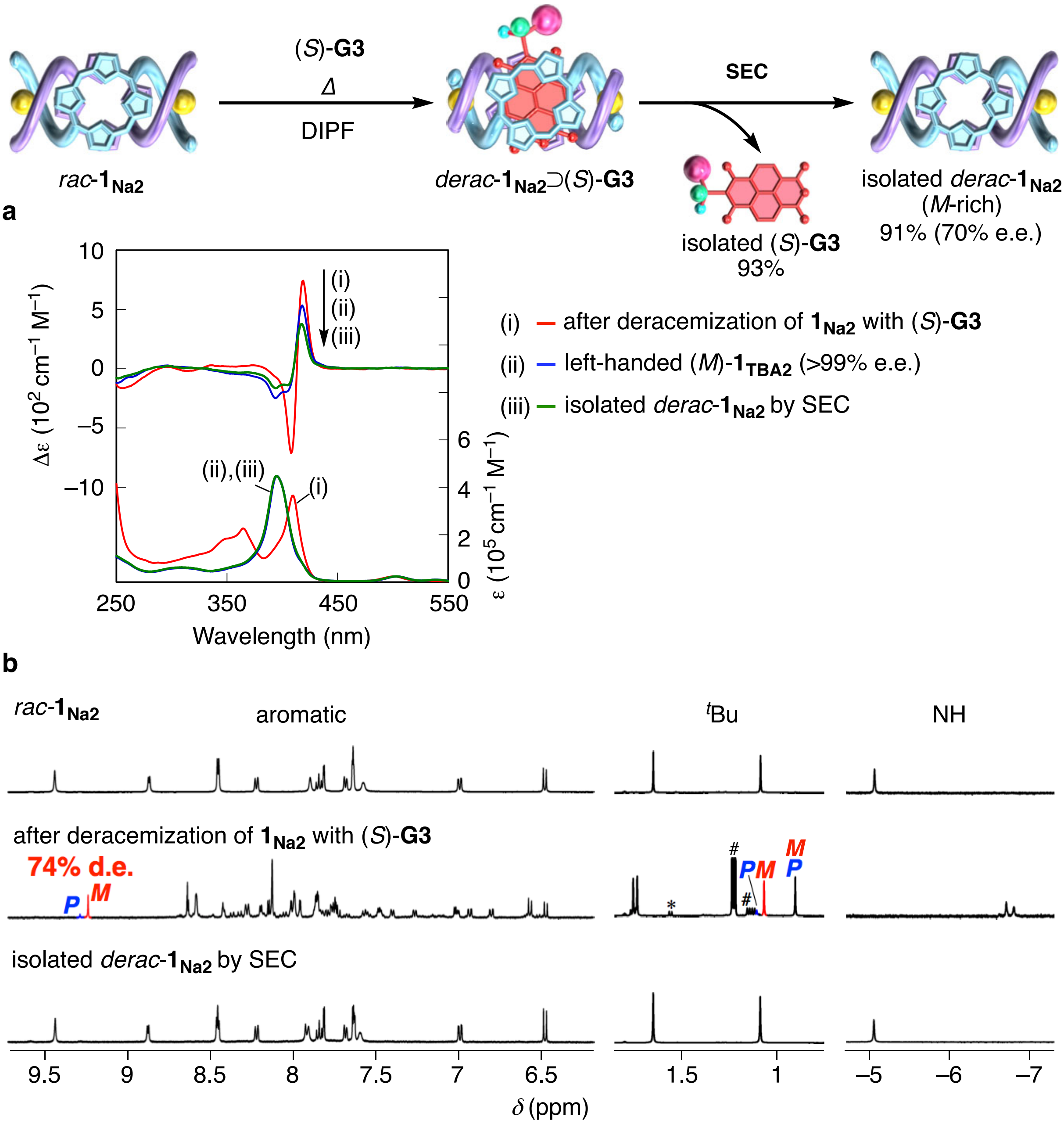

Supplementary Figure 9 | Isolation of optically active *derac*-$\mathbf{1}_{\mathrm{Na2}}$ (*M*-rich) and (*S*)-G3 by SEC after deracemization of *rac*-$\mathbf{1}_{\mathrm{Na2}}$ with (*S*)-G3. (**a**) CD and absorption spectra (0.50 mM) of the *derac*-$\mathbf{1}_{\mathrm{Na2}}$⊃(*S*)-**G3** complex ([(*S*)-**G3**]/[$\mathbf{1}_{\mathrm{Na2}}$] = 3) after deracemization of *rac*-$\mathbf{1}_{\mathrm{Na2}}$ in DIPF at 80 °C for 24 h, the isolated optically active $\mathbf{1}_{\mathrm{Na2}}$ by SEC, and enantiopure left-handed (*M*)-$\mathbf{1}_{\mathrm{TBA2}}$ measured in CH_3CN at 25 ºC. (**b**) Partial ^{1}H NMR spectra of *rac*-$\mathbf{1}_{\mathrm{Na2}}$, the *derac*-$\mathbf{1}_{\mathrm{Na2}}$⊃(*S*)-**G3** complex ([(*S*)-**G3**]/[$\mathbf{1}_{\mathrm{Na2}}$] = 3) after deracemization and the isolated *derac*-$\mathbf{1}_{\mathrm{Na2}}$ by SEC measured in CD_3CN at 25 ºC. * and # denote the protons from included (*S*)-**G3** and DIPF, respectively. The CD, absorption and NMR measurements were performed according to the following procedure; a DIPF solution of *rac*-$\mathbf{1}_{\mathrm{Na2}}$ in the presence of (*S*)-**G3** ([(*S*)-**G3**]/[$\mathbf{1}_{\mathrm{Na2}}$] = 3) was heated to 80 °C. After 24 h, the solvent was evaporated and the *derac*-$\mathbf{1}_{\mathrm{Na2}}$ and (*S*)-**G3** were isolated by SEC using an SEC column (TOYOPEARL HW-40S; 1.8 (i.d.) × 25 cm) with DMF as the eluent.

Determination of the Association Constants of (*M*)-1_{TBA2} with (*S*)- and (*R*)-G2 in THF and CH_3CN.

$$(M)\text{-}\mathbf{1}_{\mathbf{TBA2}} + (S)\text{-}\mathbf{G2} \underset{\text{THF}}{\overset{K_a}{\rightleftharpoons}} (M)\text{-}\mathbf{1}_{\mathbf{TBA2}}\supset(S)\text{-}\mathbf{G2} \qquad K_a = \frac{[(M)\text{-}\mathbf{1}_{\mathbf{TBA2}}\supset(S)\text{-}\mathbf{G2}]}{[(M)\text{-}\mathbf{1}_{\mathbf{TBA2}}]\,[(S)\text{-}\mathbf{G2}]}$$

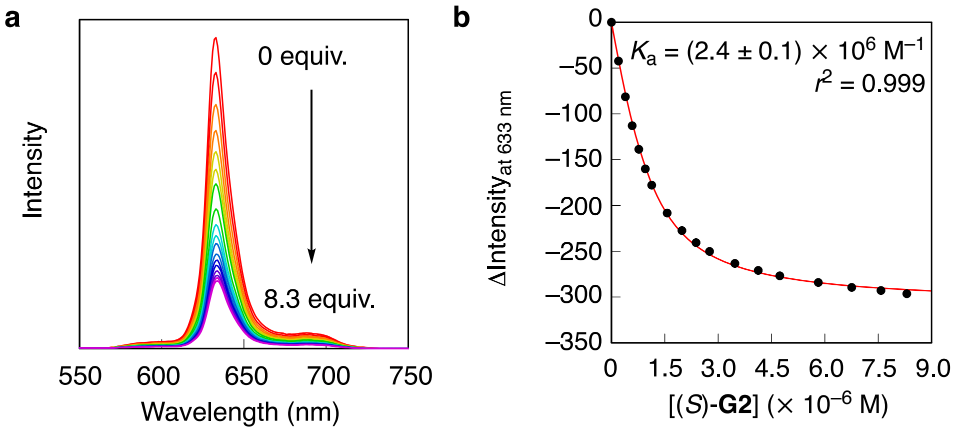

$$(M)\text{-}\mathbf{1}_{\mathbf{TBA2}} + (R)\text{-}\mathbf{G2} \underset{\text{THF}}{\overset{K_a}{\rightleftharpoons}} (M)\text{-}\mathbf{1}_{\mathbf{TBA2}}\supset(R)\text{-}\mathbf{G2} \qquad K_a = \frac{[(M)\text{-}\mathbf{1}_{\mathbf{TBA2}}\supset(R)\text{-}\mathbf{G2}]}{[(M)\text{-}\mathbf{1}_{\mathbf{TBA2}}]\,[(R)\text{-}\mathbf{G2}]}$$

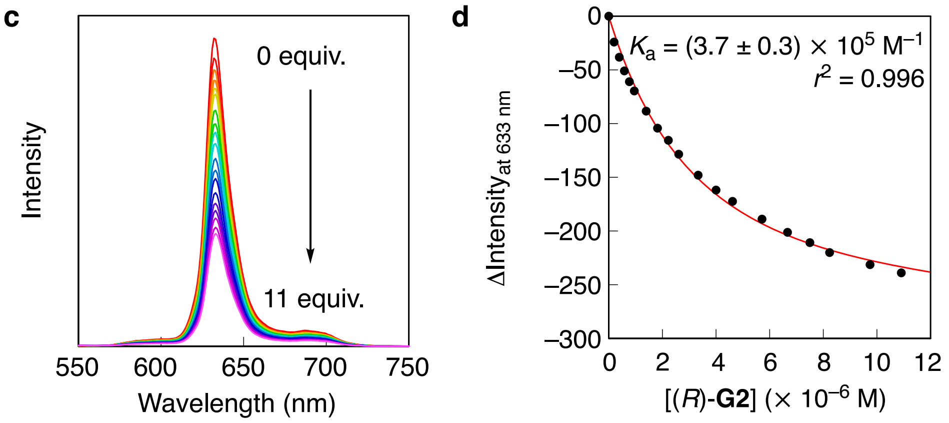

$$\Delta\,\text{Intensity} = I_{obs} - I(M)\text{-}\mathbf{1}_{\mathbf{TBA2}}$$

$$= \frac{(I(M)\text{-}\mathbf{1}_{\mathbf{TBA2}}\supset\mathbf{G2} - I(M)\text{-}\mathbf{1}_{\mathbf{TBA2}})}{2K_a[(M)\text{-}\mathbf{1}_{\mathbf{TBA2}}]_0}\,[1+K_a[(M)\text{-}\mathbf{1}_{\mathbf{TBA2}}]_0+K_a[\mathbf{G2}]_0-\{(1+K_a[(M)\text{-}\mathbf{1}_{\mathbf{TBA2}}]_0+K_a[\mathbf{G2}]_0)^2-4K_a^{\,2}[(M)\text{-}\mathbf{1}_{\mathbf{TBA2}}]_0[\mathbf{G2}]_0\}^{1/2}]$$

I_{obs}: observed fluorescent intensity
$I(M)$-$\mathbf{1}_{\mathbf{TBA2}}$: fluorescent intensity for (*M*)-$\mathbf{1}_{\mathbf{TBA2}}$
$I(M)$-$\mathbf{1}_{\mathbf{TBA2}}\supset\mathbf{G2}$: fluorescent intensity for (*M*)-$\mathbf{1}_{\mathbf{TBA2}}\supset\mathbf{G2}$
$[(M)\text{-}\mathbf{1}_{\mathbf{TBA2}}]_0$: total concentration of (*M*)-$\mathbf{1}_{\mathbf{TBA2}}$
$[\mathbf{G2}]_0$: total concentration of **G2**
K_a: association constant

(To be continued)

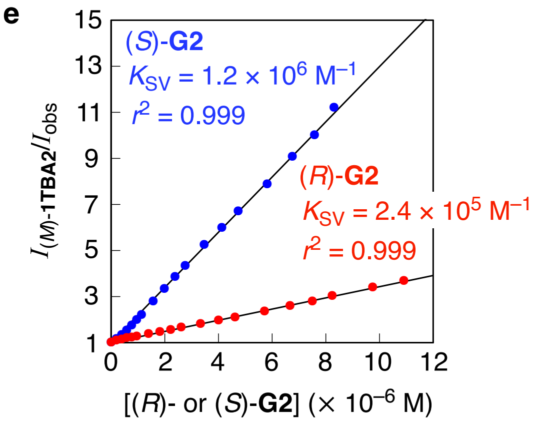

$$\frac{I(M)\text{-}\mathbf{1}_{\mathrm{TBA2}}}{I_{\mathrm{obs}}} = 1 + K_{\mathrm{SV}}[\mathbf{G2}]$$

I_{obs}: observed fluorescent intensity

$I(M)$-$\mathbf{1}_{\mathbf{TBA2}}$: fluorescent intensity for (*M*)-$\mathbf{1}_{\mathbf{TBA2}}$

K_{SV}: Stern-Volmer constant

Supplementary Figure 10 | Diastereoselective inclusion complexation between (*M*)-$\mathbf{1}_{\mathbf{TBA2}}$ and (*S*)- and (*R*)-G2 in THF. (**a**, **c**) Fluorescence spectral changes of (*M*)-$\mathbf{1}_{\mathbf{TBA2}}$ (1.0 μM, λ_{ex} = 395 nm) upon the addition of (*S*)-**G2** (**a**) and (*R*)-**G2** (**c**) in THF at 25 ºC measured in a 1.0-cm quartz cell. (**b**, **d**) Plots of fluorescence intensity changes (Δ_{Int}) at 633 nm of (*M*)-$\mathbf{1}_{\mathbf{TBA2}}$ versus the concentrations of (*S*)-**G2** (**b**) and (*R*)-**G2** (**d**). The curves in the plots were obtained by the least-squares curve-fitting method based on a 1:1 stoichiometry, giving the association constants (K_a) of (*M*)-$\mathbf{1}_{\mathbf{TBA2}}$ with (*S*)-**G2** and (*R*)-**G2** to be (2.4 ± 0.1) × 10^6 M^{-1} and (3.7 ± 0.3) × 10^5 M^{-1}, respectively, using the equation above. (**e**) Stern-Volmer plots of (*M*)-$\mathbf{1}_{\mathbf{TBA2}}$ (1.0 μM, λ_{ex} = 395 nm) quenched by (*S*)- and (*R*)-**G2** in THF at 25 ºC (data are taken from a and c). The residual fluorescence of the inclusion complex obtained in the presence of a large excess of **G2** (150 equiv) in THF was not negligible and was subtracted from the fluorescence spectra (a and c). The complete linear plots obtained by the least-squares curve fitting method indicated that the static quenching process of (*M*)-$\mathbf{1}_{\mathbf{TBA2}}$ by (*S*)- and (*R*)-**G2** in THF was dominant as anticipated from the ^{1}H NMR spectra, in which the signals due to free and complexed **G2** were separately observed because of slow exchange between them (Supplementary Fig. 12d). Source data of (**b** and **d**) are provided as a Source Data file.

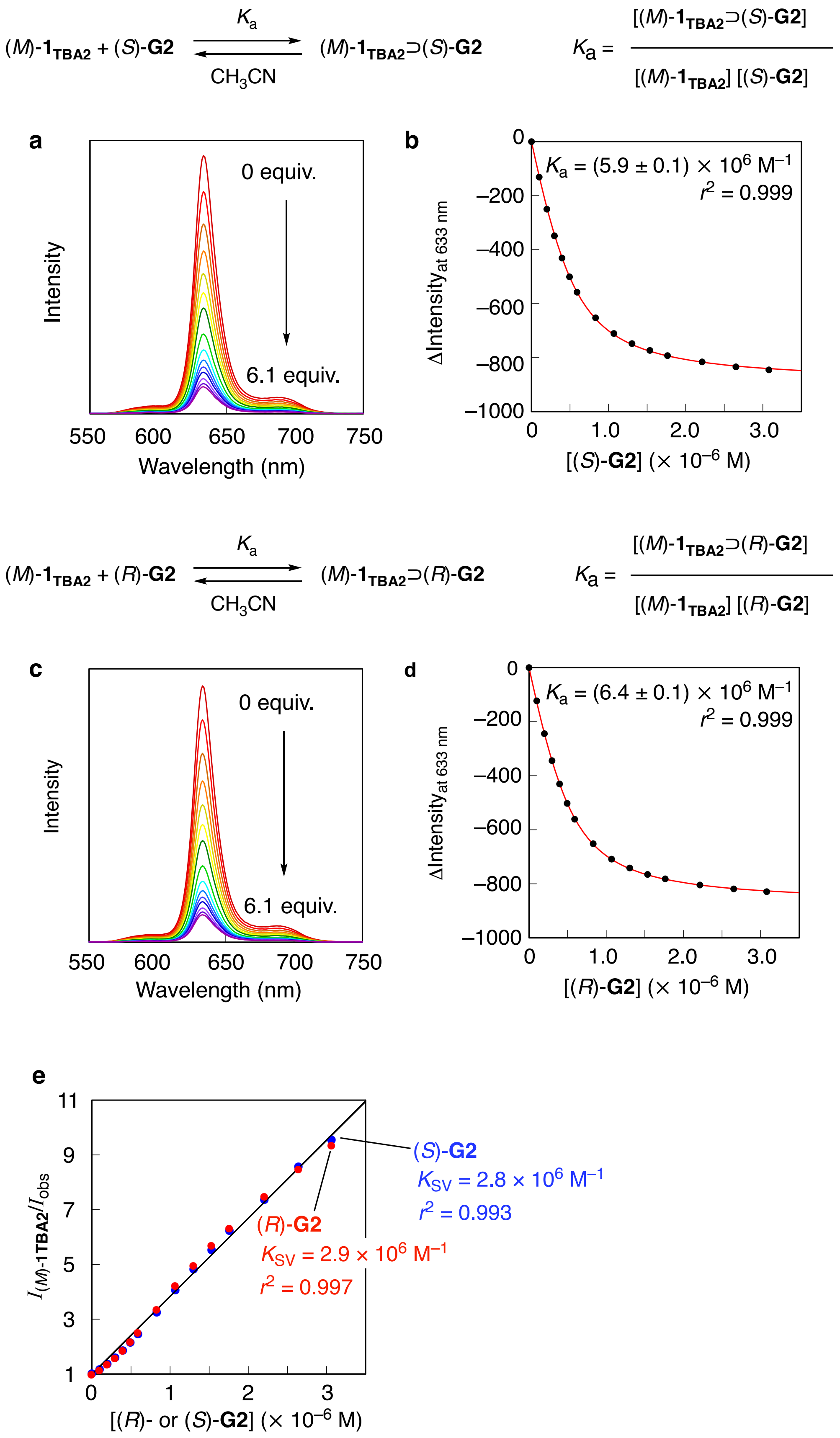
(M)-1TBA2 + (S)-G2
Ka
CH3CN
(M)-1TBA2⊃(S)-G2
Ka =
[(M)-1TBA2⊃(S)-G2]
[(M)-1TBA2] [(S)-G2]
a
0 equiv.
6.1 equiv.
Intensity
550
600
650
700
750
Wavelength (nm)
b
Ka = (5.9 ± 0.1) × 10^6 M^−1
r^2 = 0.999
ΔIntensityat 633 nm
0
−200
−400
−600
−800
−1000
0
1.0
2.0
3.0
[(S)-G2] (× 10^−6 M)
(M)-1TBA2 + (R)-G2
Ka
CH3CN
(M)-1TBA2⊃(R)-G2
Ka =
[(M)-1TBA2⊃(R)-G2]
[(M)-1TBA2] [(R)-G2]
c
0 equiv.
6.1 equiv.
Intensity
550
600
650
700
750
Wavelength (nm)
d
Ka = (6.4 ± 0.1) × 10^6 M^−1
r^2 = 0.999
ΔIntensityat 633 nm
0
−200
−400
−600
−800
−1000
0
1.0
2.0
3.0
[(R)-G2] (× 10^−6 M)
e
I(M)-1TBA2/Iobs
11
9
7
5
3
1
0
1
2
3
[(R)- or (S)-G2] (× 10^−6 M)
(S)-G2
KSV = 2.8 × 10^6 M^−1
r^2 = 0.993
(R)-G2
KSV = 2.9 × 10^6 M^−1
r^2 = 0.997

Supplementary Figure 11 | Diastereoselective inclusion complexation between (*M*)-$\mathbf{1_{TBA2}}$ and (*S*)- and (*R*)-G2 in CH_3CN. (**a**, **c**) Fluorescence spectral changes of (*M*)-$\mathbf{1_{TBA2}}$ (0.50 μM, λ_{ex} = 395 nm) upon the addition of (*S*)-**G2** (**a**) and (*R*)-**G2** (**c**) in CH_3CN at 25 °C measured in a 1.0-cm quartz cell. (**b**, **d**) Plots of fluorescence intensity changes (Δ_{Int}) at 633 nm of (*M*)-$\mathbf{1_{TBA2}}$ versus the concentrations of (*S*)-**G2** (**b**) and (*R*)-**G2** (**d**). The curves in the plots were obtained by the least-squares curve-fitting method based on a 1:1 stoichiometry, giving the association constants (K_a) of (*M*)-$\mathbf{1_{TBA2}}$ with (*S*)-**G2** and (*R*)-**G2** to be (5.9 ± 0.1) × 10^6 M^{-1} and (6.4 ± 0.1) × 10^6 M^{-1}, respectively, using the equation shown in Supplementary Fig. 10. (**e**) Stern-Volmer plots of (*M*)-$\mathbf{1_{TBA2}}$ (0.50 μM, λ_{ex} = 395 nm) quenched by (*S*)- and (*R*)-**G2** in CH_3CN at 25 °C (data are taken from a and c). The residual fluorescence of the inclusion complex obtained in the presence of a large excess of **G2** (150 equiv) in CH_3CN was negligibly small. The almost complete linear plots obtained by the least-squares curve fitting method indicated that the static quenching process of (*M*)-$\mathbf{1_{TBA2}}$ by (*S*)- and (*R*)-**G2** in CH_3CN was dominant as anticipated from the ^{1}H NMR spectra, in which the signals due to free and complexed **G2** were separately observed because of slow exchange between them (Supplementary Fig. 12e). Source data of (**b** and **d**) are provided as a Source Data file.

Diastereoselective Inclusion Complexation of Racemic Guests in (*M*)-1$_{TBA2}$.

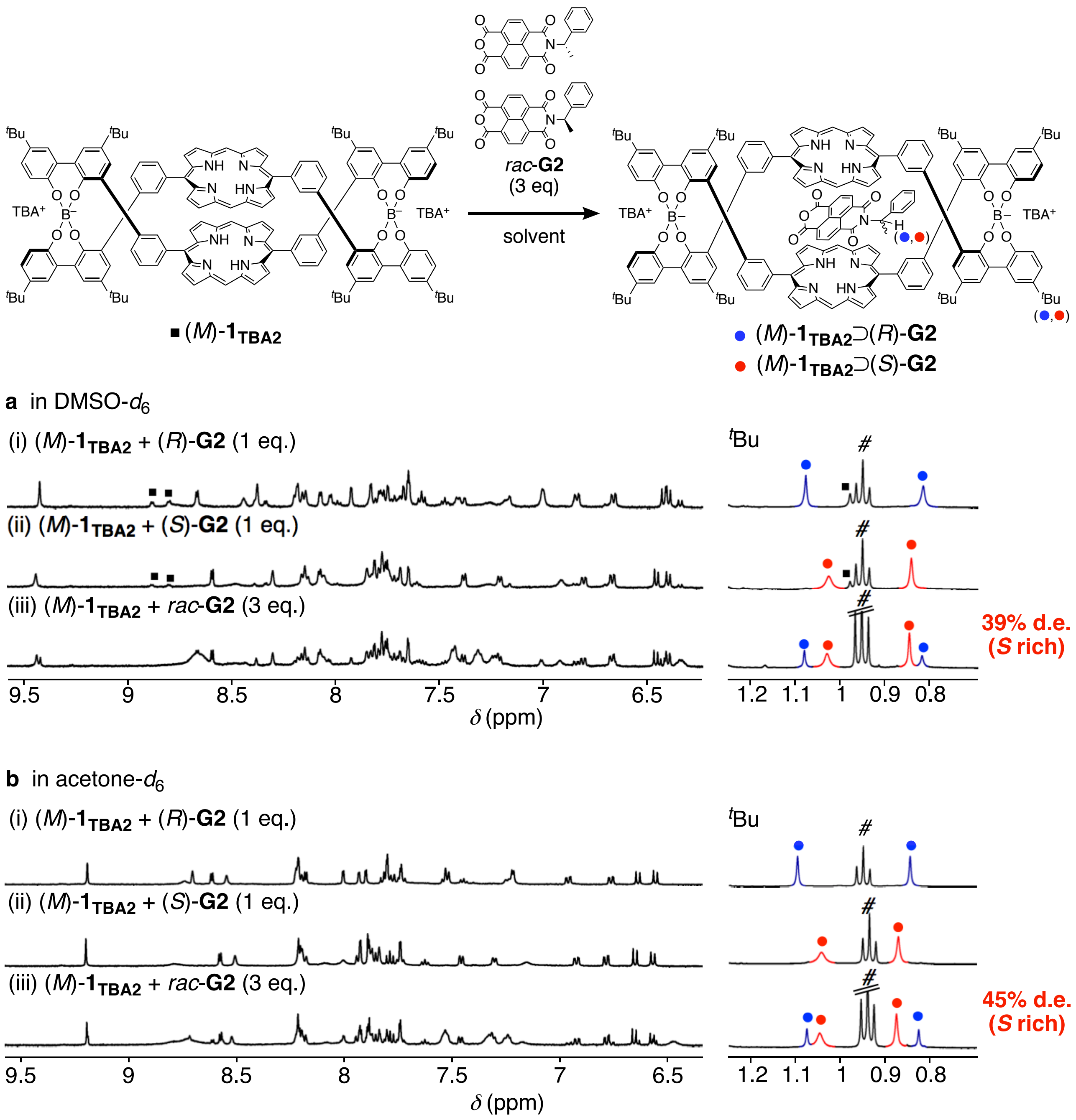

(**Supplementary Figure 12** to be continued)

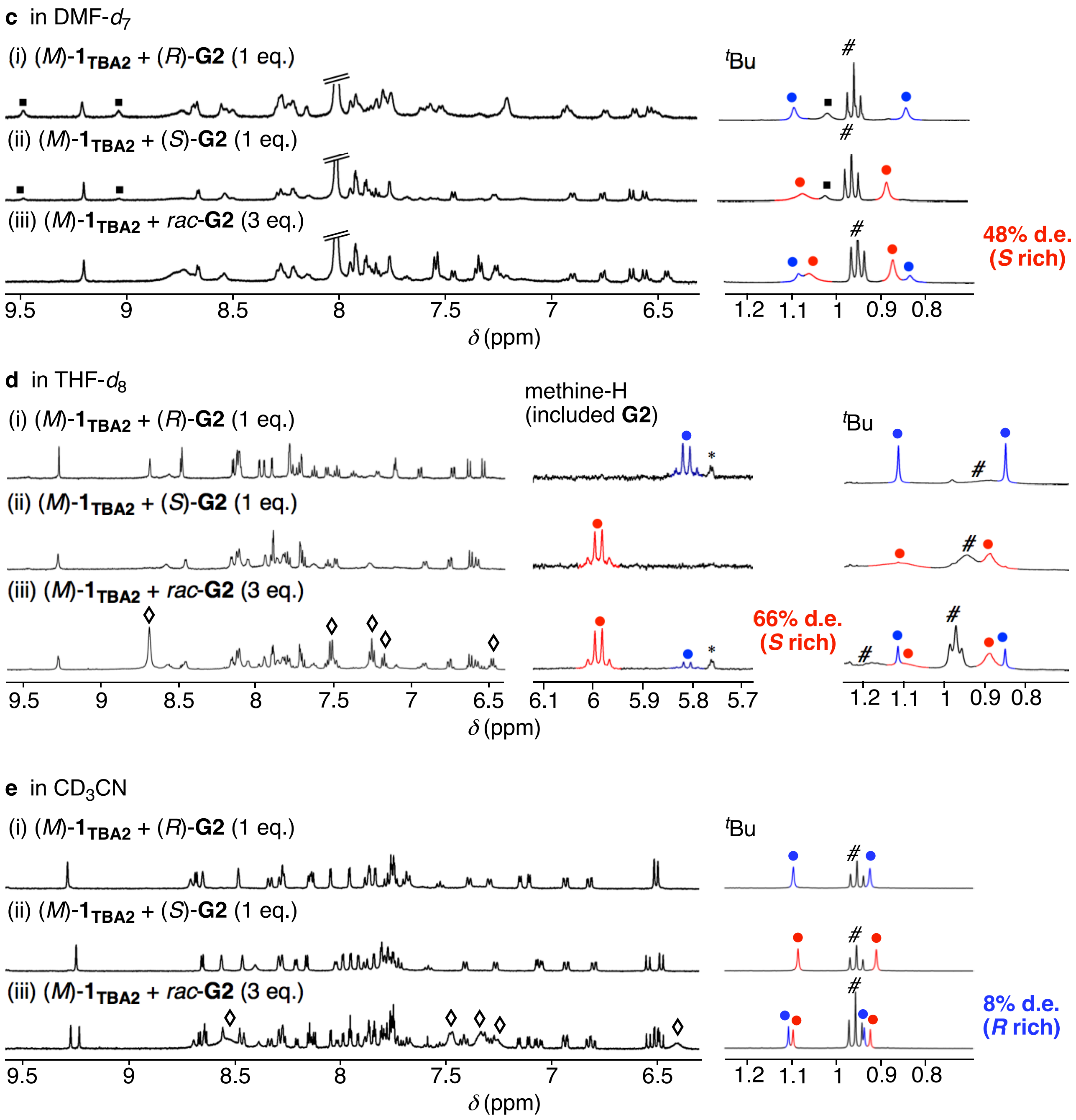

Supplementary Figure 12 | Diastereoselective inclusion complexation between (*M*)-1$_{TBA2}$ and *rac*-G2 in various solvents. Partial ^{1}H NMR spectra (500 MHz, 0.40 mM, 25 °C) of (*M*)-**1**$_{TBA2}$ in the presence of 1 equivalent of (*R*)-**G2** (i) and (*S*)-**G2** (ii), and 3 equivalents of *rac*-**G2** (iii) in DMSO-d_6 (**a**), acetone-d_6 (**b**), DMF-d_7 (**c**), THF-d_8 (**d**) and CD_3CN (**e**). The d.e. values were estimated by the integral ratio of the ^{t}Bu or methine proton signals derived from the diastereomeric inclusion complexes (*M*)-**1**$_{TBA2}$⊃(*R*)-**G2** (blue circles) and (*M*)-**1**$_{TBA2}$⊃(*S*)-**G2** (red circles). #, *, ■ and ◊ denote the protons from TBA, unknown impurities, free (*M*)-**1**$_{TBA2}$ and free **G2**, respectively.

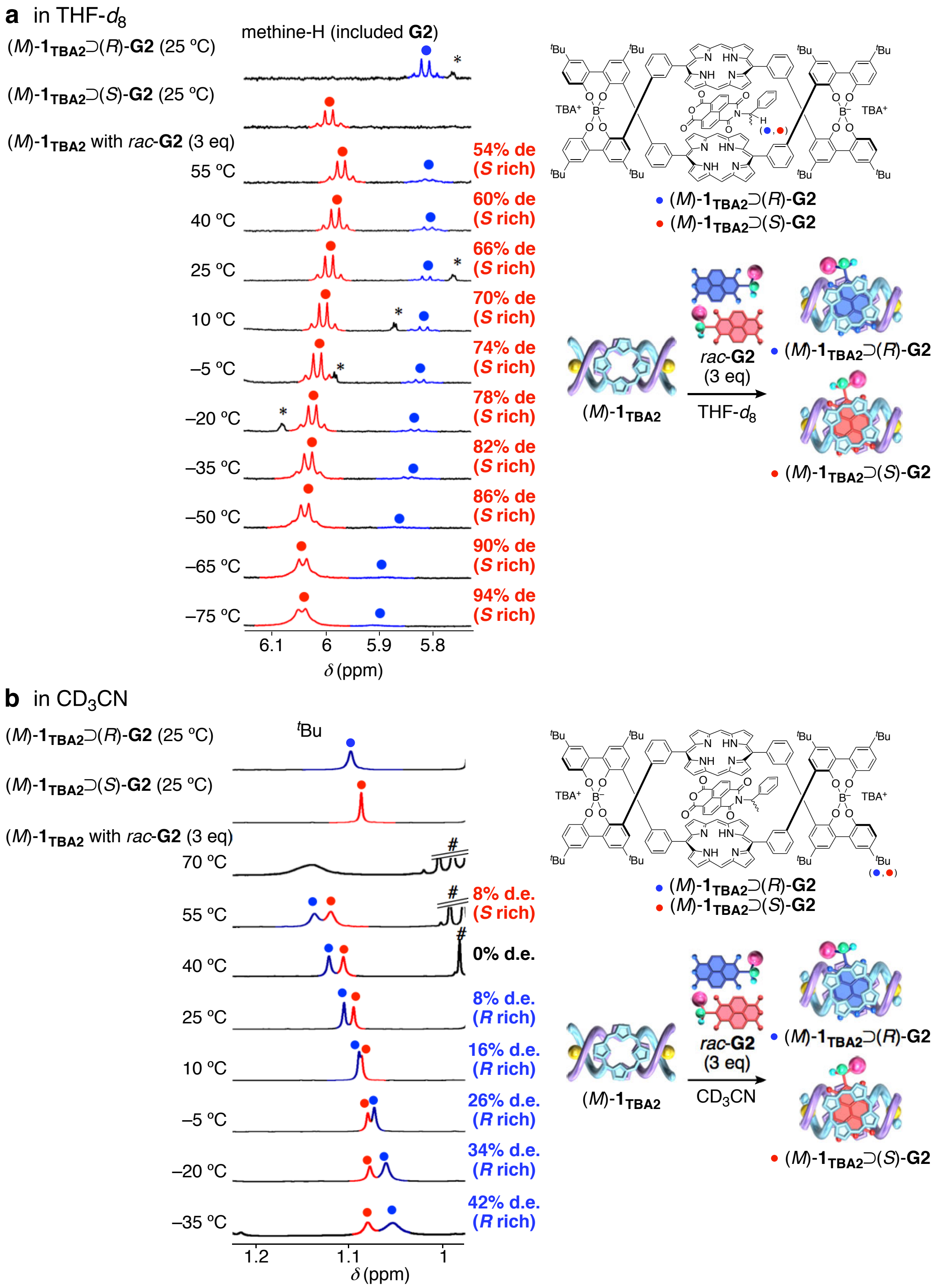

Supplementary Figure 13 | Temperature-dependent diastereoselective inclusion complexation between (*M*)-$\mathbf{1}_{\mathbf{TBA2}}$ and *rac*-G2 in THF-d_8 (a) and CD_3CN (b). Variable temperature (VT) ^{1}H NMR spectra (500 MHz, 0.40 mM) of (*M*)-$\mathbf{1}_{\mathbf{TBA2}}$ in the presence of 3 equivalents of *rac*-**G2** in THF-d_8 from −75 to 55 °C (a) and in CD_3CN from −35 to 70 °C (b). # and * denote the protons from TBA and unknown impurities, respectively. ^{1}H NMR spectra of (*M*)-$\mathbf{1}_{\mathbf{TBA2}}$⊃(*R*)-**G2** (blue circles) and (*M*)-$\mathbf{1}_{\mathbf{TBA2}}$⊃(*S*)-**G2** (red circles) in THF-d_8 (a) and CD_3CN (b) at 25 °C are also shown. The d.e. at 70 ºC in CD_3CN (b) could not be estimated due to the broadening of the peaks of (*M*)-$\mathbf{1}_{\mathbf{TBA2}}$⊃(*R*)-**G2** and (*M*)-$\mathbf{1}_{\mathbf{TBA2}}$⊃(*S*)-**G2**.

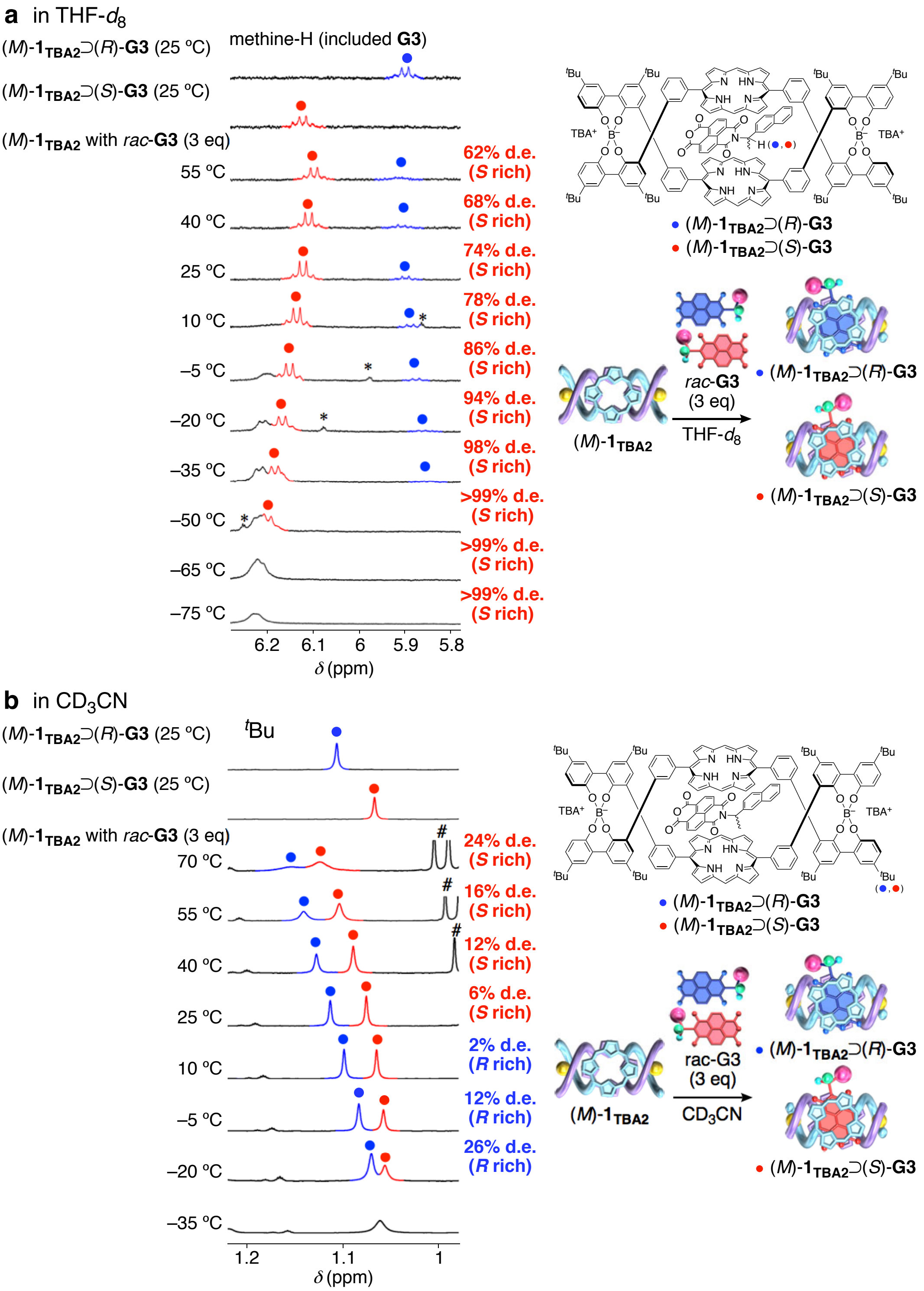

Supplementary Figure 14 | Temperature-dependent diastereoselective inclusion complexation between (*M*)-$\mathbf{1}_{\mathrm{TBA2}}$ and *rac*-G3 in THF-*d*$_8$ (a) and CD_3CN (b). VT ^{1}H NMR spectra (500 MHz, 0.40 mM) of (*M*)-$\mathbf{1}_{\mathrm{TBA2}}$ in the presence of 3 equivalents of *rac*-**G3** in THF-*d*$_8$ from −75 to 55 °C (a) and in CD_3CN from −35 to 70 °C (b). # and * denote the protons from TBA and unknown impurities, respectively. ^{1}H NMR spectra of (*M*)-$\mathbf{1}_{\mathrm{TBA2}}$⊃(*R*)-**G2** (blue circles) and (*M*)-$\mathbf{1}_{\mathrm{TBA2}}$⊃(*S*)-**G2** (red circles) in THF-*d*$_8$ (a) and CD_3CN (b) at 25 °C are also shown. The d.e. at –35 °C in CD_3CN (b) could not be estimated due to the broadening of the peaks of (*M*)-$\mathbf{1}_{\mathrm{TBA2}}$⊃(*R*)-**G3** and (*M*)-$\mathbf{1}_{\mathrm{TBA2}}$⊃(*S*)-**G3**.

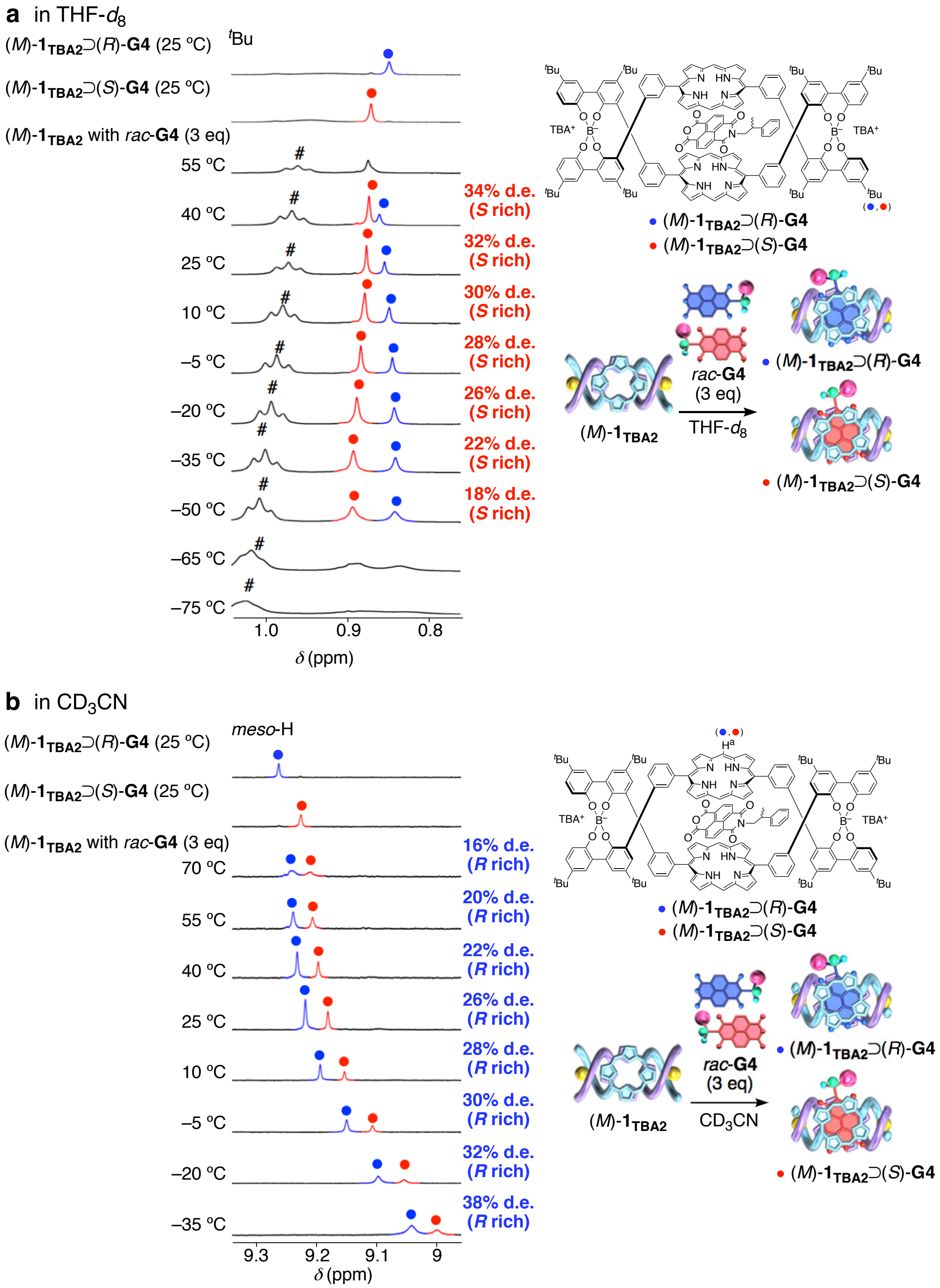

Supplementary Figure 15 | Temperature-dependent diastereoselective inclusion complexation between (*M*)-$\mathbf{1}_{\mathbf{TBA2}}$ and *rac*-G4 in THF-d_8 (a) and CD_3CN (b). VT ^{1}H NMR spectra (500 MHz, 0.40 mM) of (*M*)-$\mathbf{1}_{\mathbf{TBA2}}$ in the presence of 3 equivalents of *rac*-**G4** in THF-d_8 from −75 to 55 °C (a) and in CD_3CN from −35 to 70 °C. # denotes the protons from TBA. ^{1}H NMR spectra of (*M*)-$\mathbf{1}_{\mathbf{TBA2}}$⊃(*R*)-**G4** (blue circles) and (*M*)-$\mathbf{1}_{\mathbf{TBA2}}$⊃(*S*)-**G4** (red circles) in THF-d_8 (a) and CD_3CN (b) at 25 °C are also shown. The d.e. at –65 and –75 ºC in THF-d_8 (a) could not be estimated due to the broadening of the peaks of (*M*)-$\mathbf{1}_{\mathbf{TBA2}}$⊃(*R*)-**G4** and (*M*)-$\mathbf{1}_{\mathbf{TBA2}}$⊃(*S*)-**G4**.

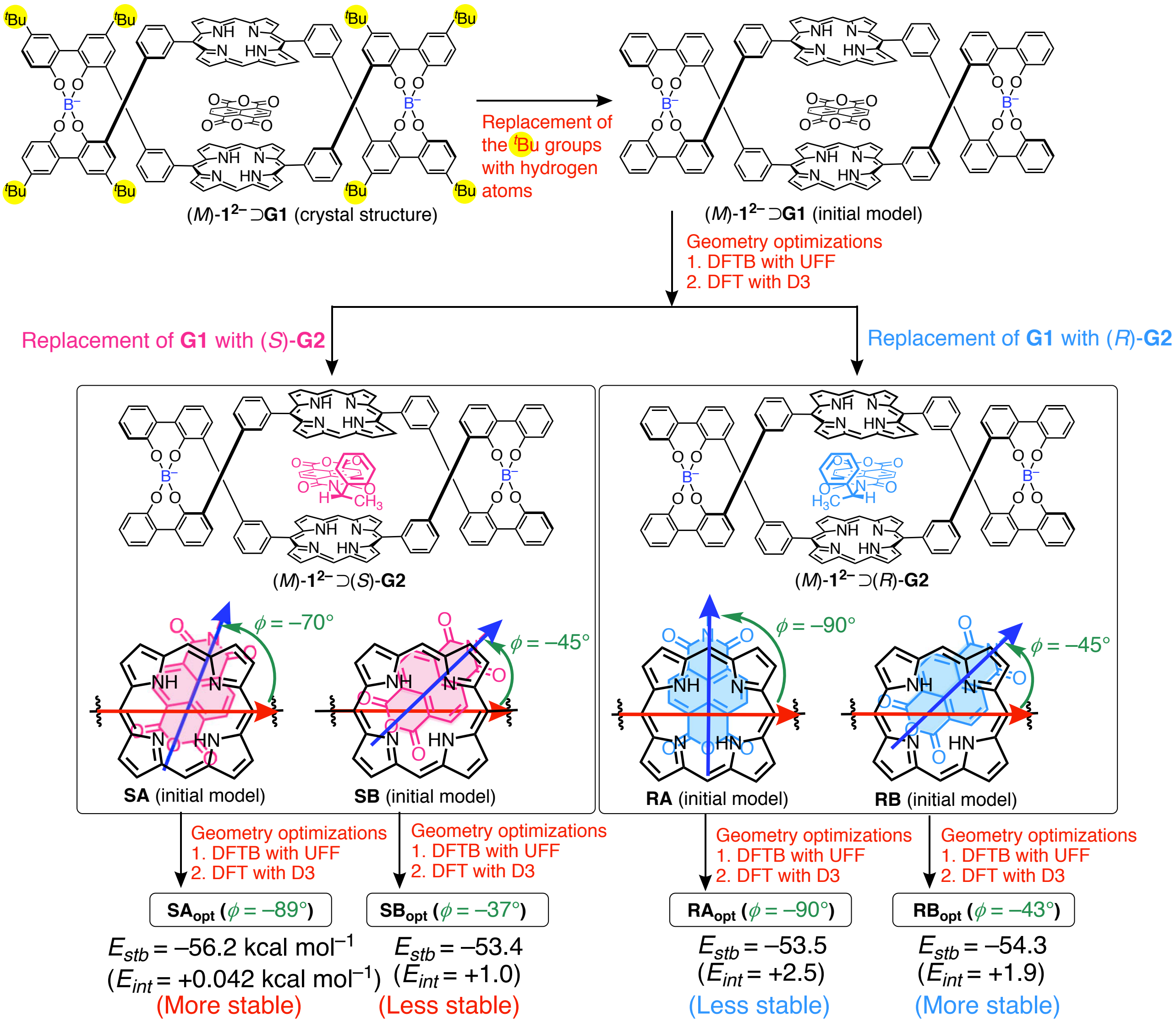

Supplementary Figure 16 | Summary of DFT calculations of diastereomeric pair of (*M*)-1^{2-}⊃G2. Procedures for construction of two different initial structures of each of (*M*)-**1**$^{2-}$⊃(*S*)-**G2** (**SA** and **SB**) and (*M*)-**1**$^{2-}$⊃(*R*)-**G2** (**RA** and **RB**) with different guest orientations and for their geometry optimizations. ϕ denotes the twist angle between the longer axis of the **NMI** moiety of **G2** and the longer axis of one of the two porphyrin rings of (*M*)-**1**$^{2-}$. The E_{stb} and E_{int} values are also shown.

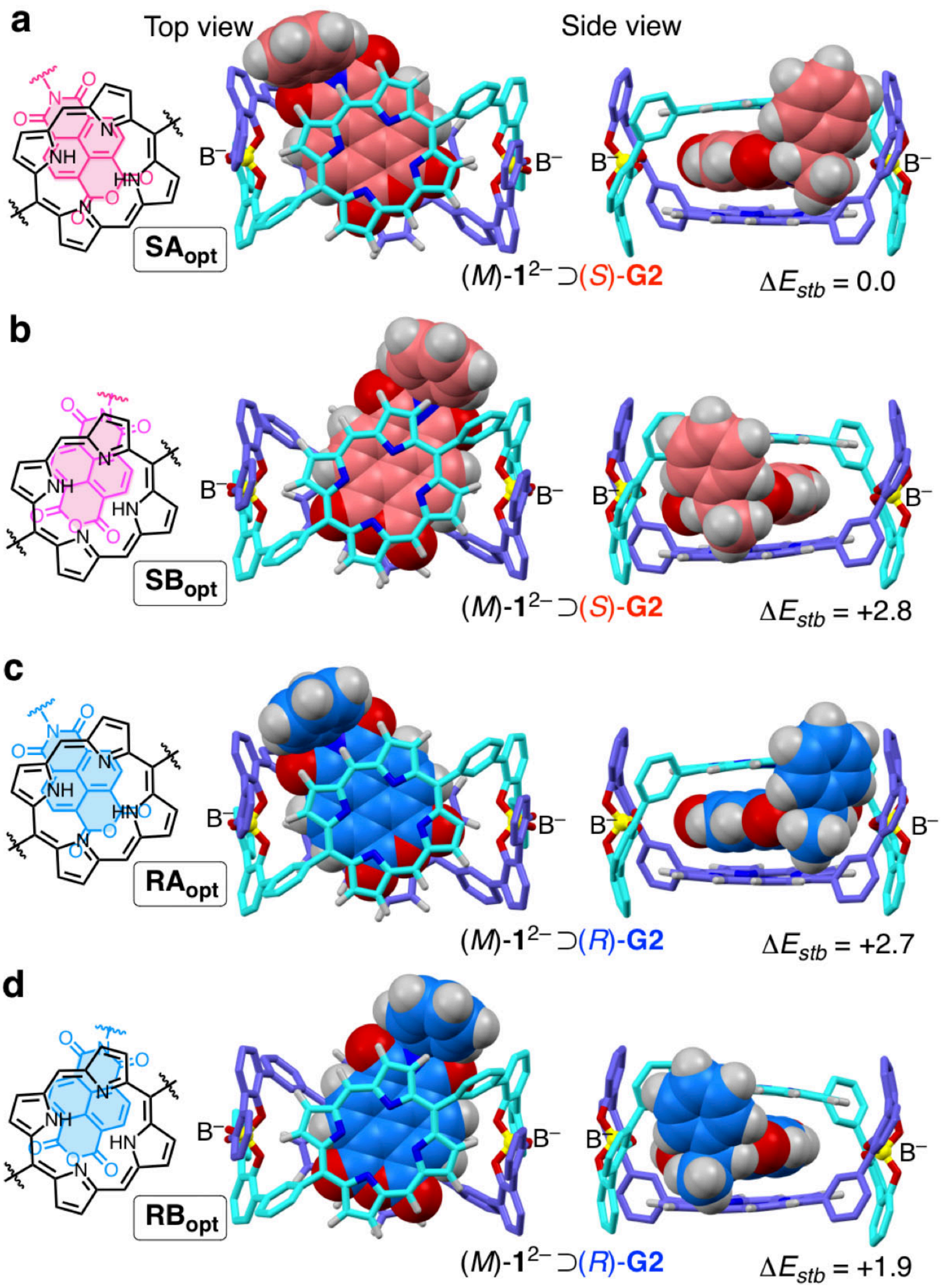

Supplementary Figure 17 | Molecular models of diastereomeric pair of (*M*)-1^{2-}⊃G2. Top and side views of the optimized inclusion complex structures of (*M*)-**1**$^{2-}$⊃(*S*)-**G2** (**SA$_{opt}$** (**a**) and **SB$_{opt}$** (**b**)) and (*M*)-**1**$^{2-}$⊃(*R*)-**G2** (**RA$_{opt}$** (**c**) and **RB$_{opt}$** (**d**)) obtained by the DFT calculations with the D3 dispersion correction. The ΔE_{stb} values are also shown (see Supplementary Table 3). The included guests are highlighted as a space-filling model. Hydrogen atoms except for *meso*- and *β*-protons of the porphyrin rings and **G2** are omitted for clarity. The ^{t}Bu groups were replaced with hydrogen atoms to simplify the calculations.

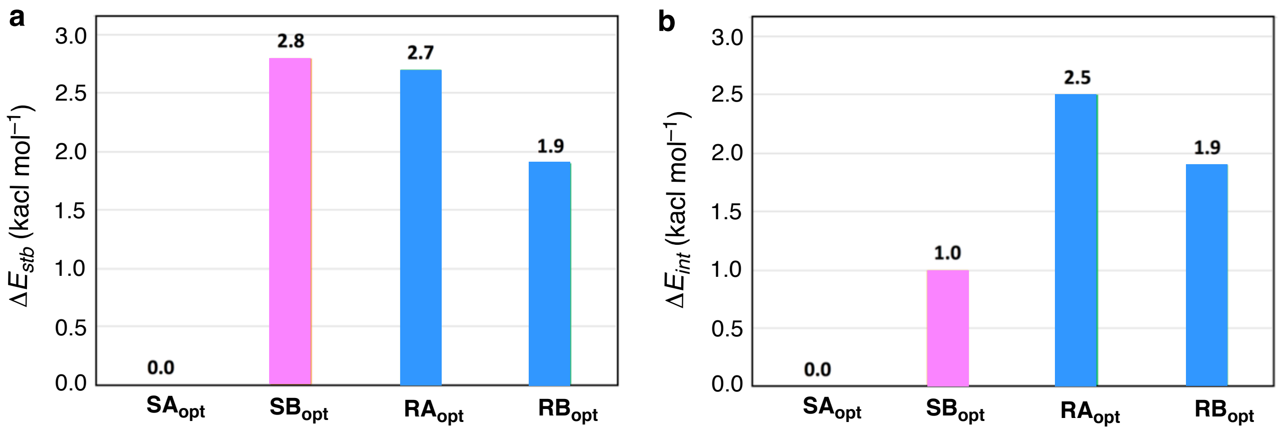

Supplementary Figure 18 | The energy differences (ΔE_{stb} (**a**) and ΔE_{int} (**b**)) between the most stable structure $\mathbf{SA_{opt}}$ and the other structures with the RI-TPSS-D3/def-SV(P) level of theory (**a**) and the RI-TPSS/def-SV(P) level of theory (**b**).

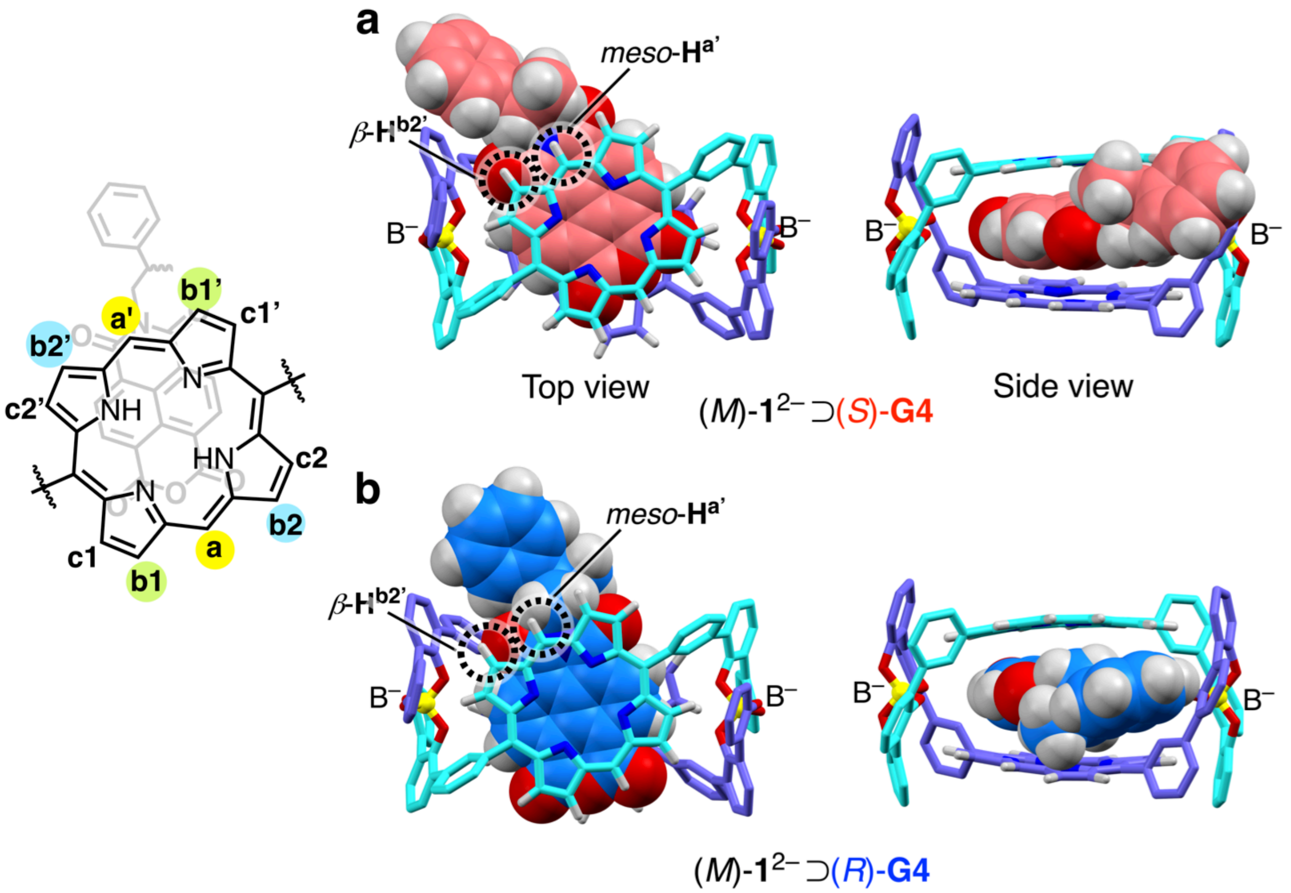

Supplementary Figure 19 | Molecular models of diastereomeric pair of (*M*)-1^{2-}⊃G4. Top and side views of the optimized structures of (*M*)-**1**$^{2-}$⊃(*S*)-**G4** (**a**) and (*M*)-**1**$^{2-}$⊃(*S*)-**G4** (**b**) obtained by the DFT calculations with the D3 dispersion correction. The included guests are highlighted as a space-filling model. Hydrogen atoms except for *meso*- and *β*-protons of the porphyrin rings and **G4** are omitted for clarity. The ^{t}Bu groups were replaced with hydrogen atoms to simplify the calculations.

Rotational Dynamics of the Chiral Pendant Groups of the Guests Included in (*M*)-1_{TBA2}.

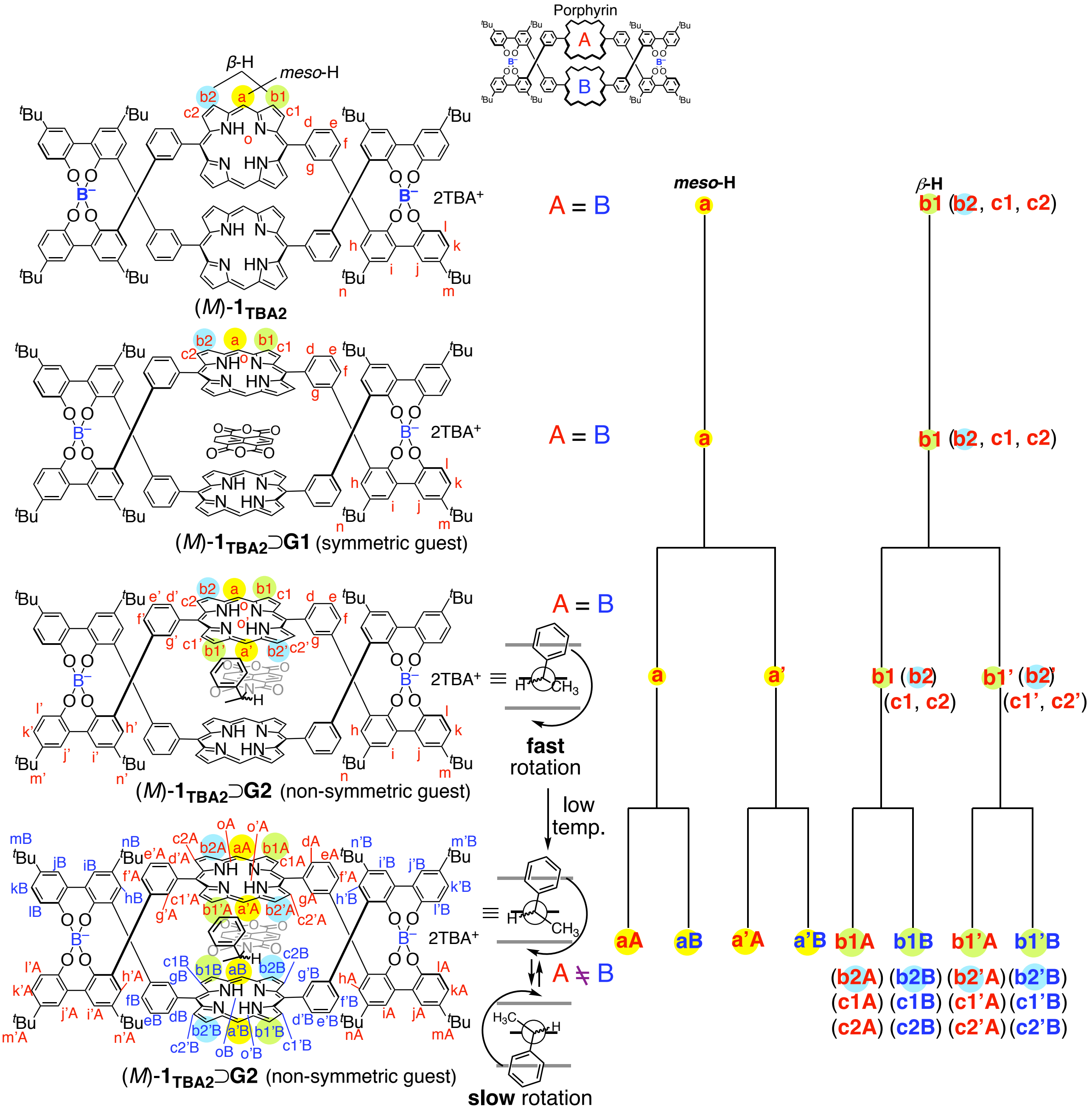

Supplementary Figure 20 | Desymmetrization of a pseudo *D*2-symmetric structure of (*M*)-1_{TBA2} upon complexation with symmetric and non-symmetric guests. Chemical structures of (*M*)-1_{TBA2} and its complexes with symmetric achiral **G1** and non-symmetric chiral **G2** with labels for proton assignments of their NMR spectra shown in Fig. 3b and Supplementary Figs 21–28. Proton peak splitting diagrams for *meso*-H (a and a') and *β*-H (b1 and b1' (b2 and b2', c1 and c1', and c2 and c2')) protons of (*M*)-1_{TBA2} and its inclusion complexes with **G1** and **G2** and further splitting due to slow rotation of the 1-phenylethyl groups around the N-C$^{\alpha}$ bonds (A and B), which results in the formation of two rotational conformers with respect to two (top and bottom) porphyrin rings, are also shown.

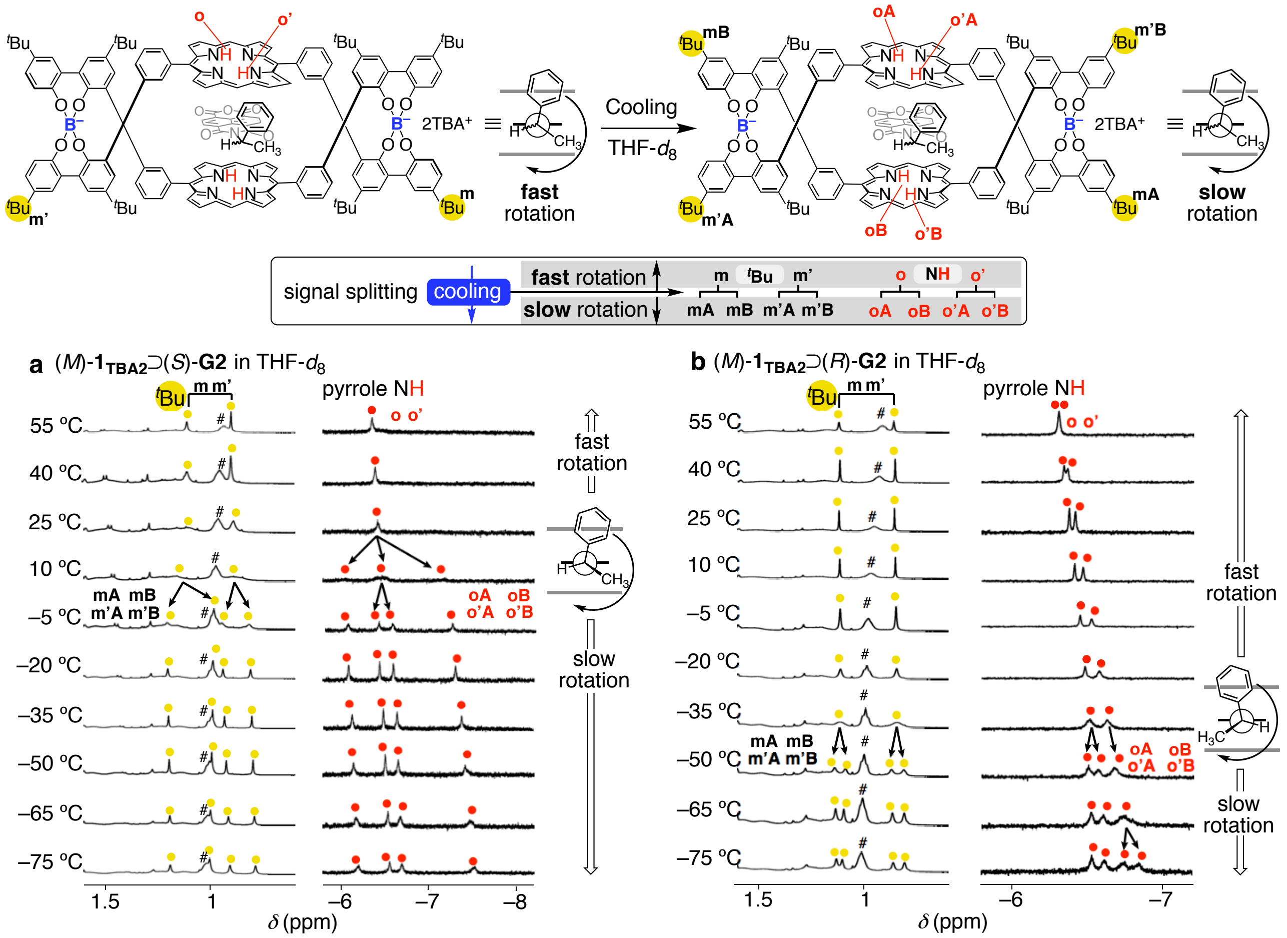

(**Supplementary Figure 21** to be continued)

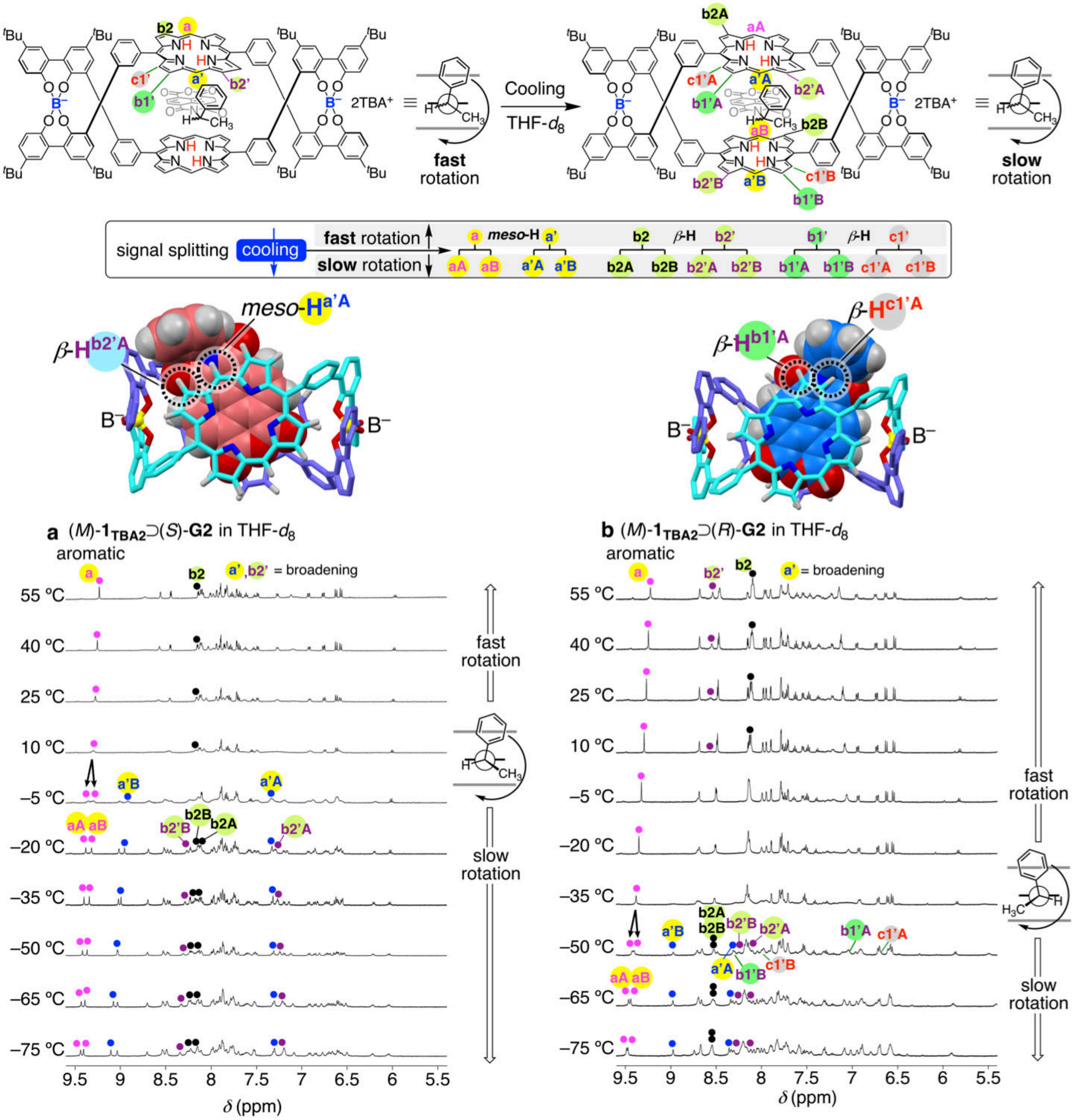

Supplementary Figure 21 | VT ^{1}H NMR spectra of diastereomeric inclusion complexes (*M*)-$\mathbf{1_{TBA2}}$⊃(*R*)-G2 and (*M*)-$\mathbf{1_{TBA2}}$⊃(*S*)-G2 in THF-*d*$_8$. VT ^{1}H NMR spectra (500 MHz, 0.40 mM) of (*M*)-$\mathbf{1_{TBA2}}$ in the presence of 1 equivalent of (*S*)-**G2** (**a**) and (*R*)-**G2** (**b**) in THF-*d*$_8$ from −75 to 55 °C. # denotes the protons from TBA. For peak assignments, see Supplementary Figs 24 and 25.

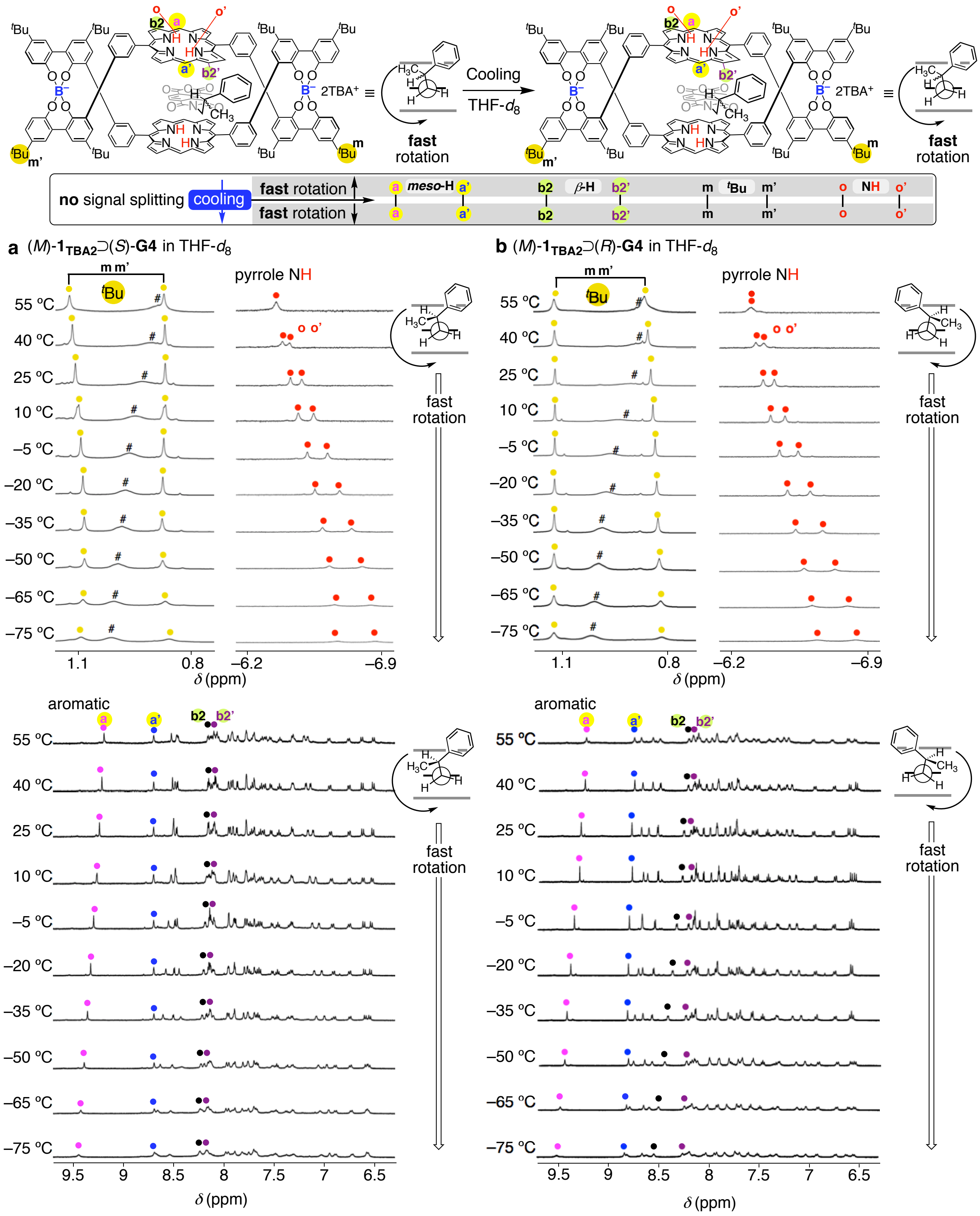

Supplementary Figure 22 | VT ^{1}H NMR spectra of diastereomeric inclusion complexes (*M*)-1$_{\mathrm{TBA2}}$⊃(*R*)-G4 and (*M*)-1$_{\mathrm{TBA2}}$⊃(*S*)-G4 in THF-*d*$_8$. VT ^{1}H NMR spectra (500 MHz, 0.40 mM) of (*M*)-**1**$_{\mathrm{TBA2}}$ in the presence of 1 equivalent of (*S*)-**G4** (**a**) and (*R*)-**G4** (**b**) in THF-*d*$_8$ from −75 to 55 °C. # denotes the protons from TBA. For peak assignments, see Supplementary Figs 23 and 40–43.

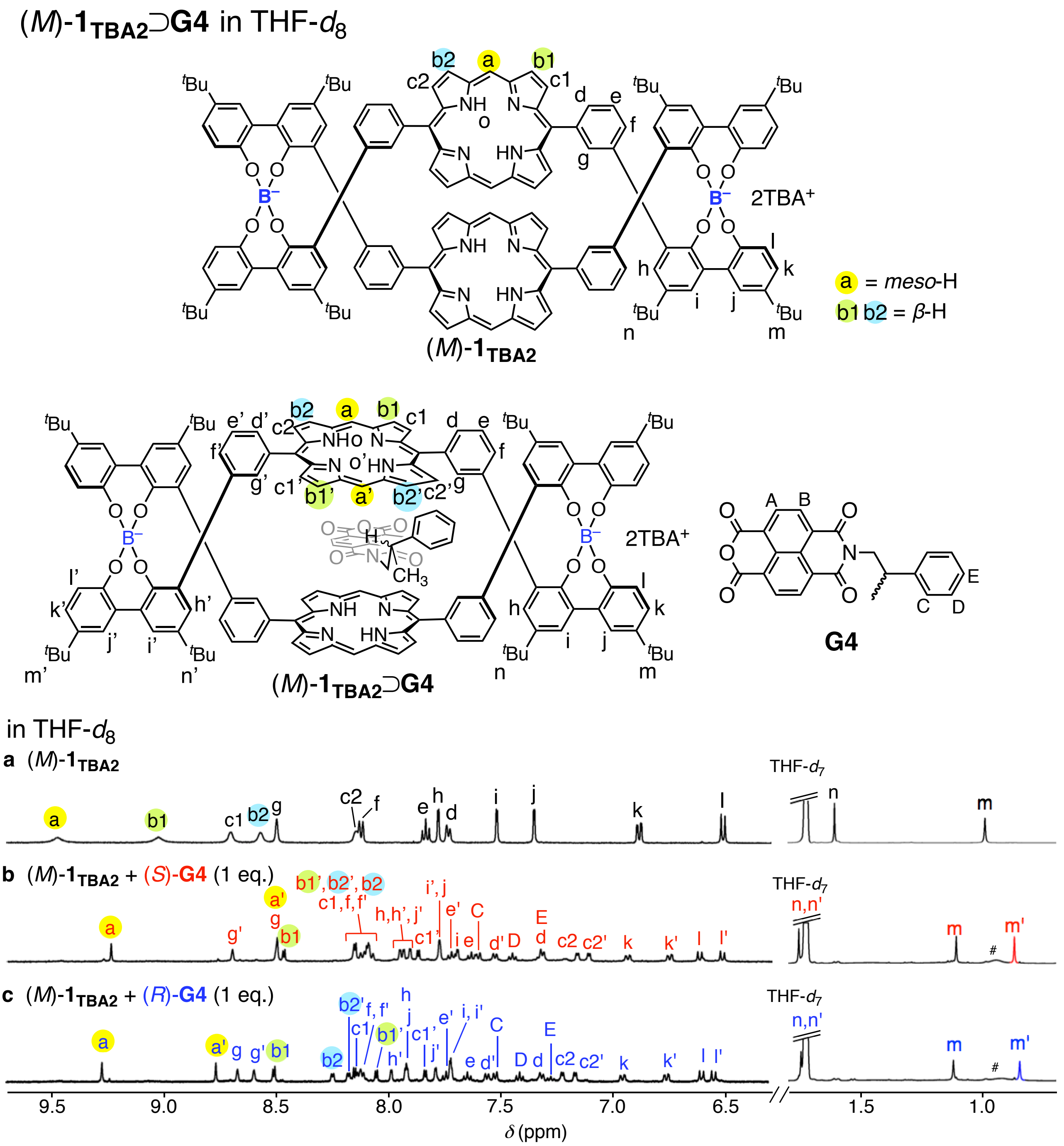

Supplementary Figure 23 | Diastereoselective inclusion complexation between (*M*)-$\mathbf{1}_{\mathbf{TBA2}}$ and *rac*-G4. Partial ^{1}H NMR spectra (500 MHz, 0.40 mM, ambient temperature) of (*M*)-$\mathbf{1}_{\mathbf{TBA2}}$ in the absence (**a**) and presence of 1 equivalent of (*S*)-**G4** (**b**) and (*R*)-**G4** (**c**) in THF-d_8. # denotes the protons from TBA. The signals of (*M*)-$\mathbf{1}_{\mathbf{TBA2}}$⊃(*R*)-**G4** and (*M*)-$\mathbf{1}_{\mathbf{TBA2}}$⊃(*S*)-**G4** were assigned by 2D gCOSY and ROESY spectroscopies (Supplementary Figs 40–43). The ^{1}H NMR spectrum of (*M*)-$\mathbf{1}_{\mathbf{TBA2}}$ in THF-d_8 is identical to that shown in Fig. 3b(i).

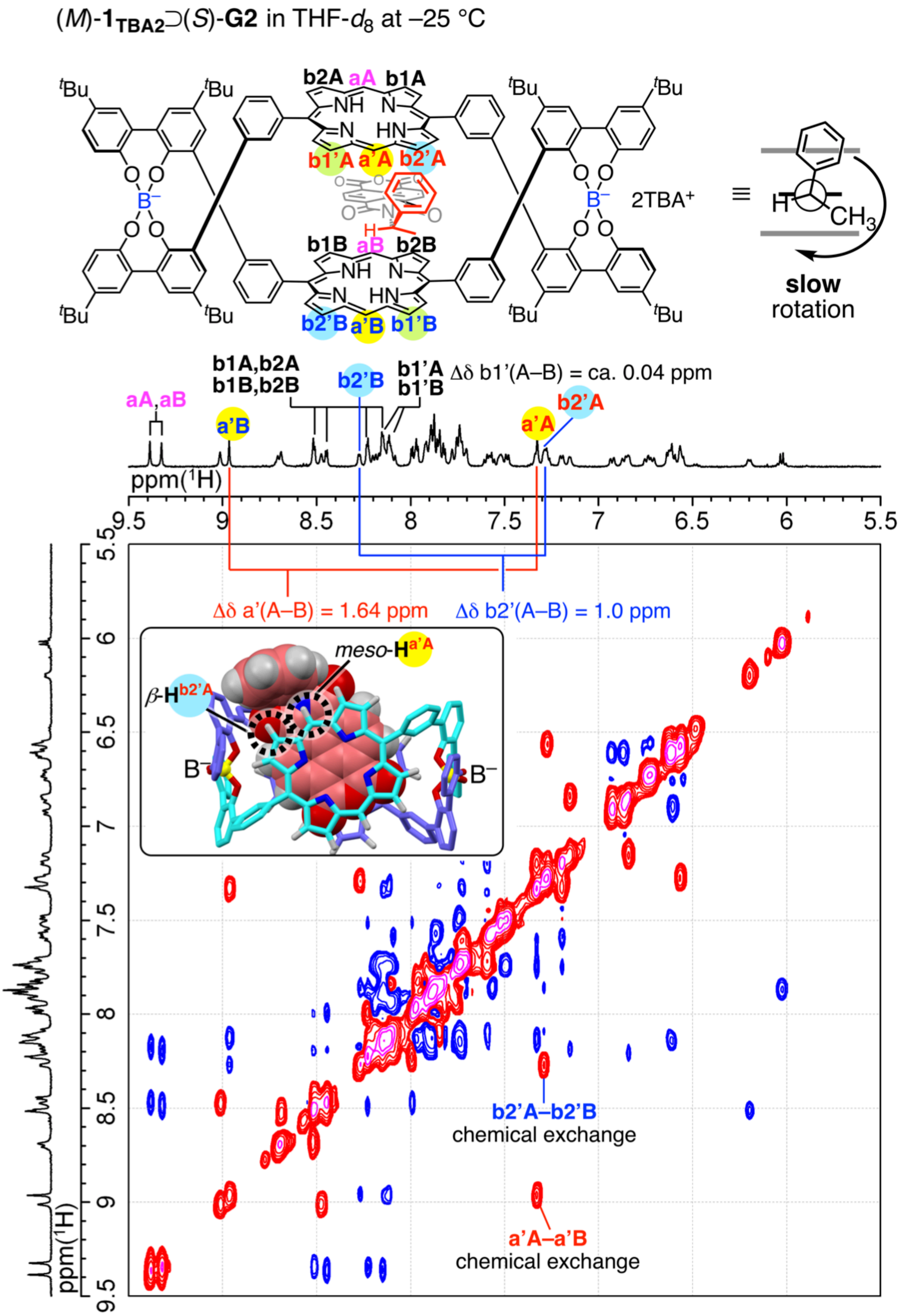

Supplementary Figure 24 | ROESY spectrum of (*M*)-1_{TBA2}⊃(*S*)-G2 in THF-*d*$_8$. Partial ROESY spectrum (500 MHz, THF-*d*$_8$, –25 ºC, mixing time = 200 ms) of (*M*)-$\mathbf{1}_{TBA2}$⊃(*S*)-**G2** (0.40 mM). The DFT calculated structure of (*M*)-$\mathbf{1}^{2-}$⊃(*S*)-**G2** is also shown.

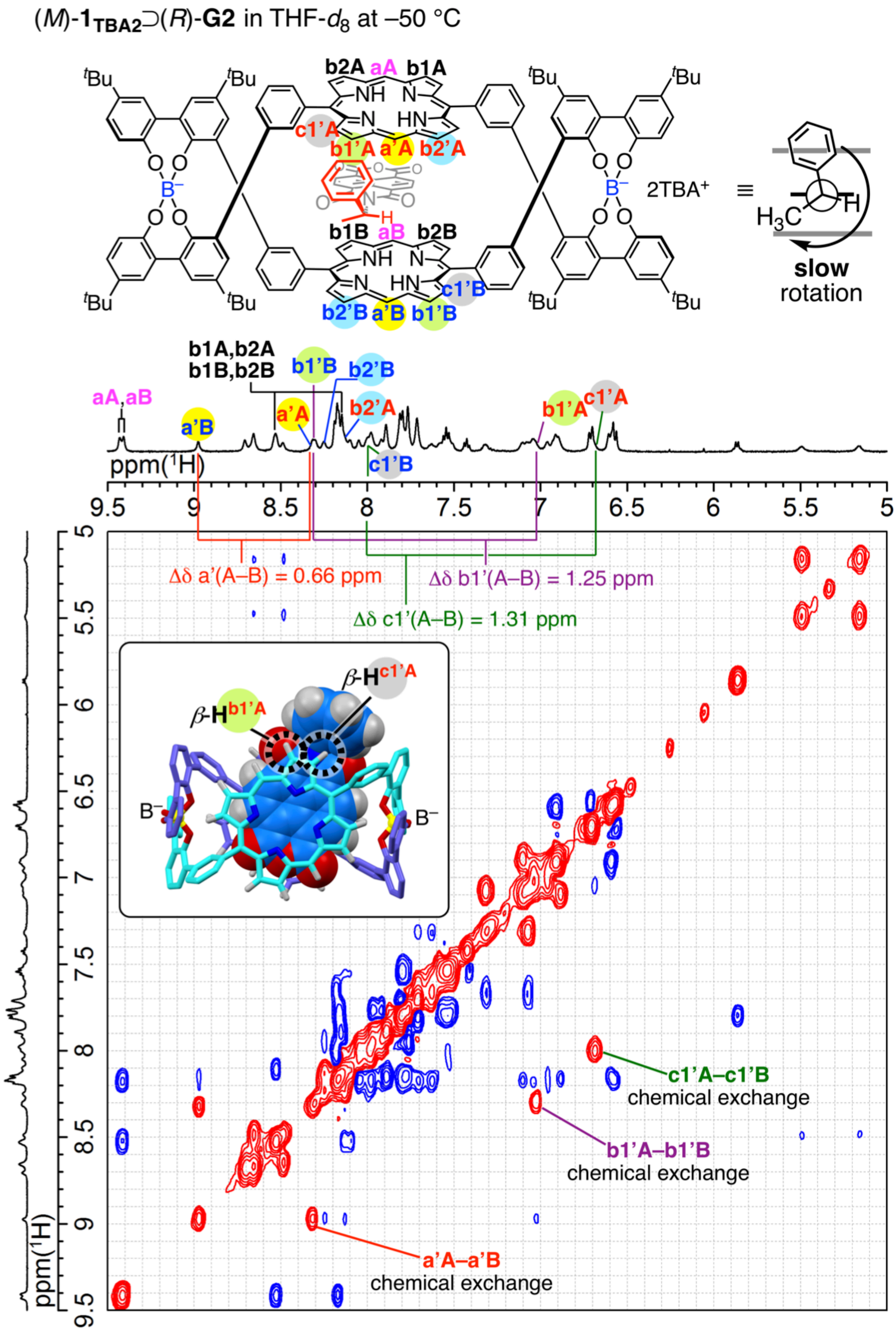

Supplementary Figure 25 | ROESY spectrum of (*M*)-1_{TBA2}⊃(*R*)-G2 in THF-*d*$_8$. Partial ROESY spectrum (500 MHz, THF-*d*$_8$, –50 °C, mixing time = 200 ms) of (*M*)-$\mathbf{1}_{TBA2}$⊃(*R*)-**G2** (0.40 mM). The DFT calculated structure of (*M*)-$\mathbf{1}^{2-}$⊃(*R*)-**G2** is also shown.

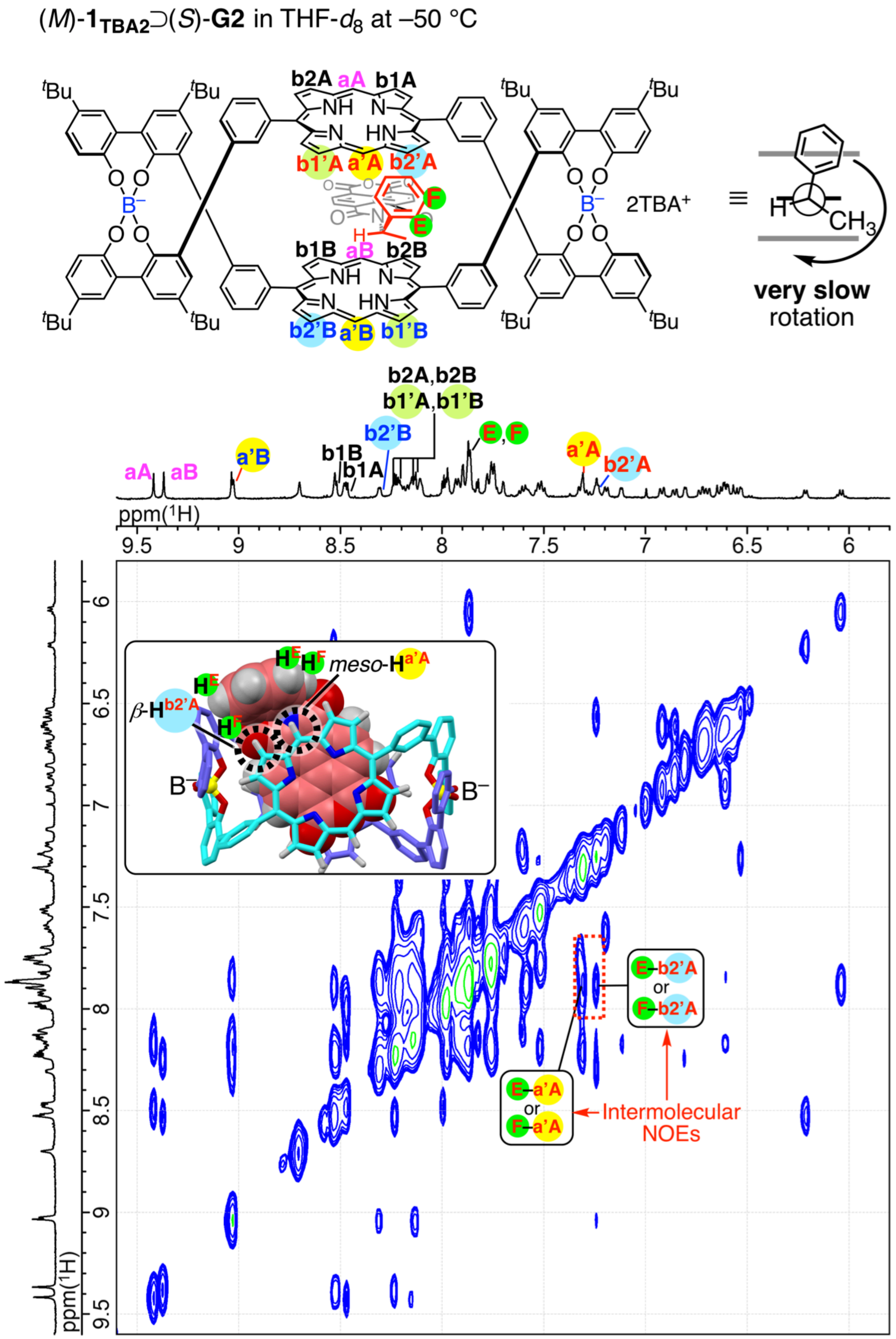

Supplementary Figure 26 | NOESY spectrum of (*M*)-$\mathbf{1_{TBA2}}$⊃(*S*)-G2 in THF-*d*$_8$. Partial NOESY spectrum (500 MHz, THF-*d*$_8$, –50 ºC, mixing time = 800 ms) of (*M*)-$\mathbf{1_{TBA2}}$⊃(*S*)-**G2** (0.40 mM). The DFT calculated structure of (*M*)-$\mathbf{1}^{2-}$⊃(*S*)-**G2** is also shown.

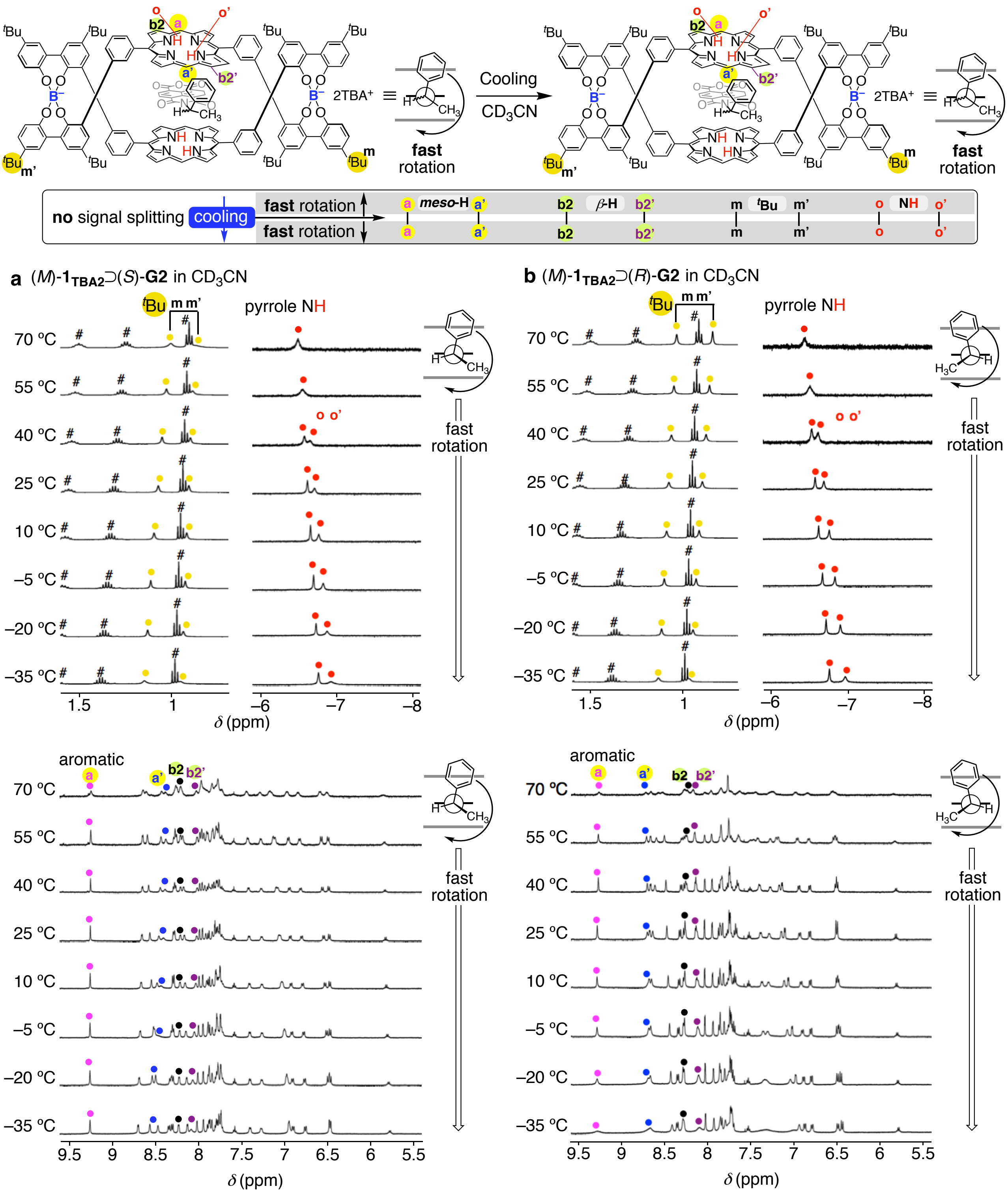

Supplementary Figure 27 | VT ^{1}H NMR spectra of diastereomeric inclusion complexes (*M*)-1$_{TBA2}$⊃(*S*)-G2 and (*M*)-1$_{TBA2}$⊃(*R*)-G2 in CD_3CN. VT ^{1}H NMR spectra (500 MHz, 0.40 mM) of (*M*)-**1**$_{TBA2}$ in the presence of 1 equivalent of (*S*)-**G2** (**a**) and (*R*)-**G2** (**b**) in CD_3CN from −35 to 70 °C. # denotes the protons from TBA. For peak assignments, see Supplementary Figs 28 and 36–39.

(M)-1_{TBA2}⊃G1 and (M)-1_{TBA2}⊃G2 in CD_3CN

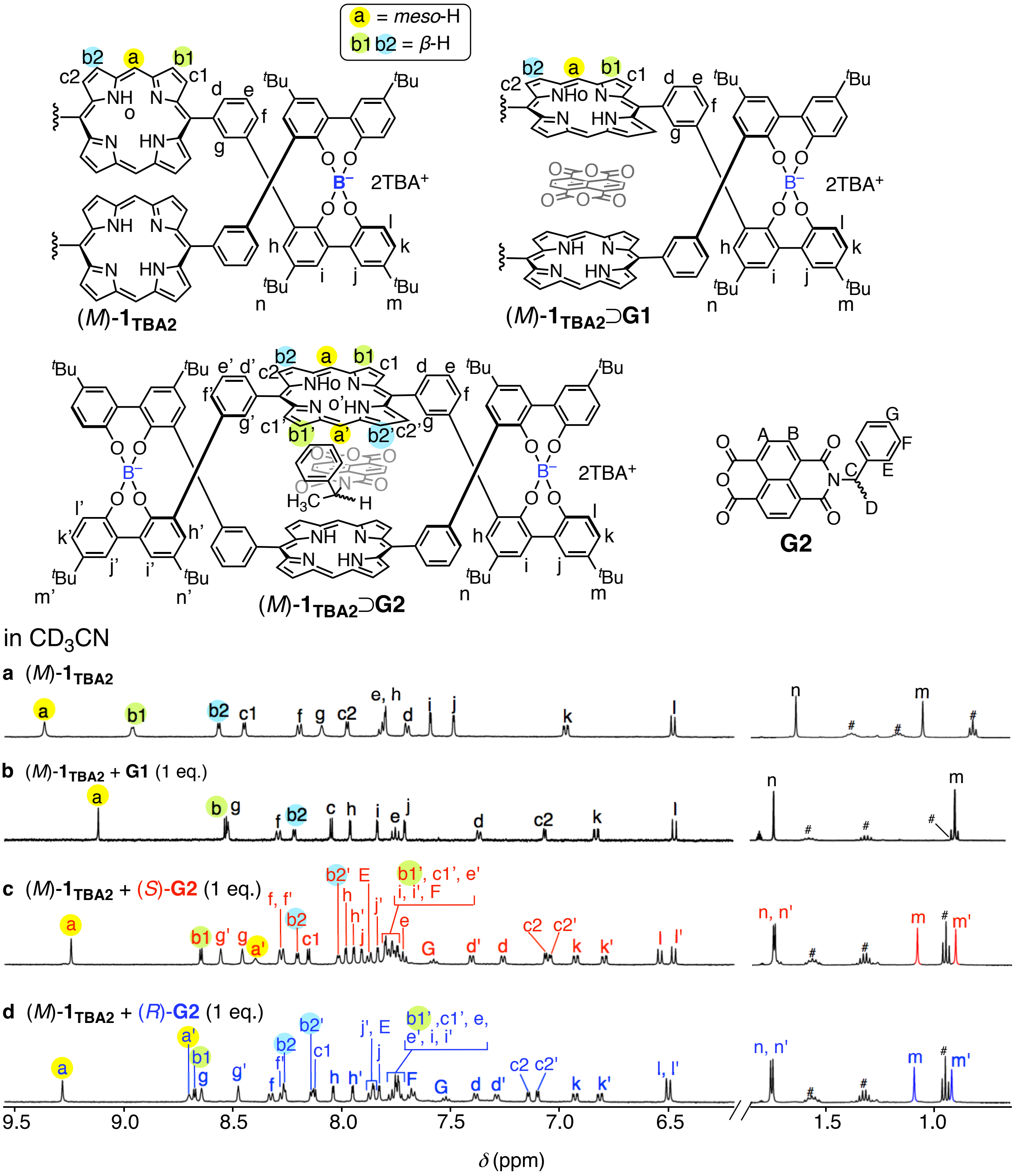

Supplementary Figure 28 | Diastereoselective inclusion complexation between (*M*)-1$_{TBA2}$ **and *rac*-G2.** Partial ^{1}H NMR spectra (500 MHz, 0.40 mM, ambient temperature) of (M)-**1**$_{TBA2}$ in the absence (**a**) and presence of 1 equivalent of **G1** (**b**), (S)-**G2** (**c**) and (R)-**G2** (**d**) in CD_3CN. # denotes the protons from TBA. The signal assignment of (M)-**1**$_{TBA2}$ in CD_3CN was previously reported.[1] The signals of (M)-**1**$_{TBA2}$⊃(S)-**G2** and (M)-**1**$_{TBA2}$⊃(R)-**G2** were assigned by 2D gCOSY and ROESY spectroscopies (Supplementary Figs 36–39).

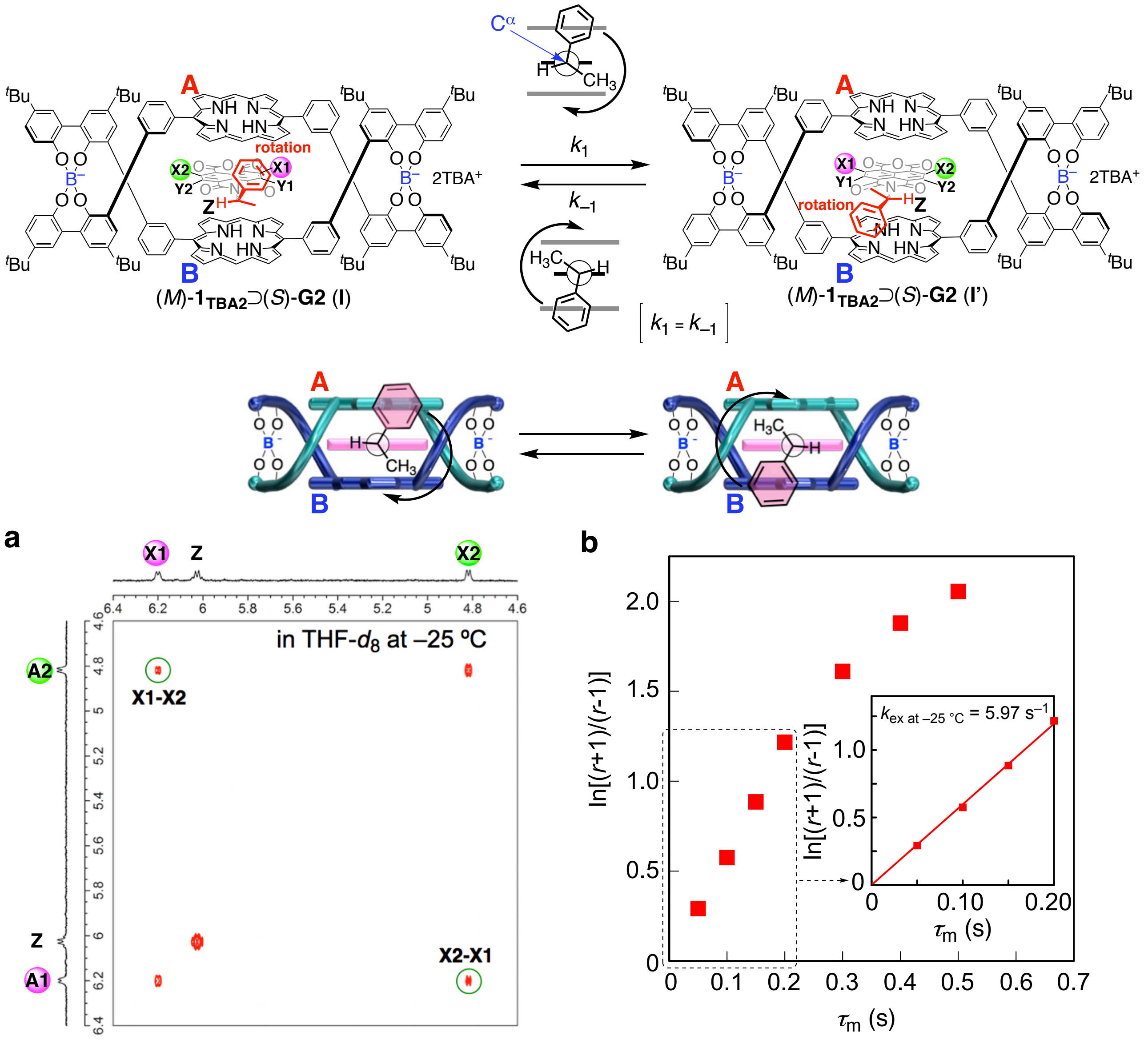

Supplementary Figure 29 | Determination of the apparent exchange rate constant, k_{ex} ($k_{ex} = k_1 + k_{-1}$), between two rotational conformers resulting from slow rotation of the (*S*)-phenylethyl group around the N-C$^{\alpha}$ bond of (*S*)-**G2** sandwiched between the bisporphyrin of (*M*)-$\mathbf{1}_{\mathbf{TBA2}}$ at –25 °C. (**a**) Partial 2D EXSY (NOESY) spectrum (500 MHz, THF-d_8, –25 °C, 0.4 mM, mixing time (τ_m) = 0.2 s) of (*M*)-$\mathbf{1}_{\mathbf{TBA2}}$⊃(*S*)-**G2**. (**b**) Plots of ln[(r + 1)/(r – 1)] as a function of τ_m in THF-d_8 at –25 °C. Source data of (**b**) are provided as a Source Data file.

2D NMR Spectra of (*M*)-1_{TBA2} and Its Inclusion Complexes with (*R*)-G2, (*S*)-G2, (*R*)-G4, and (*S*)-G4.

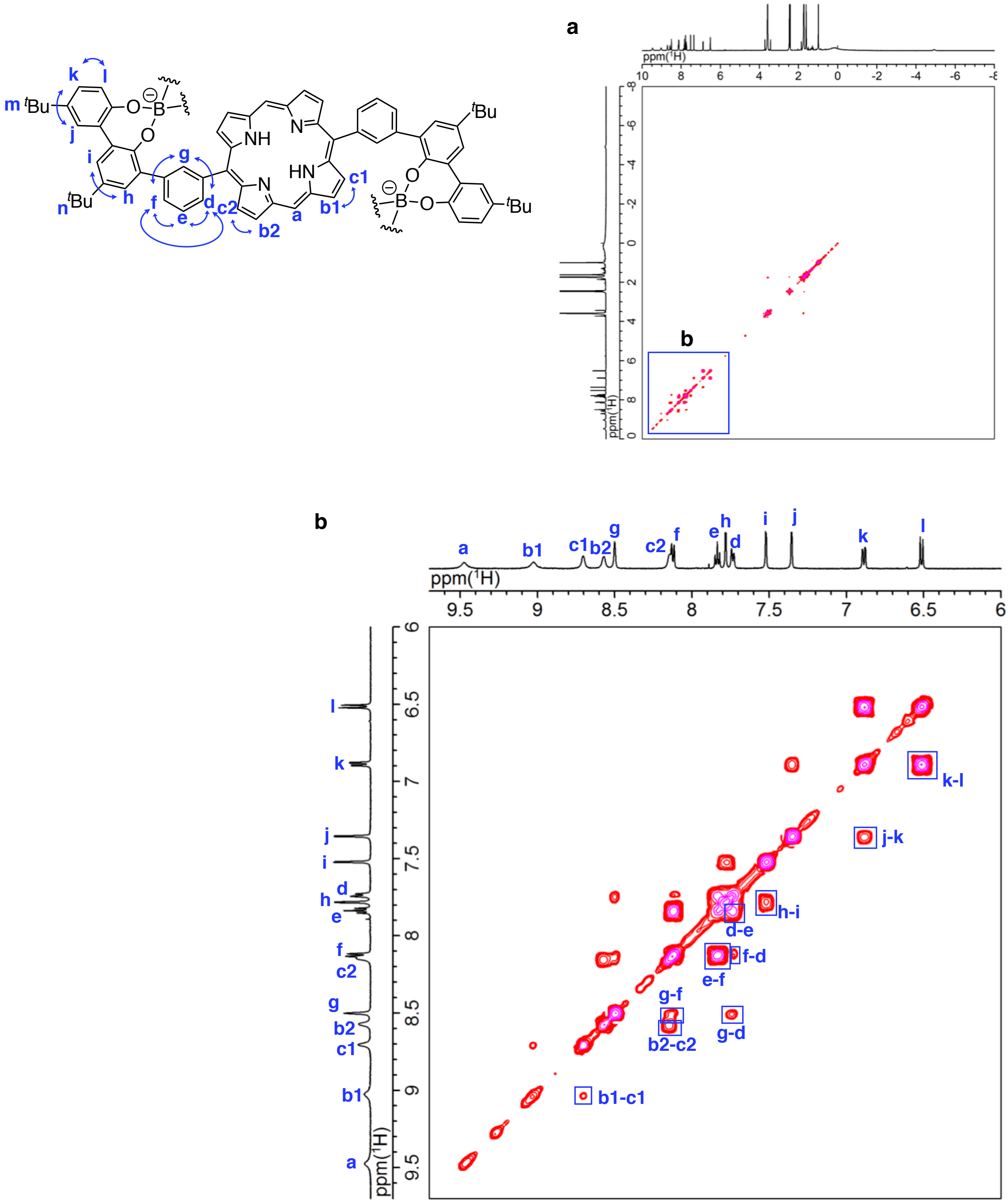

Supplementary Figure 30 | gCOSY spectra of (*M*)-1_{TBA2} in THF-d_8. Full (**a**) and partial (**b**) gCOSY spectra (500 MHz, THF-d_8, 25 ºC) of (*M*)-$\mathbf{1}_{TBA2}$ (0.40 mM).

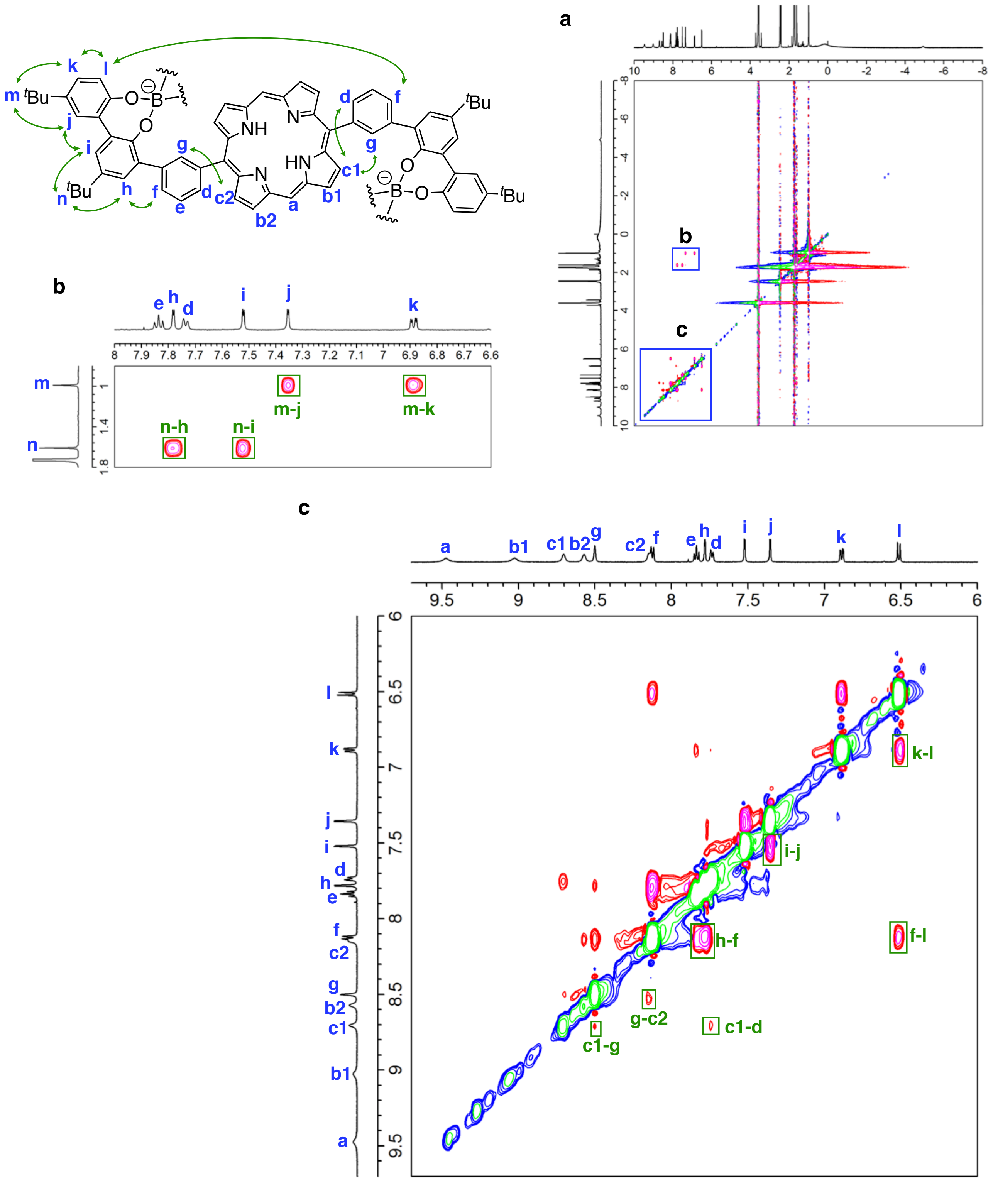

Supplementary Figure 31 | ROESY spectra of (*M*)-1_{TBA2} in THF-d_8. Full (**a**) and partial (**b** and **c**) ROESY spectra (500 MHz, THF-d_8, 25 ºC, mixing time = 200 ms) of (*M*)-$\mathbf{1}_{TBA2}$ (0.40 mM).

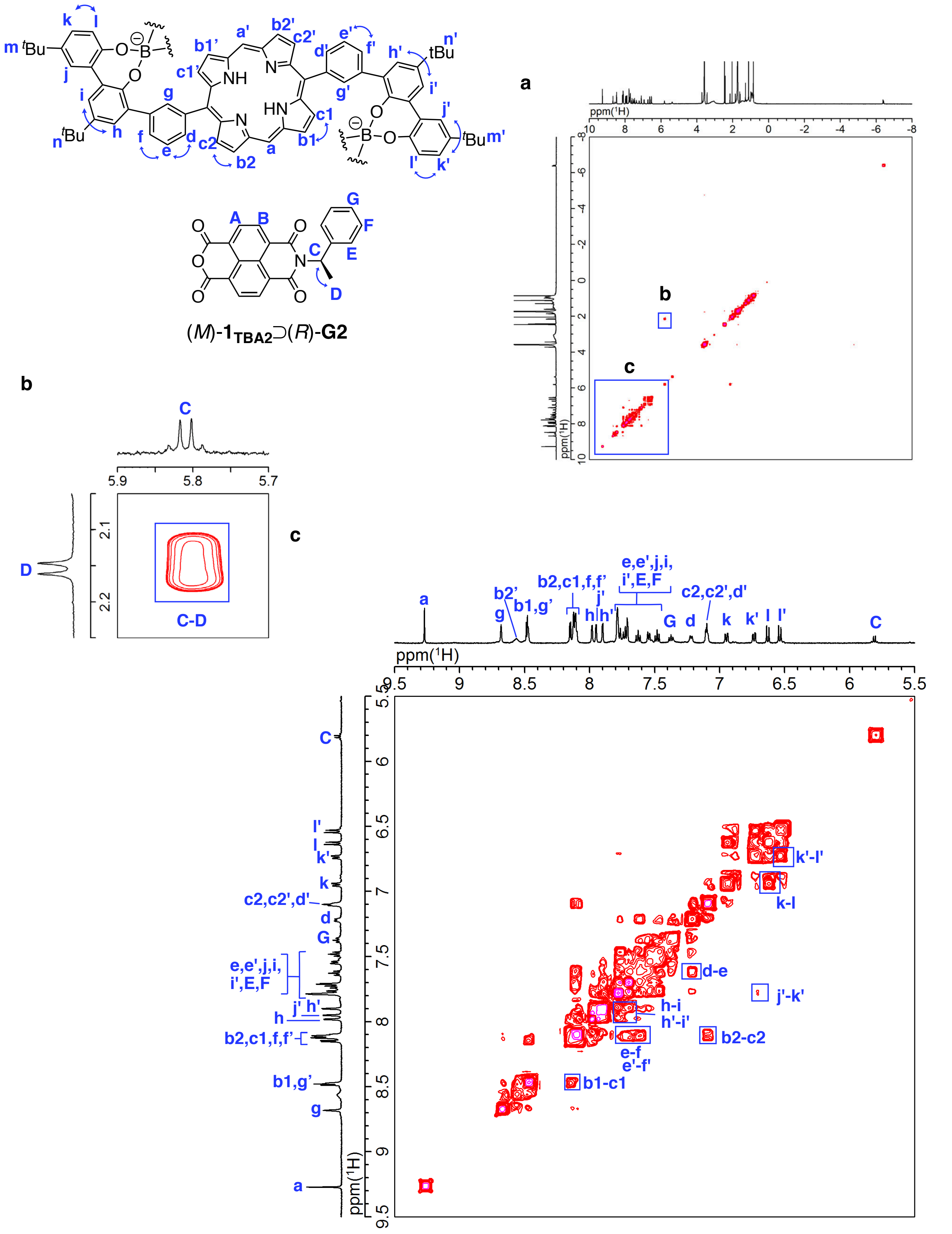

Supplementary Figure 32 | gCOSY spectra of (*M*)-$\mathbf{1}_{\mathbf{TBA2}}$⊃(*R*)-G2 in THF-*d*$_8$. Full (**a**) and partial (**b** and **c**) gCOSY spectra (500 MHz, THF-*d*$_8$, 25 ºC) of (*M*)-$\mathbf{1}_{\mathbf{TBA2}}$⊃(*R*)-**G2** (0.40 mM).

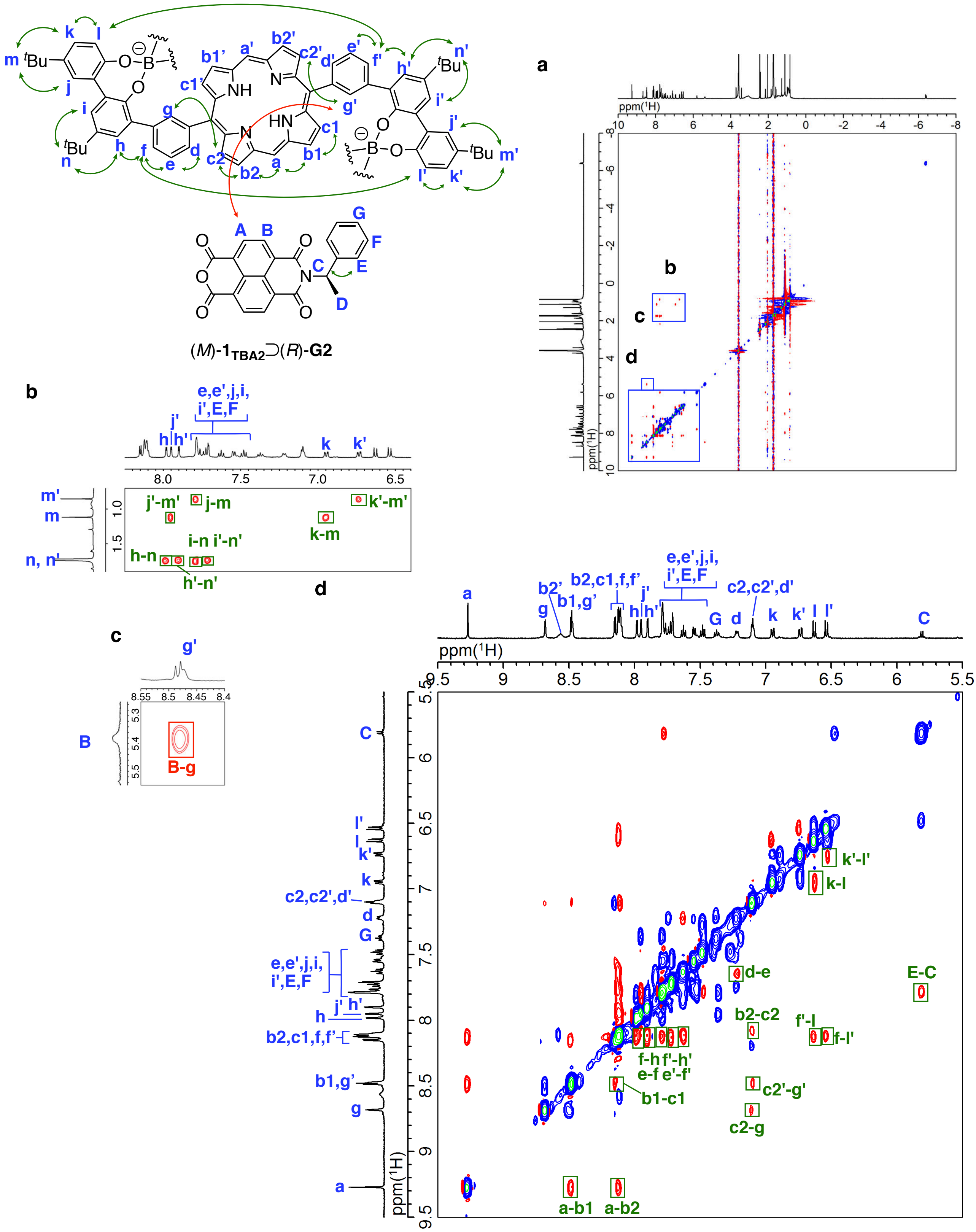

Supplementary Figure 33 | ROESY spectra of (*M*)-1$_{TBA2}$⊃(*R*)-G2 in THF-*d*$_8$. Full (**a**) and partial (**b**–**d**) ROESY spectra (500 MHz, THF-*d*$_8$, 25 ºC, mixing time = 200 ms) of (*M*)-**1**$_{TBA2}$⊃(*R*)-**G2** (0.40 mM). Intermolecular NOE cross-peaks are indicated by red arrows.

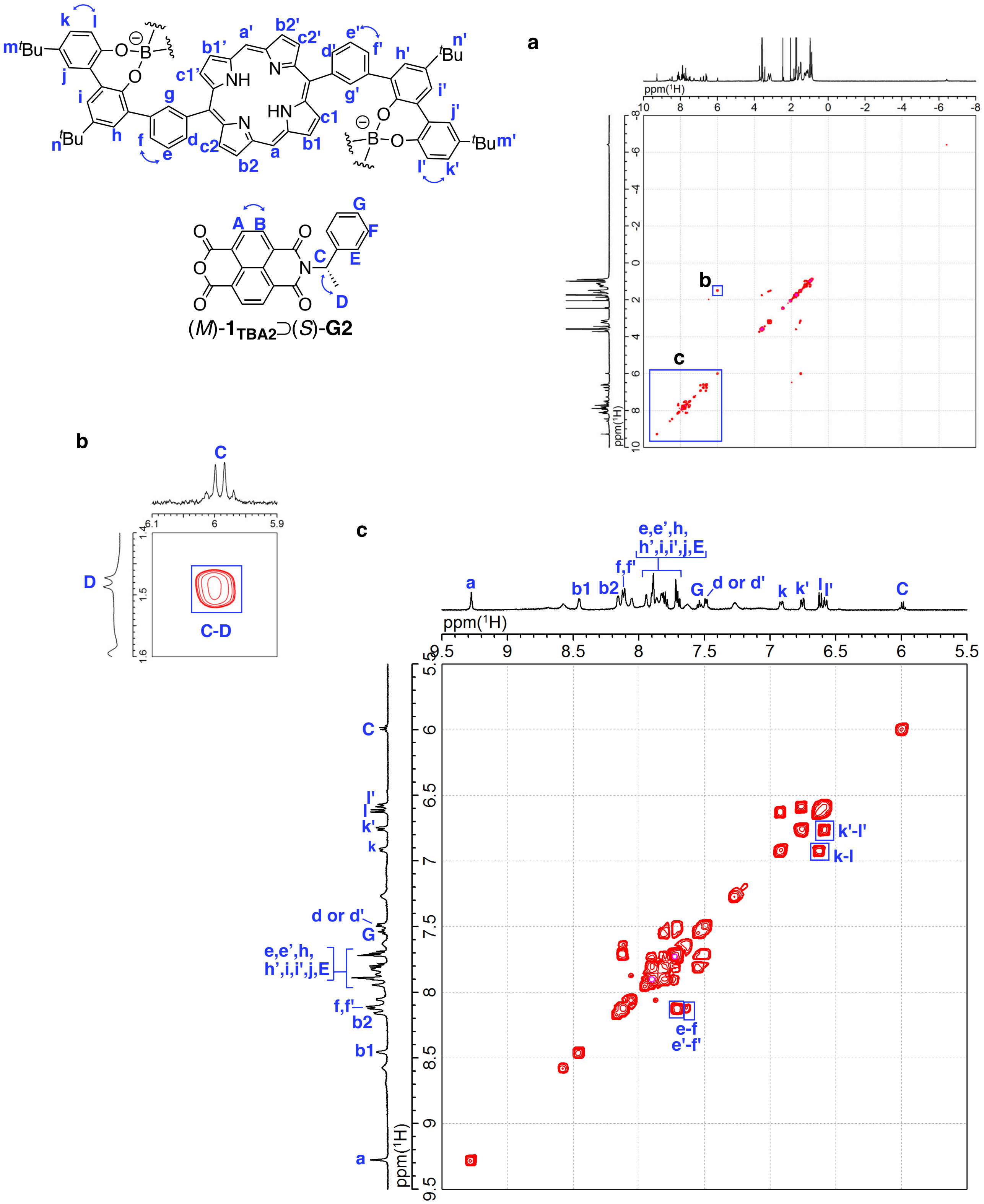

Supplementary Figure 34 | gCOSY spectra of (*M*)-1$_{TBA2}$⊃(*S*)-G2 in THF-*d*$_8$. Full (**a**) and partial (**b** and **c**) gCOSY spectra (500 MHz, THF-*d*$_8$, 25 ºC) of (*M*)-**1**$_{TBA2}$⊃(*S*)-**G2** (0.40 mM).

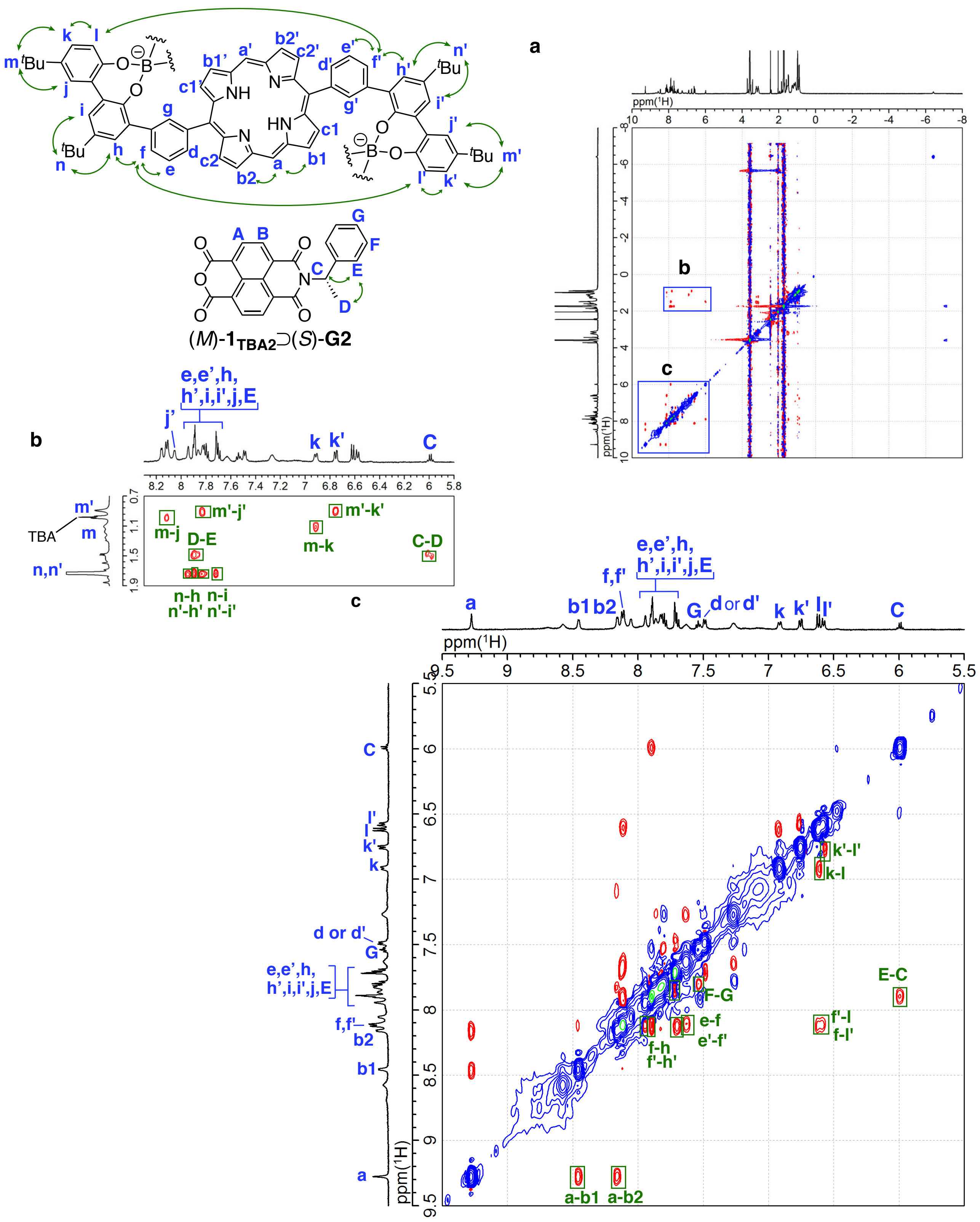

Supplementary Figure 35 | ROESY spectra of (*M*)-1$_{TBA2}$⊃(*S*)-G2 in THF-*d*$_8$. Full (**a**) and partial (**b** and **c**) ROESY spectra (500 MHz, THF-*d*$_8$, 25 °C, mixing time = 200 ms) of (*M*)-**1**$_{TBA2}$⊃(*S*)-**G2** (0.40 mM). Intermolecular NOE cross-peaks are indicated by red arrows.

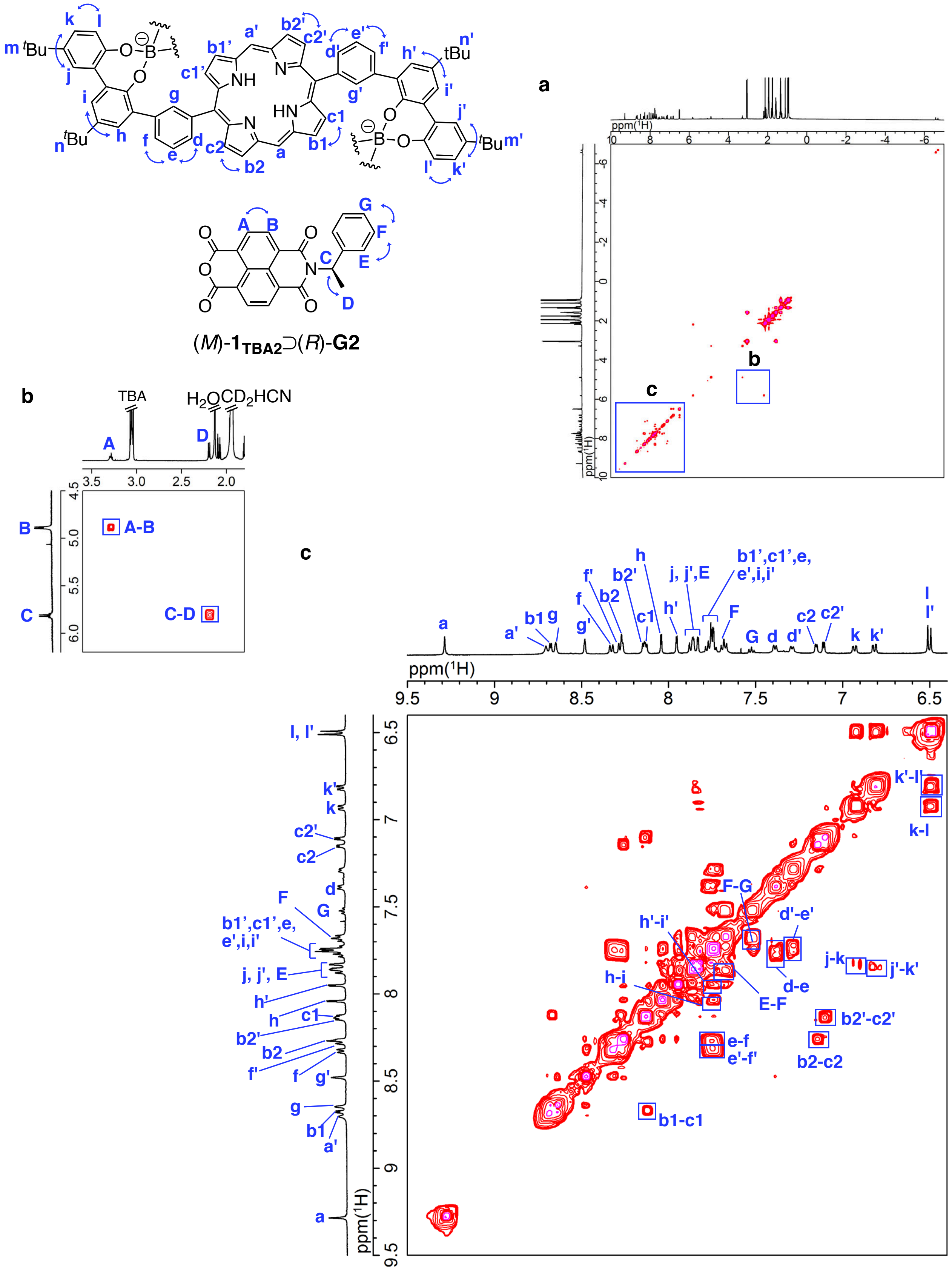

Supplementary Figure 36 | gCOSY spectra of (*M*)-1$_{TBA2}$⊃(*R*)-G2 in CD$_3$CN. Full (**a**) and partial (**b** and **c**) gCOSY spectra (500 MHz, CD$_3$CN, 25 ºC) of (*M*)-**1**$_{TBA2}$⊃(*R*)-**G2** (0.40 mM).

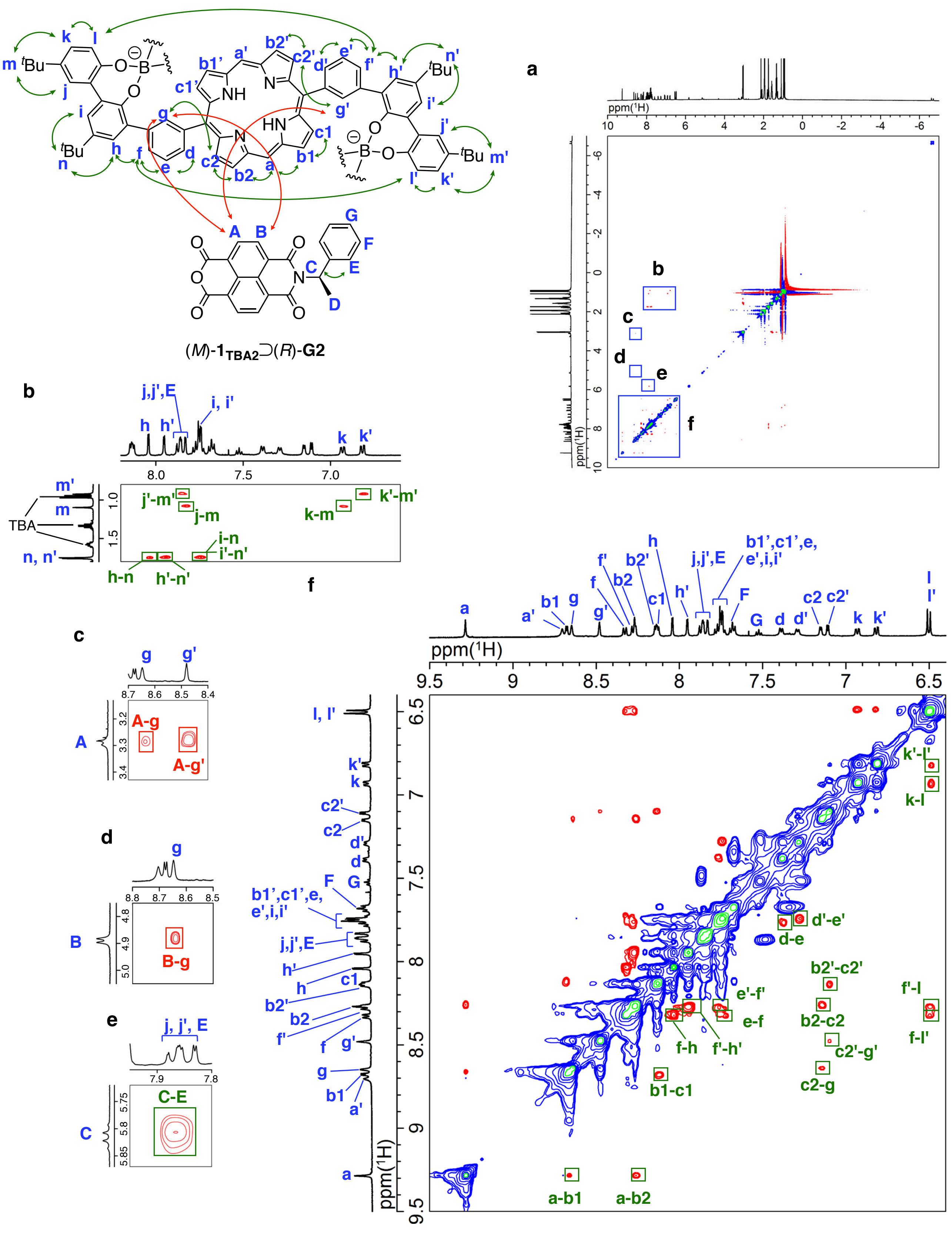

Supplementary Figure 37 | ROESY spectra of (*M*)-1$_{TBA2}$⊃(*R*)-G2 in CD_3CN. Full (**a**) and partial (**b**–**f**) ROESY spectra (500 MHz, CD_3CN, 25 ºC, mixing time = 200 ms) of (*M*)-**1**$_{TBA2}$⊃(*R*)-**G2** (0.40 mM). Intermolecular NOE cross-peaks are indicated by red arrows.

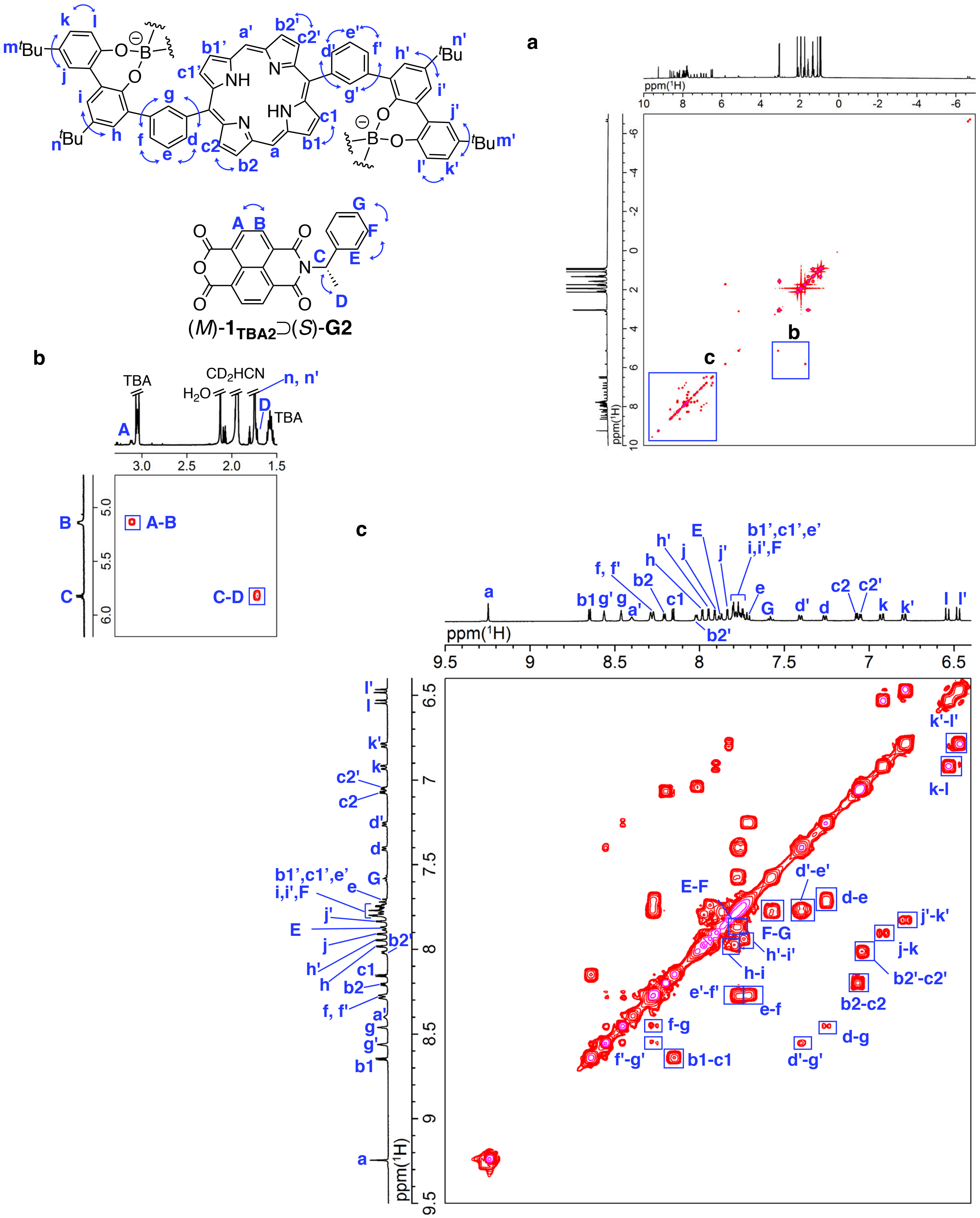

Supplementary Figure 38| gCOSY spectra of (*M*)-1$_{TBA2}$⊃(*S*)-G2 in CD$_3$CN. Full (**a**) and partial (**b** and **c**) gCOSY spectra (500 MHz, CD$_3$CN, 25 ºC) of (*M*)-**1**$_{TBA2}$⊃(*S*)-**G2** (0.40 mM).

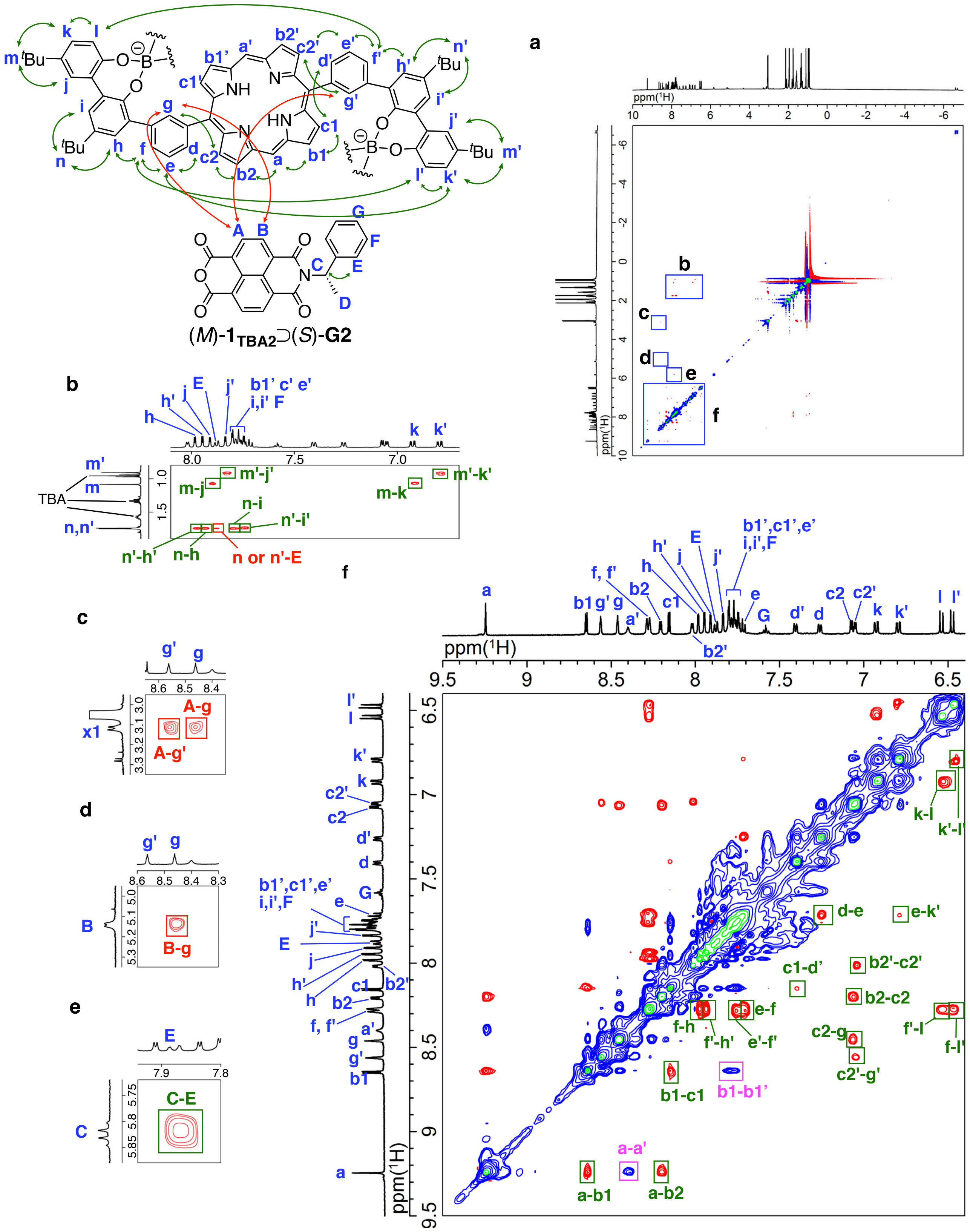

Supplementary Figure 39| ROESY spectra of (*M*)-$\mathbf{1_{TBA2}}$⊃(*S*)-G2 in CD_3CN. Full (**a**) and partial (**b**–**f**) ROESY spectra (500 MHz, CD_3CN, 25 ºC, mixing time = 200 ms) of (*M*)-$\mathbf{1_{TBA2}}$⊃(*S*)-**G2** (0.40 mM). Intermolecular NOE cross-peaks are indicated by red arrows.

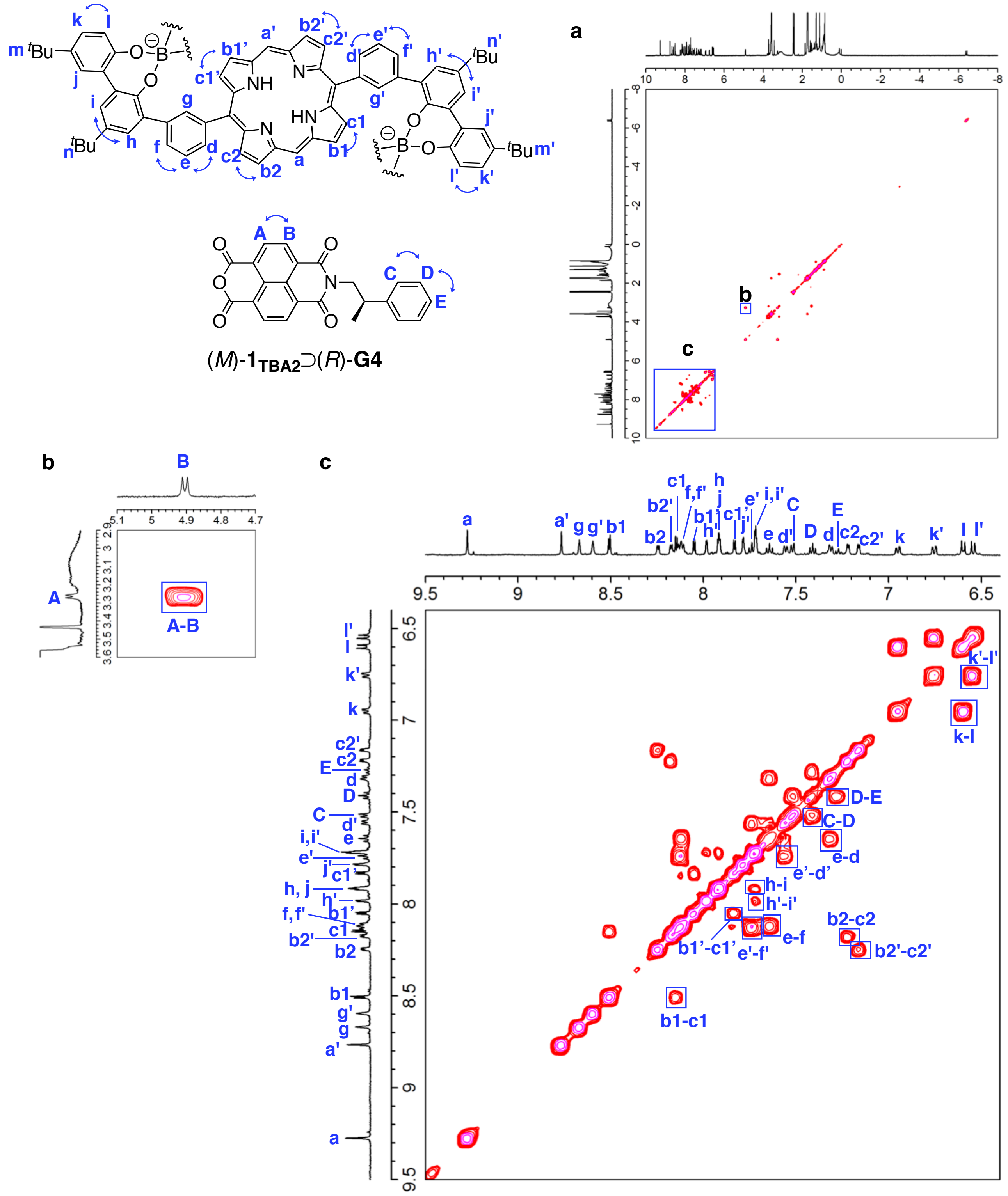

Supplementary Figure 40 | gCOSY spectra of (*M*)-$\mathbf{1_{TBA2}}$⊃(*R*)-G4 in THF-d_8. Full (**a**) and partial (**b** and **c**) gCOSY spectra (500 MHz, THF-d_8, 25 ºC) of (*M*)-$\mathbf{1_{TBA2}}$⊃(*R*)-**G4** (0.40 mM).

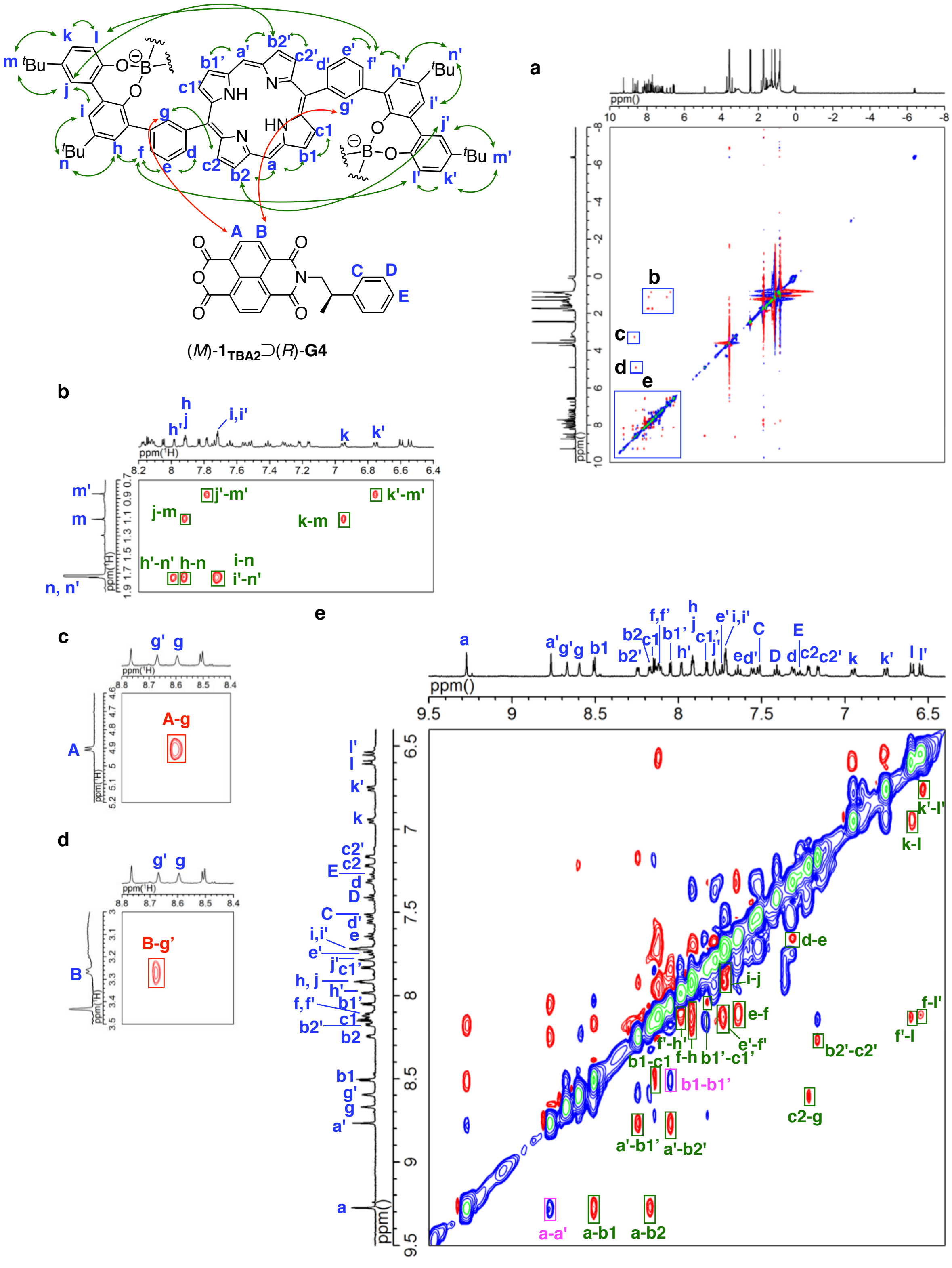

Supplementary Figure 41 | ROESY spectra of (*M*)-1$_{TBA2}$⊃(*R*)-G4 in THF-*d*$_8$. Full (**a**) and partial (**b**–**e**) ROESY spectra (500 MHz, THF-*d*$_8$, 25 ºC, mixing time = 200 ms) of (*M*)-**1**$_{TBA2}$⊃(*R*)-**G4** (0.40 mM). Intermolecular NOE cross-peaks are indicated by red arrows

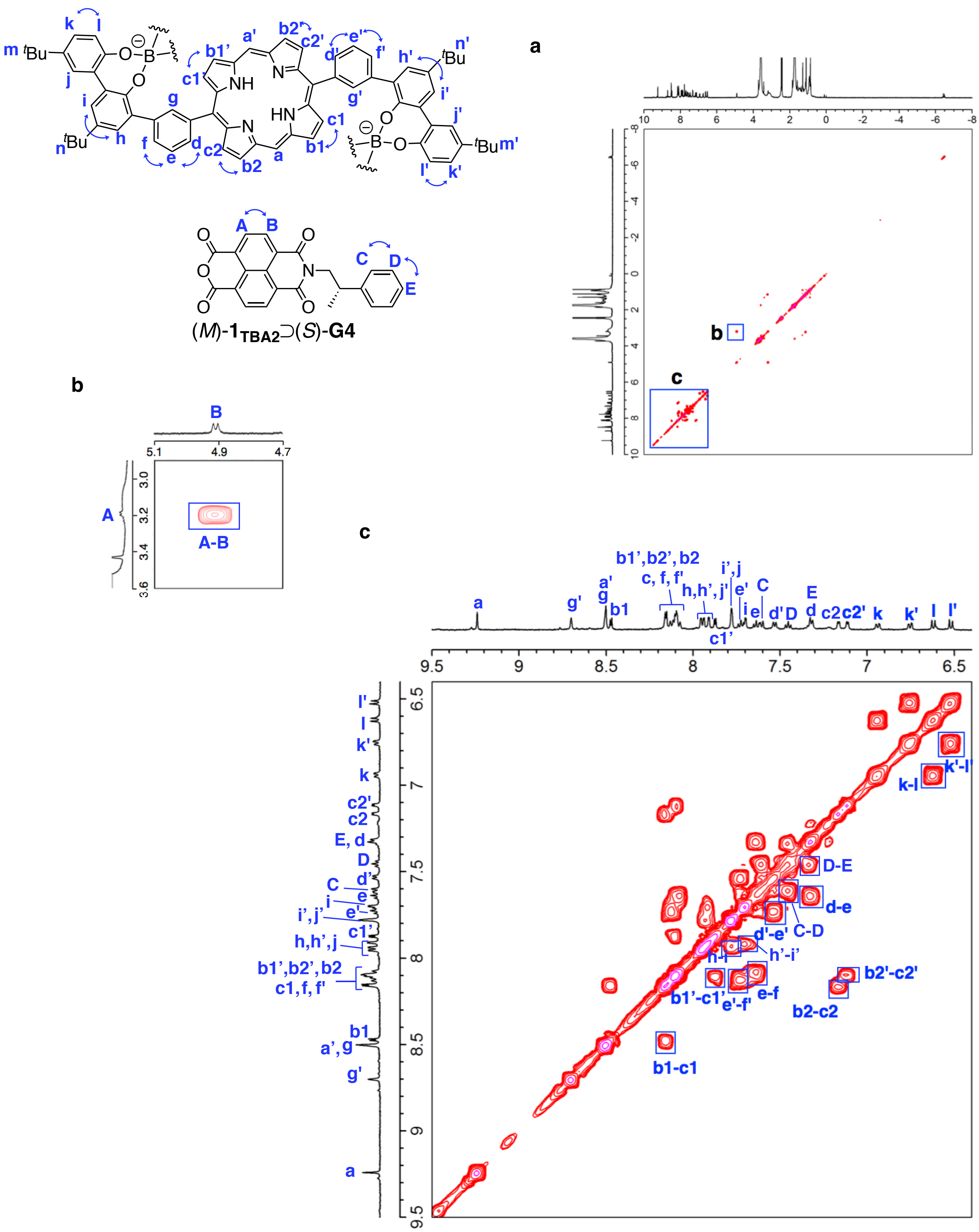

Supplementary Figure 42 | gCOSY spectra of (*M*)-1$_{TBA2}$⊃(*S*)-G4 in THF-*d*$_8$. Full (**a**) and partial (**b** and **c**) gCOSY spectra (500 MHz, THF-*d*$_8$, 25 ºC) of (*M*)-**1**$_{TBA2}$⊃(*S*)-**G4** (0.40 mM).

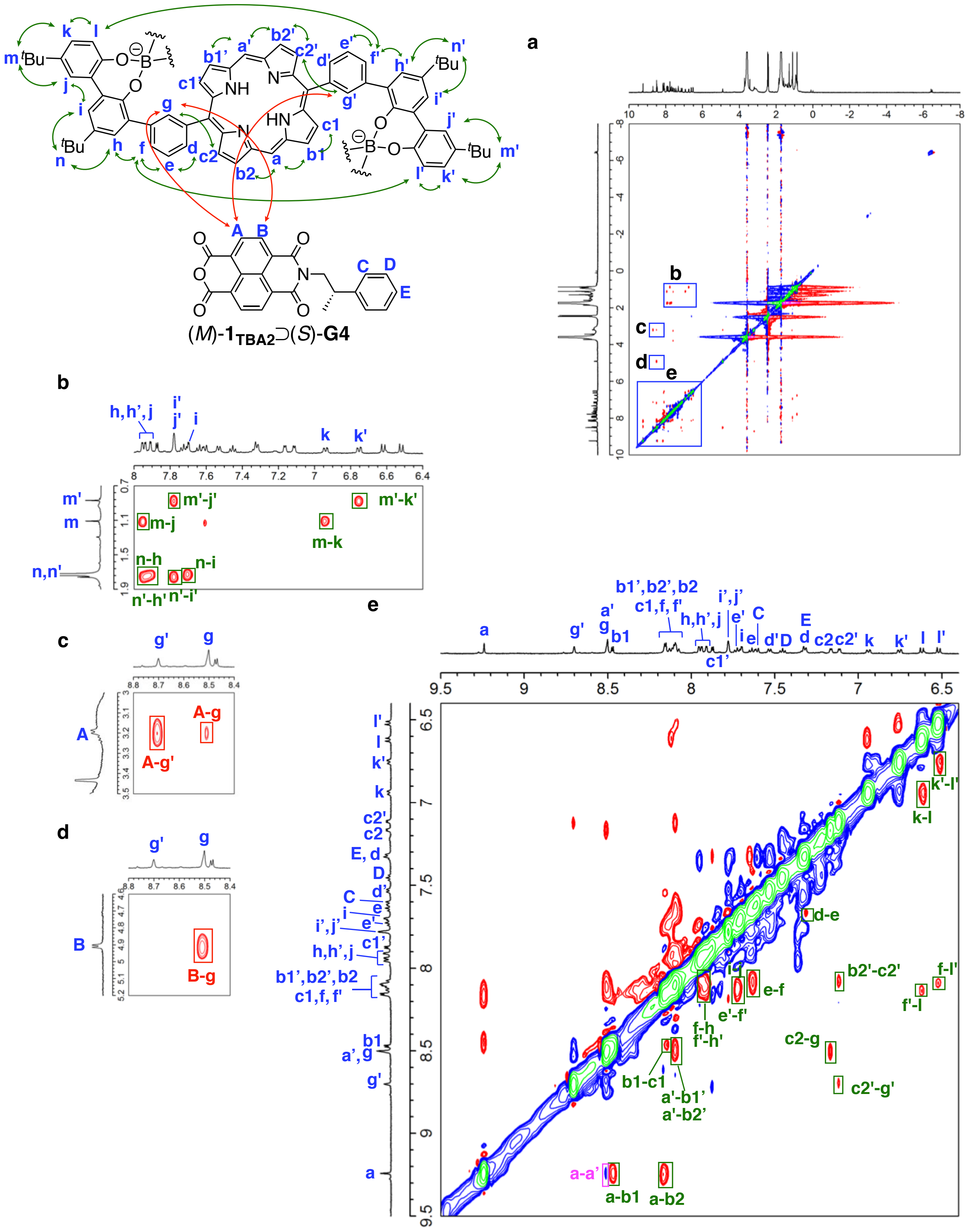

Supplementary Figure 43 | ROESY spectra of (*M*)-1$_{\mathbf{TBA2}}$⊃(*S*)-G4 in THF-*d*$_8$. Full (**a**) and partial (**b**–**e**) ROESY spectra (500 MHz, THF-*d*$_8$, 25 ºC, mixing time = 200 ms) of (*M*)-**1**$_{\mathbf{TBA2}}$⊃(*S*)-**G4** (0.40 mM). Intermolecular NOE cross-peaks are indicated by red arrows.

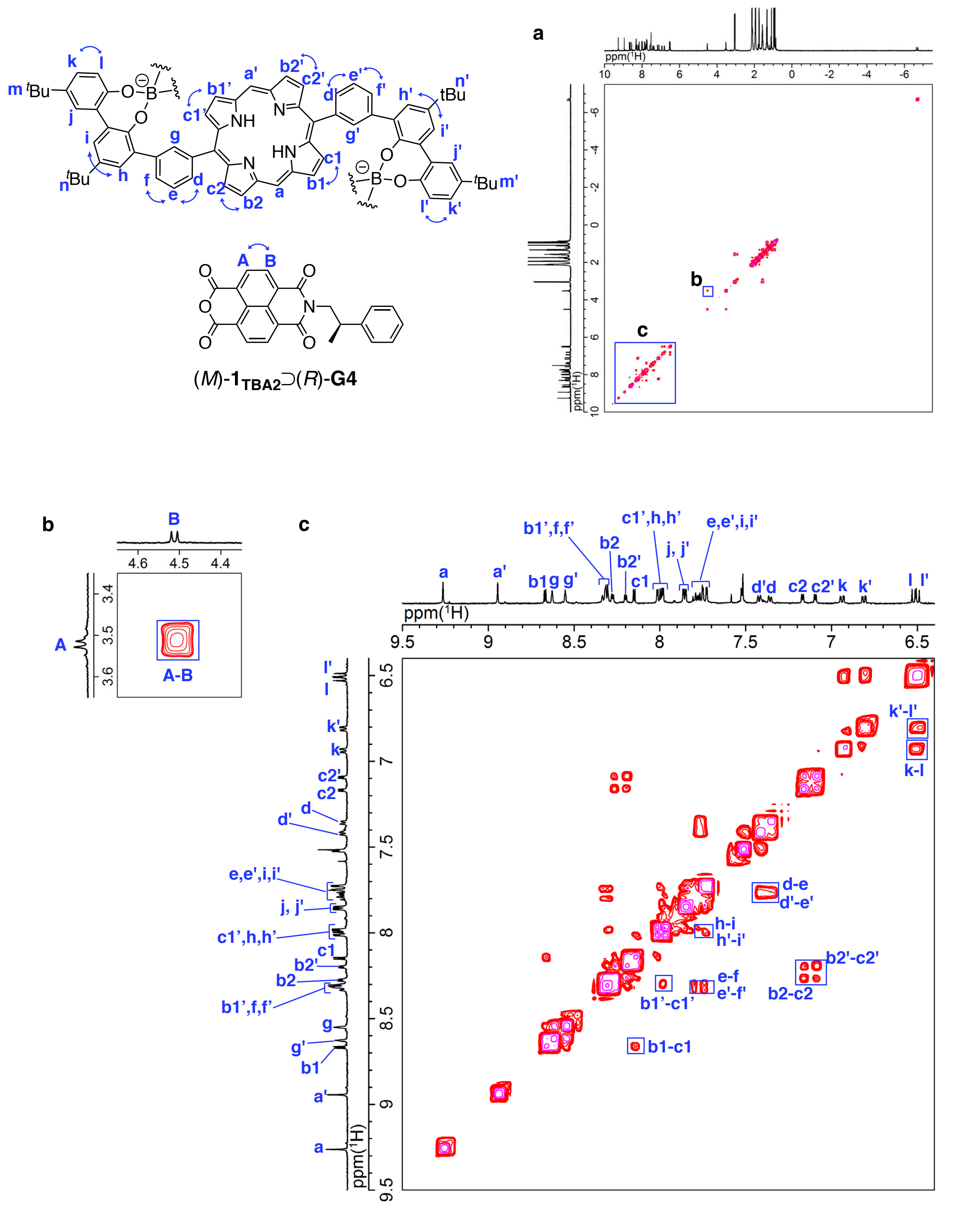

Supplementary Figure 44 | gCOSY spectra of (*M*)-1$_{TBA2}$⊃(*R*)-G4 in CD$_3$CN. Full (**a**) and partial (**b** and **c**) gCOSY spectra (500 MHz, CD$_3$CN, 25 ºC) of (*M*)-**1**$_{TBA2}$⊃(*R*)-**G4** (0.40 mM).

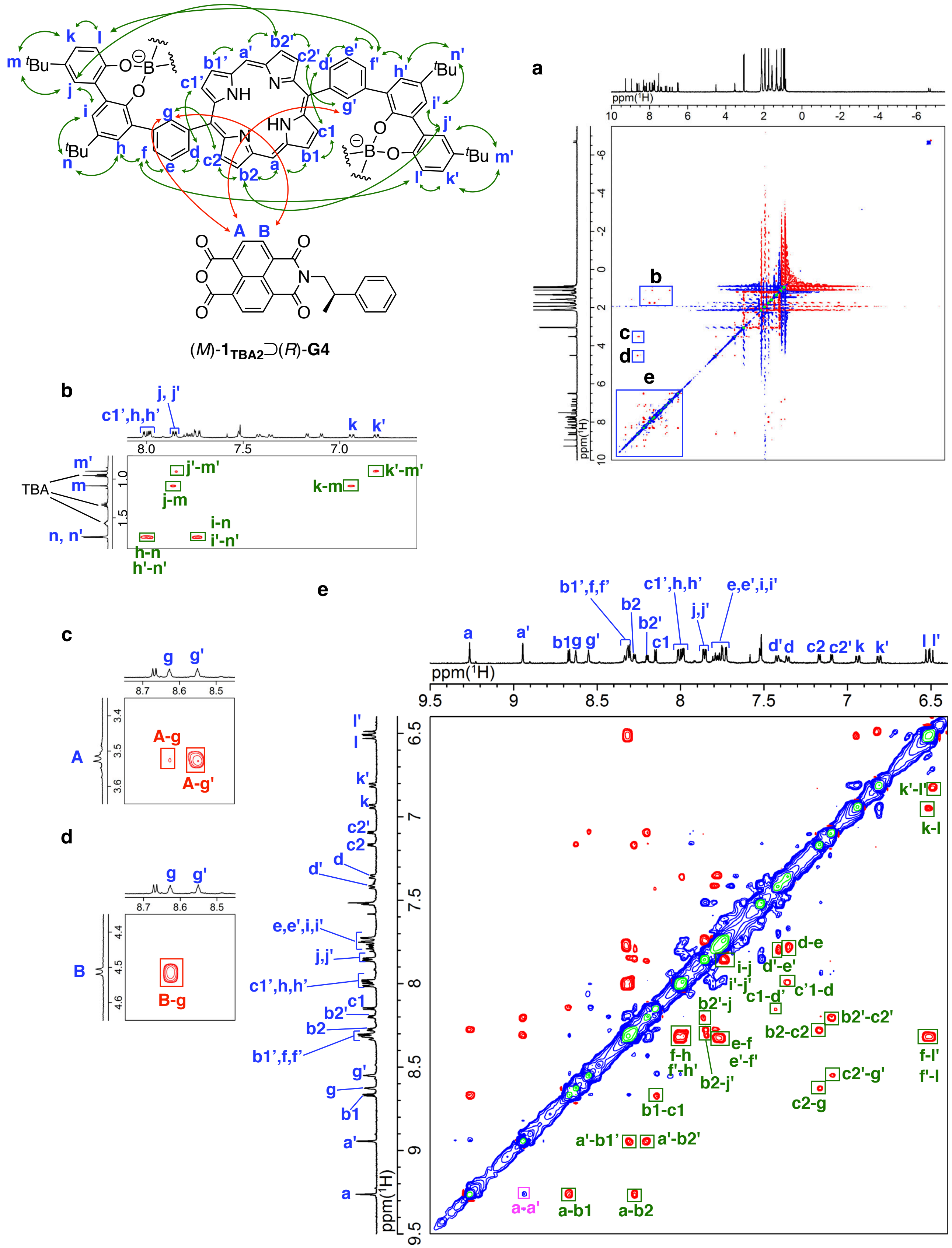

Supplementary Figure 45 | ROESY spectra of (*M*)-1$_{TBA2}$⊃(*R*)-G4 in CD$_3$CN. Full (**a**) and partial (**b**–**e**) ROESY spectra (500 MHz, CD$_3$CN, 25 ºC, mixing time = 200 ms) of (*M*)-**1$_{TBA2}$**⊃(*R*)-**G4** (0.40 mM). Intermolecular NOE cross-peaks are indicated by red arrows.

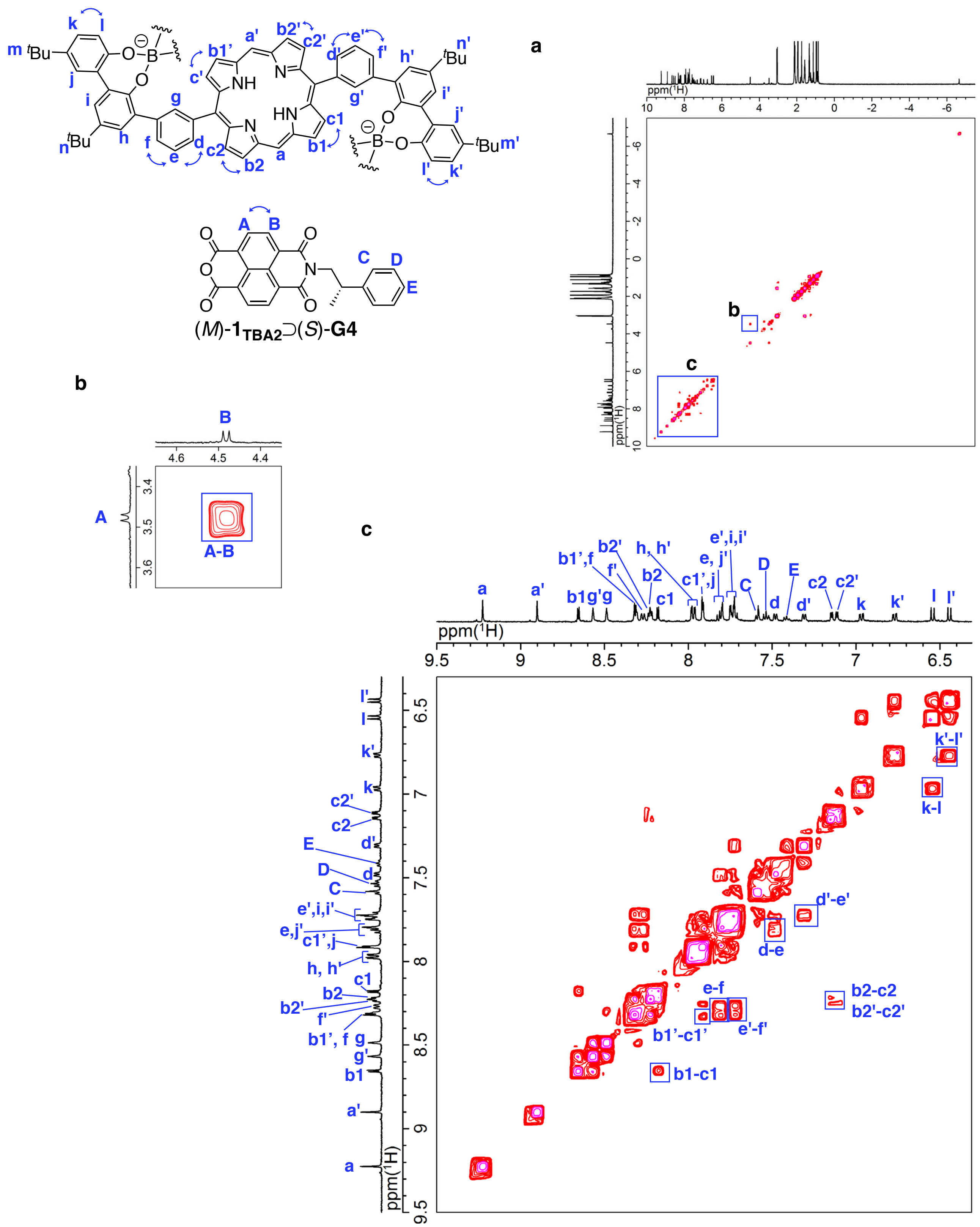

Supplementary Figure 46 | gCOSY spectra of (*M*)-1$_{TBA2}$⊃(*S*)-G4 in CD$_3$CN. Full (**a**) and partial (**b** and **c**) gCOSY spectra (500 MHz, CD$_3$CN, 25 ºC) of (*M*)-**1**$_{TBA2}$⊃(*S*)-**G4** (0.40 mM).

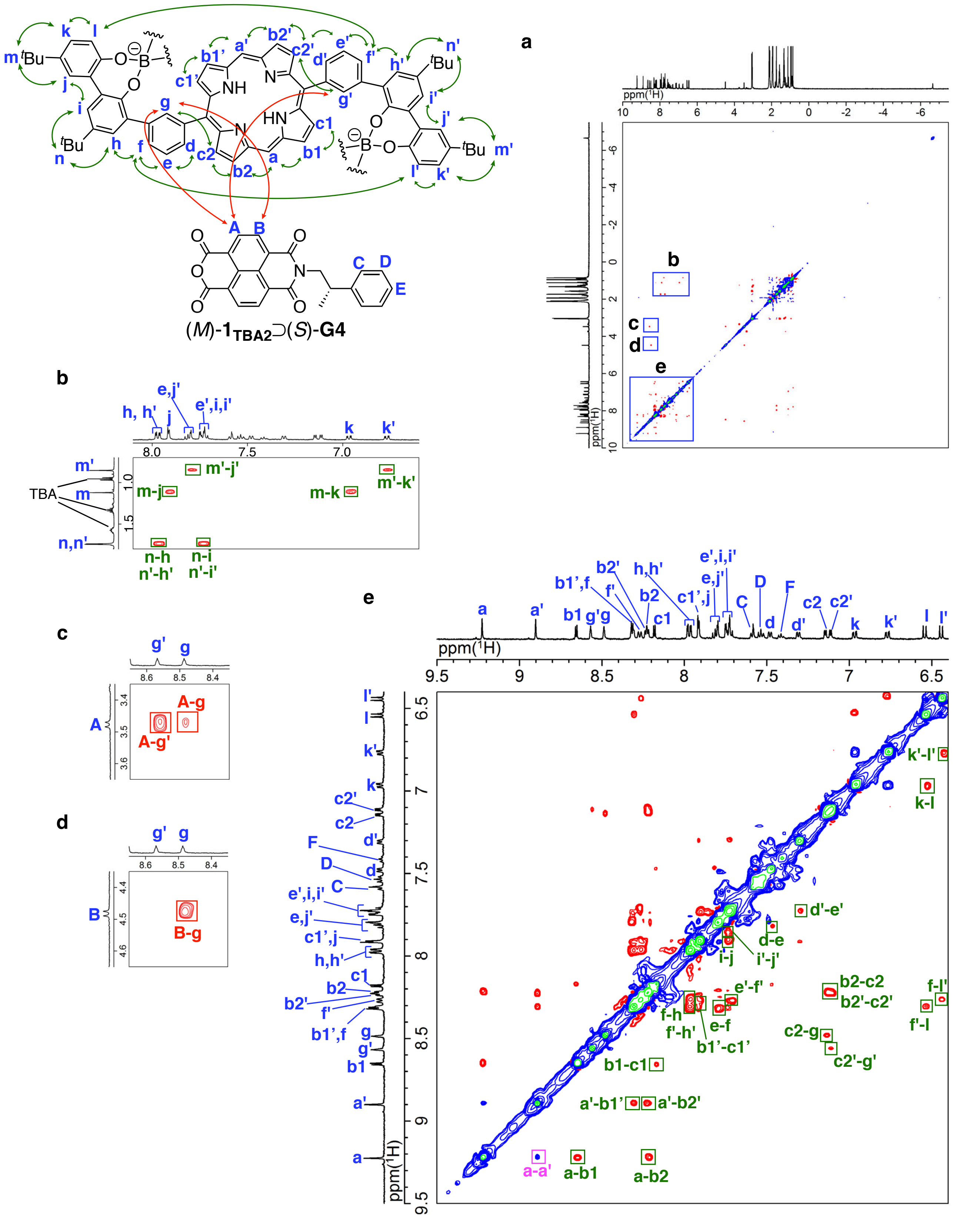

Supplementary Figure 47 | ROESY spectra of (*M*)-1$_{TBA2}$⊃(*S*)-G4 in CD_3CN. Full (**a**) and partial (**b**–**e**) ROESY spectra (500 MHz, CD_3CN, 25 ºC, mixing time = 200 ms) of (*M*)-**1**$_{TBA2}$⊃(*S*)-**G4** (0.40 mM). Intermolecular NOE cross-peaks are indicated by red arrows.

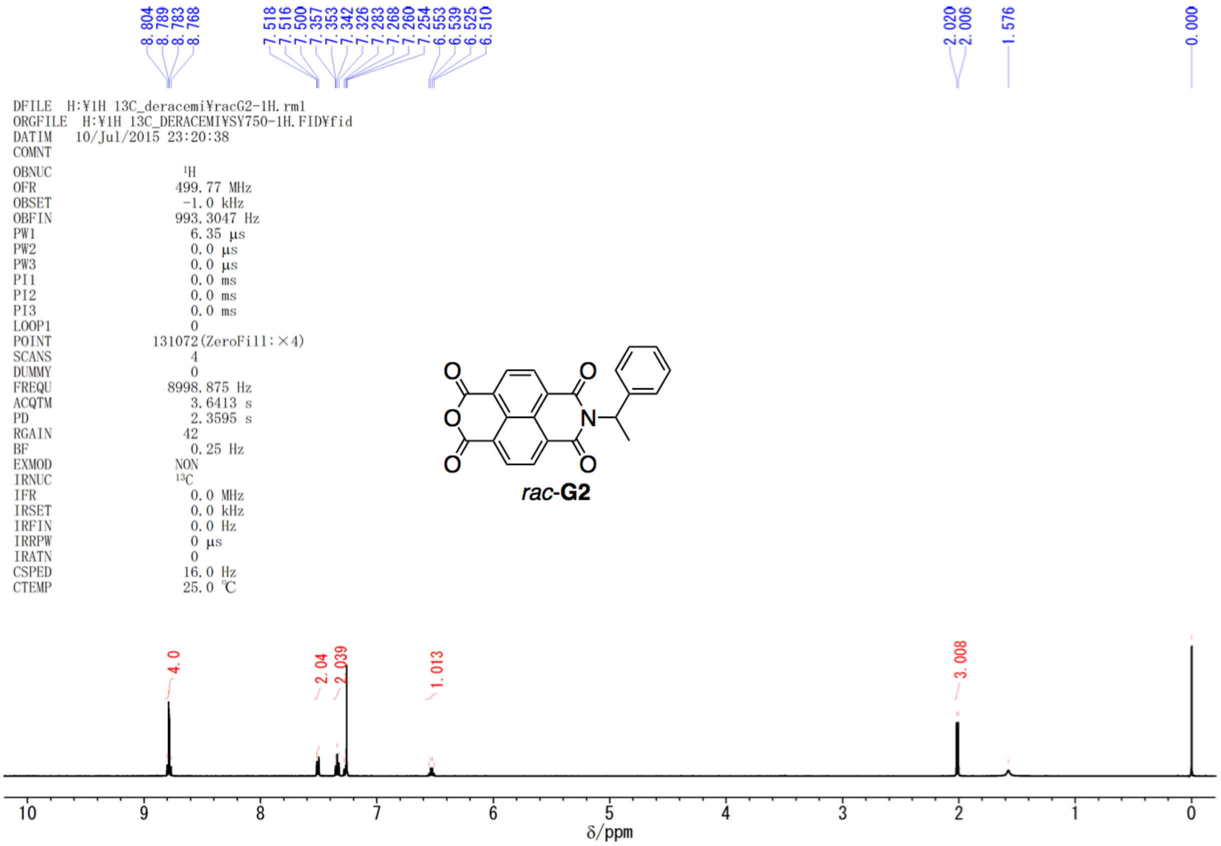

Supplementary Figure 48 | ^{1}H NMR ($CDCl_3$, 500 MHz) spectrum of *rac*-**G2**.

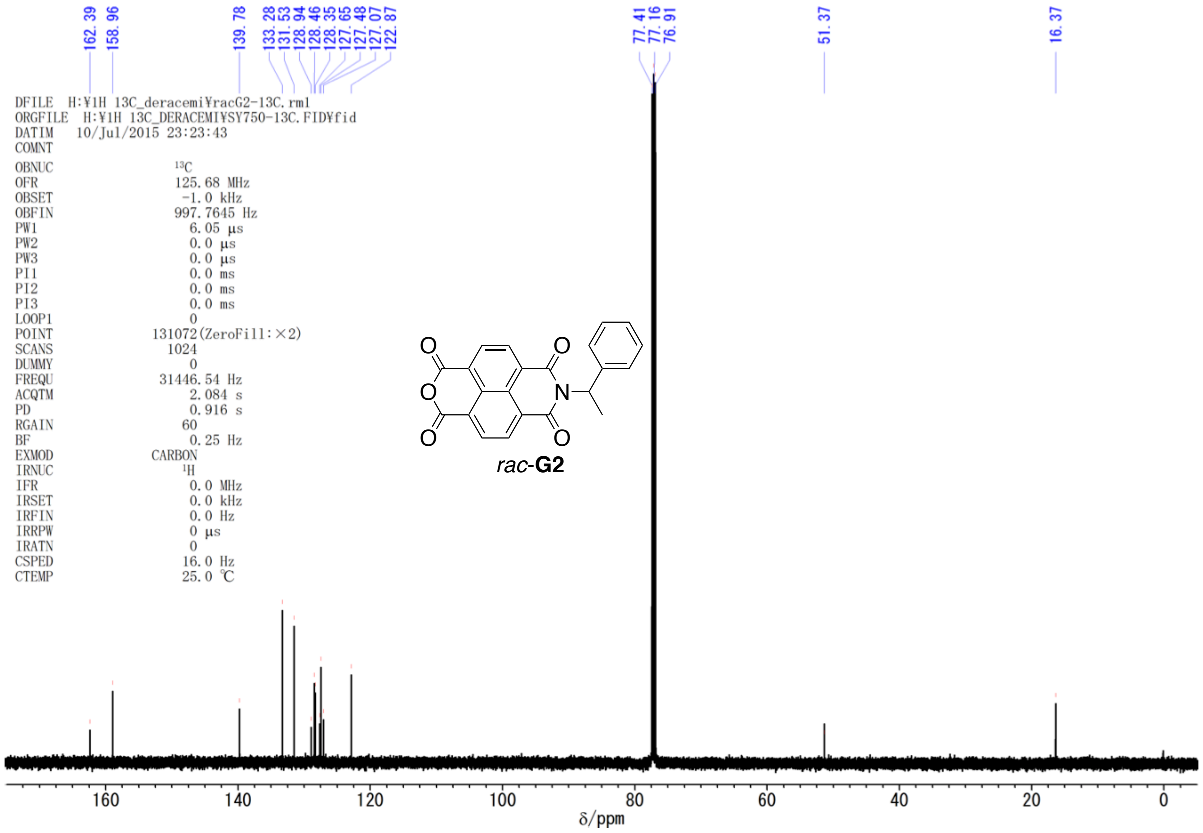

Supplementary Figure 49 | ^{13}C NMR ($CDCl_3$, 125 MHz) spectrum of *rac*-**G2**.

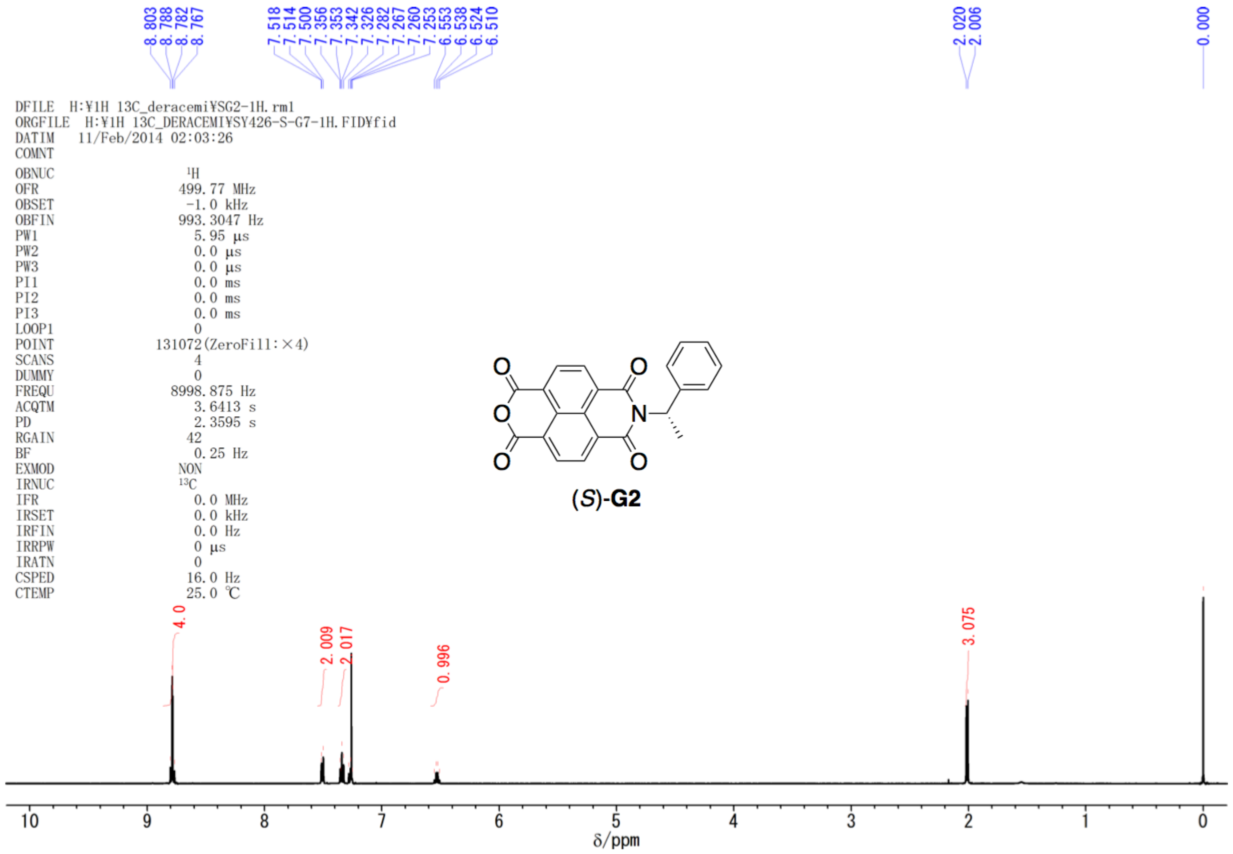

Supplementary Figure 50 | ^{1}H NMR ($CDCl_3$, 500 MHz) spectrum of (*S*)-**G2**.

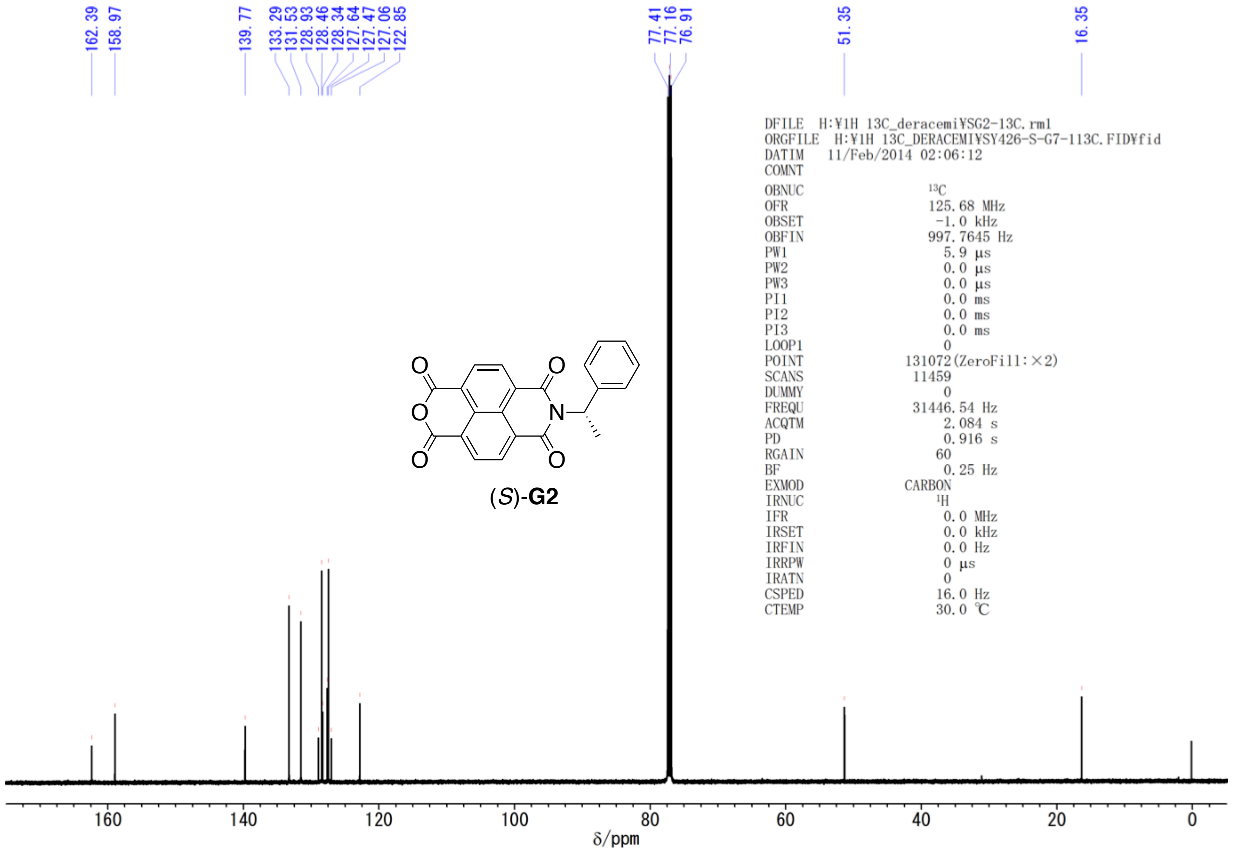

Supplementary Figure 51 | ^{13}C NMR ($CDCl_3$, 125 MHz) spectrum of (*S*)-**G2**.

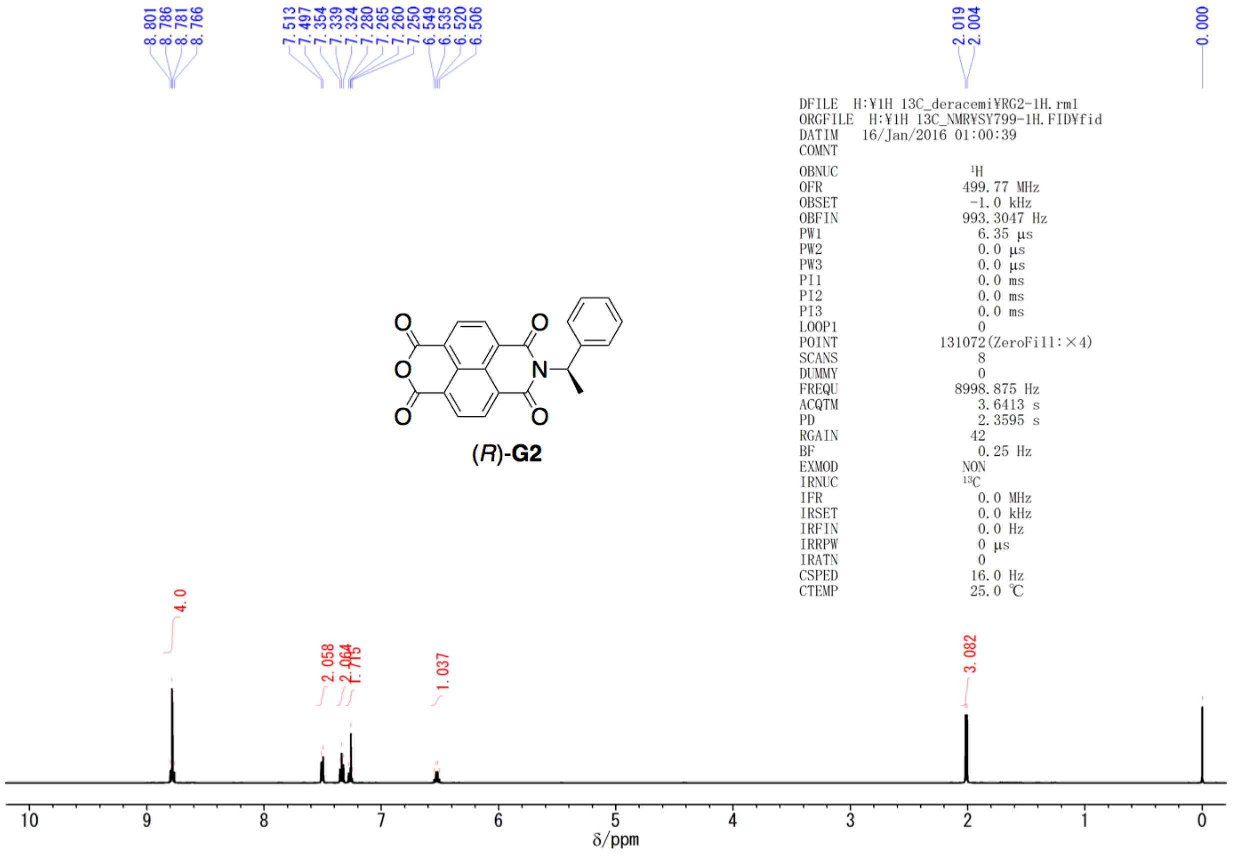

Supplementary Figure 52 | ^{1}H NMR ($CDCl_3$, 500 MHz) spectrum of (*R*)-**G2**.

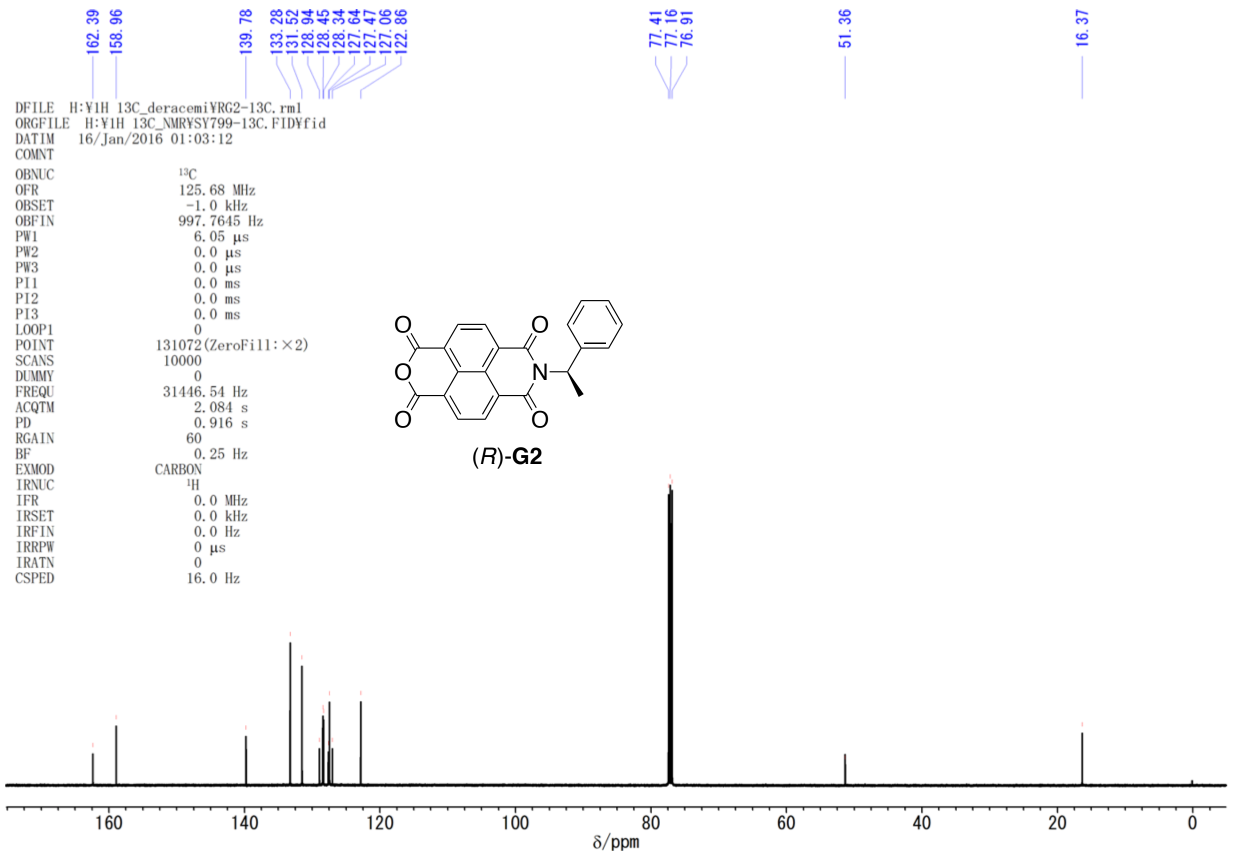

Supplementary Figure 53 | ^{13}C NMR ($CDCl_3$, 125 MHz) spectrum of (*R*)-**G2**.

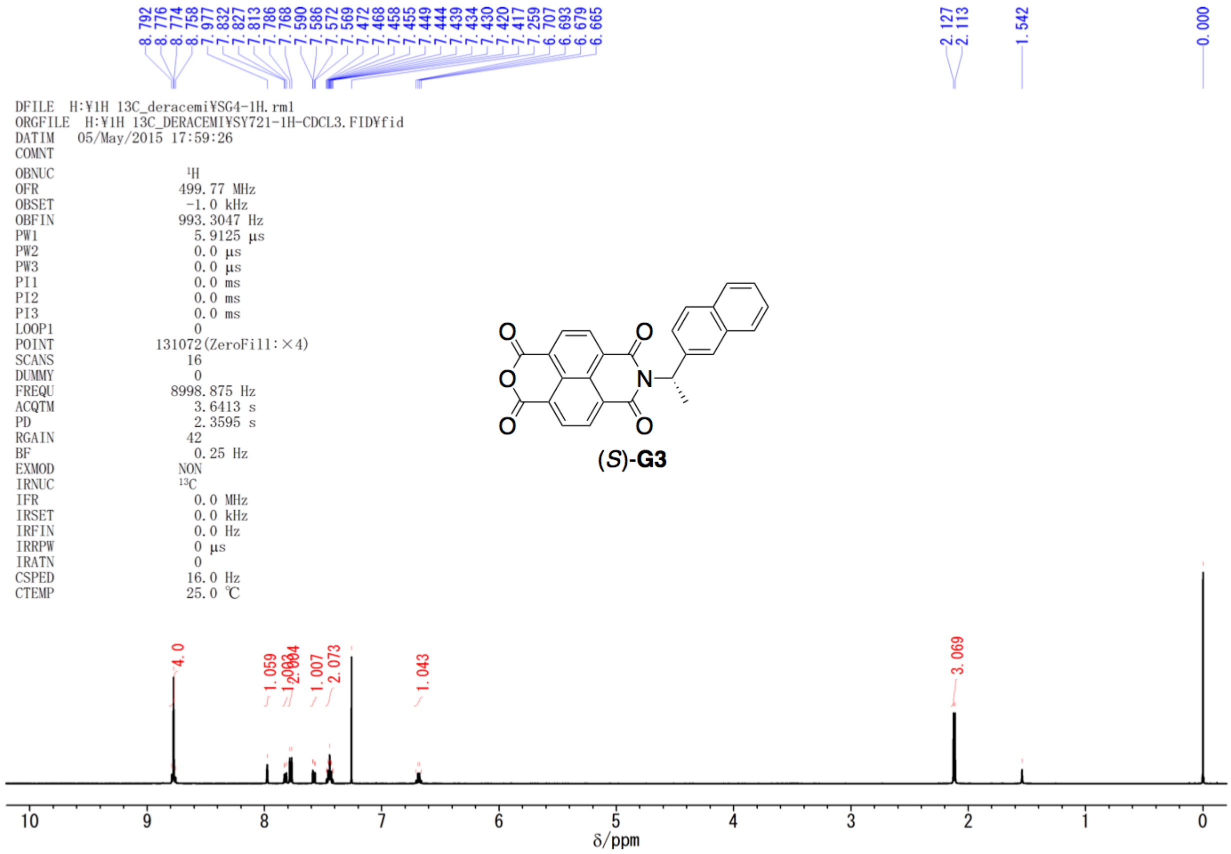

Supplementary Figure 54 | ^{1}H NMR ($CDCl_3$, 500 MHz) spectrum of (*S*)-**G3**.

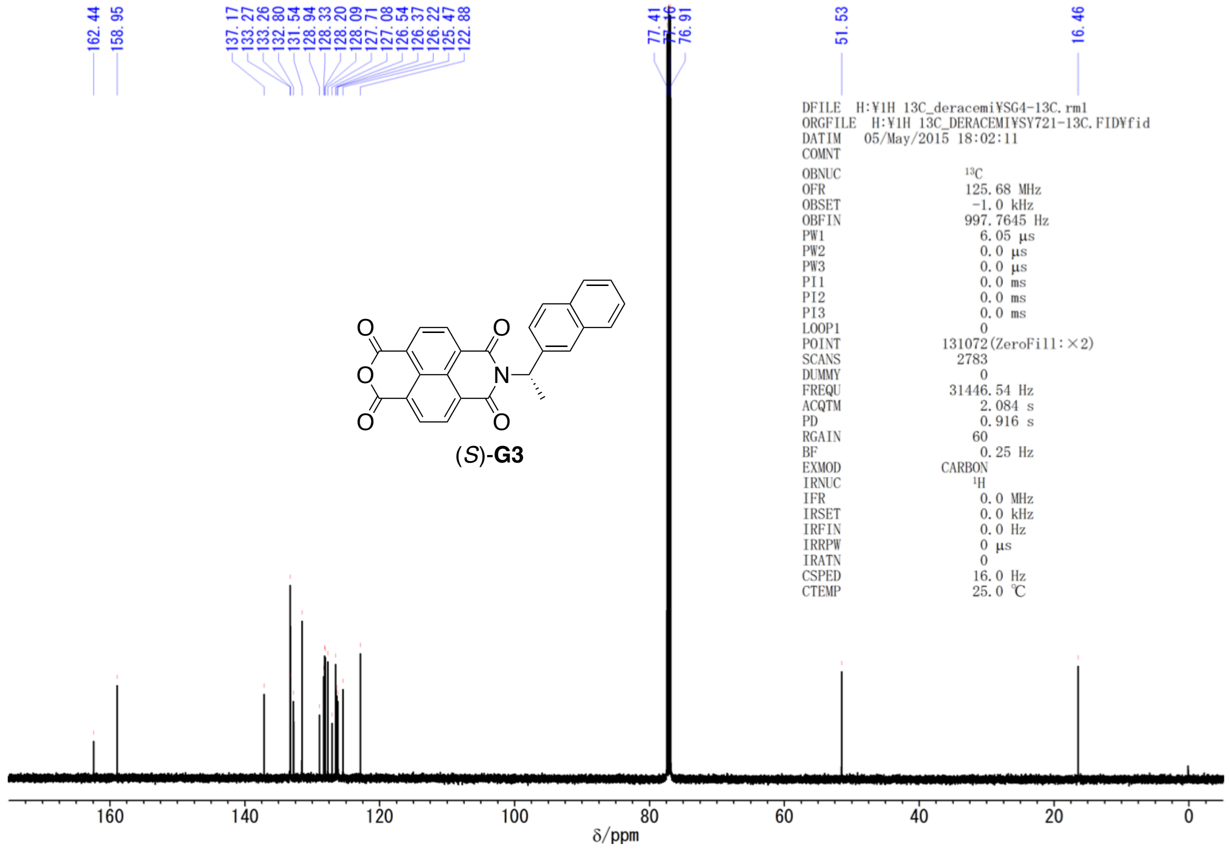

Supplementary Figure 55 | ^{13}C NMR ($CDCl_3$, 125 MHz) spectrum of (*S*)-**G3**.

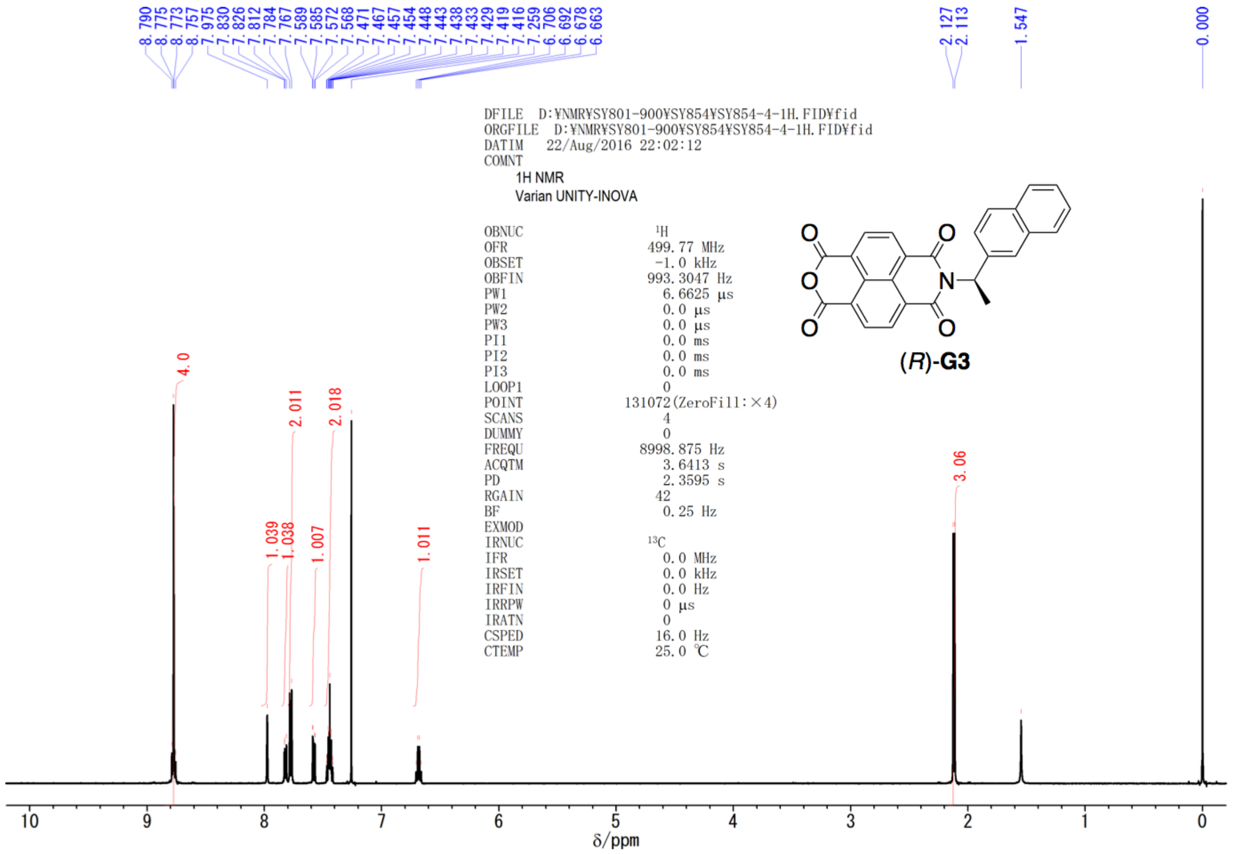

Supplementary Figure 56 | ^{1}H NMR ($CDCl_3$, 500 MHz) spectrum of (*R*)-**G3**.

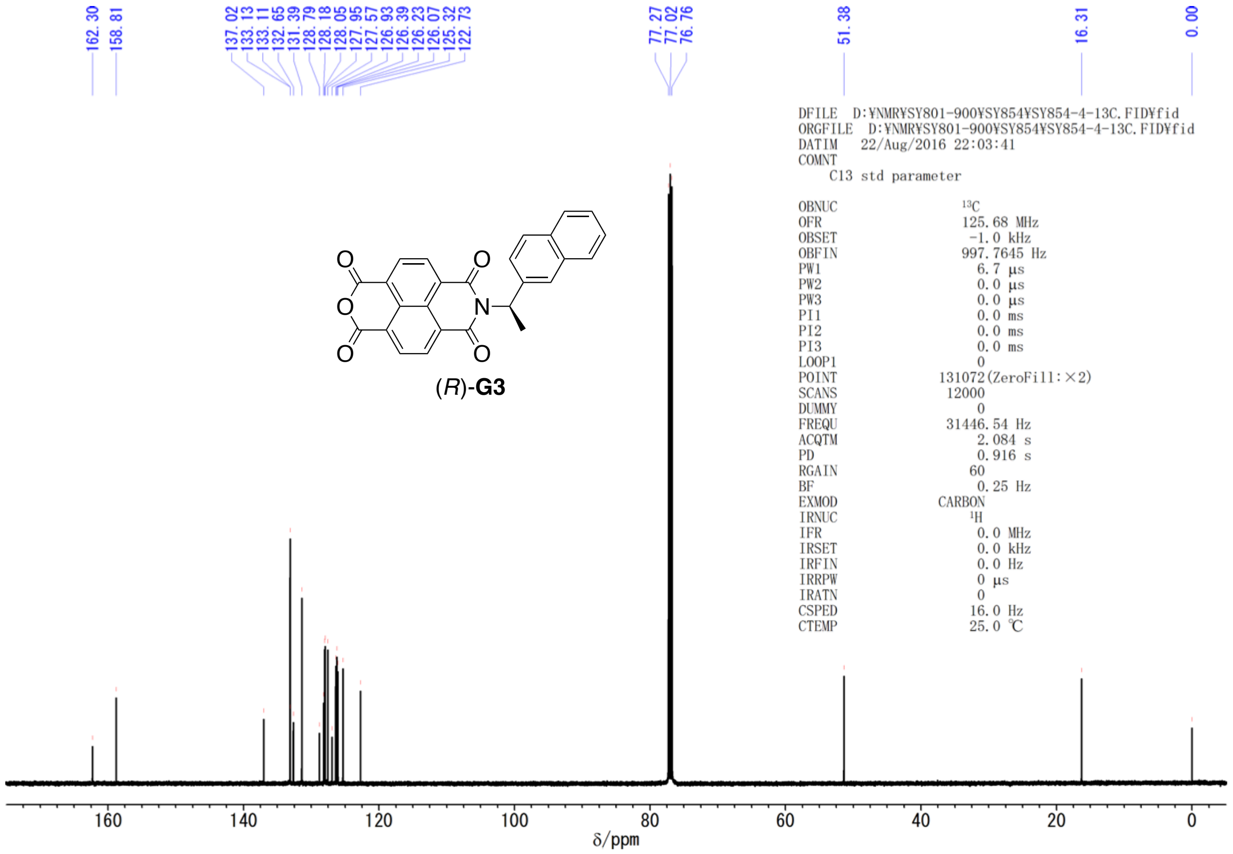

Supplementary Figure 57 | ^{13}C NMR ($CDCl_3$, 125 MHz) spectrum of (*R*)-**G3**.

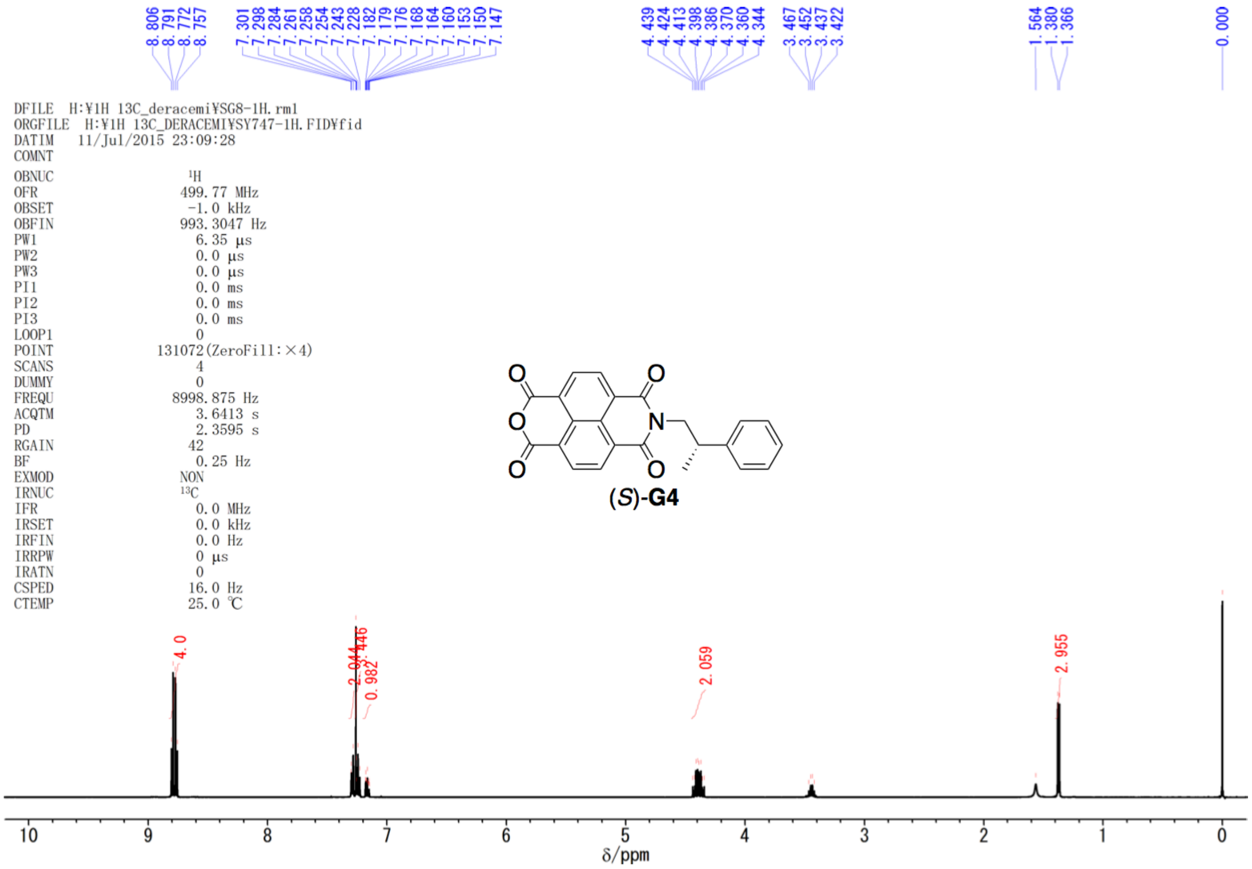

Supplementary Figure 58 | ^{1}H NMR ($CDCl_3$, 500 MHz) spectrum of (*S*)-**G4**.

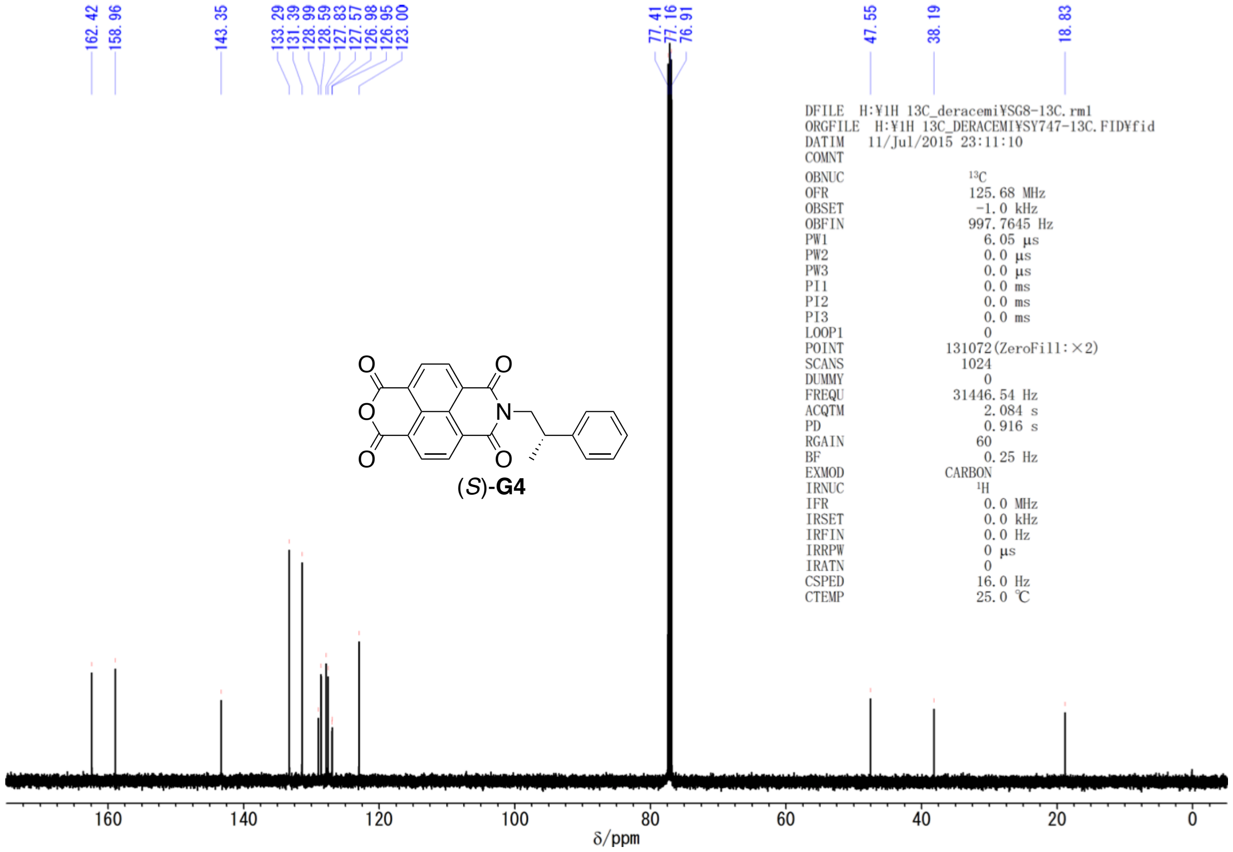

Supplementary Figure 59 | ^{13}C NMR ($CDCl_3$, 125 MHz) spectrum of (*S*)-**G4**.

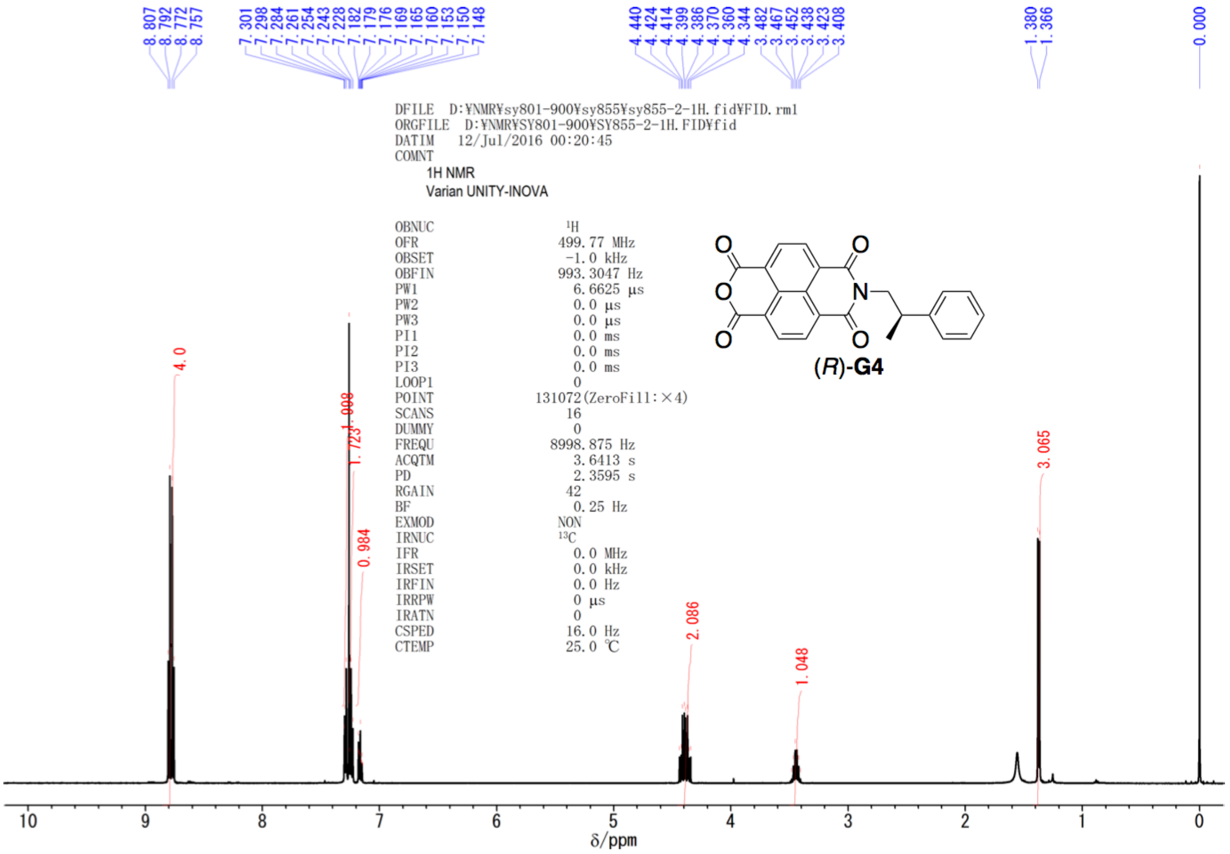

Supplementary Figure 60 | ^{1}H NMR ($CDCl_3$, 500 MHz) spectrum of (*R*)-**G4**.

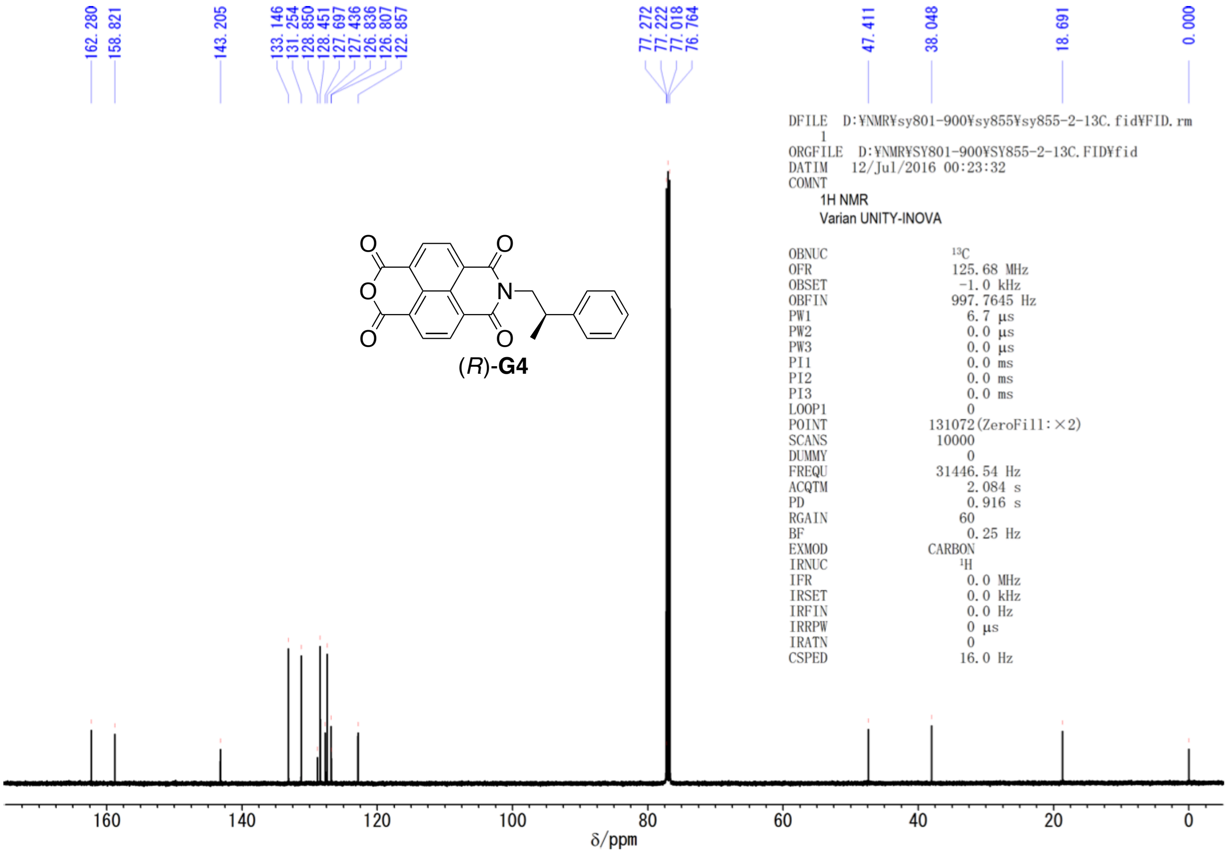

Supplementary Figure 61 | ^{13}C NMR ($CDCl_3$, 125 MHz) spectrum of (*R*)-**G4**.

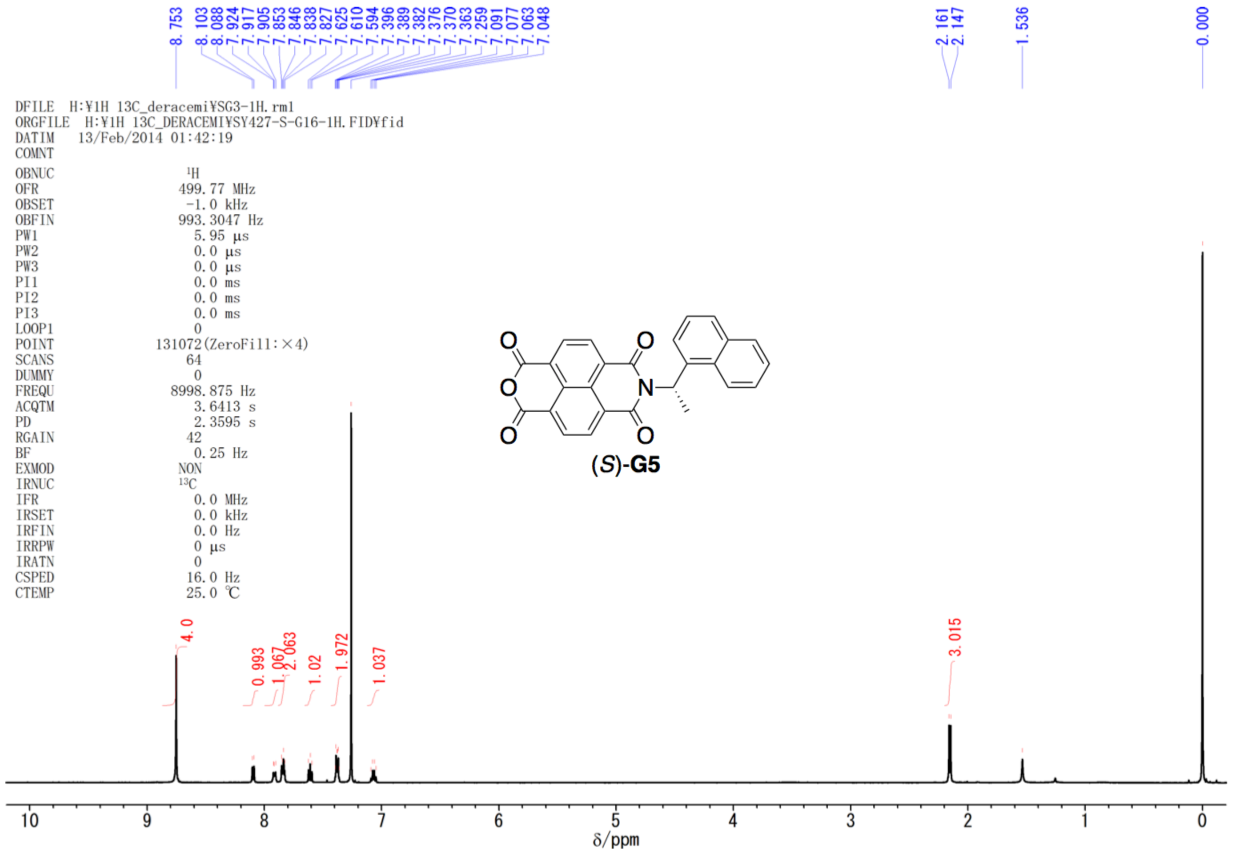

Supplementary Figure 62 | ^{1}H NMR ($CDCl_3$, 500 MHz) spectrum of (*S*)-**G5**.

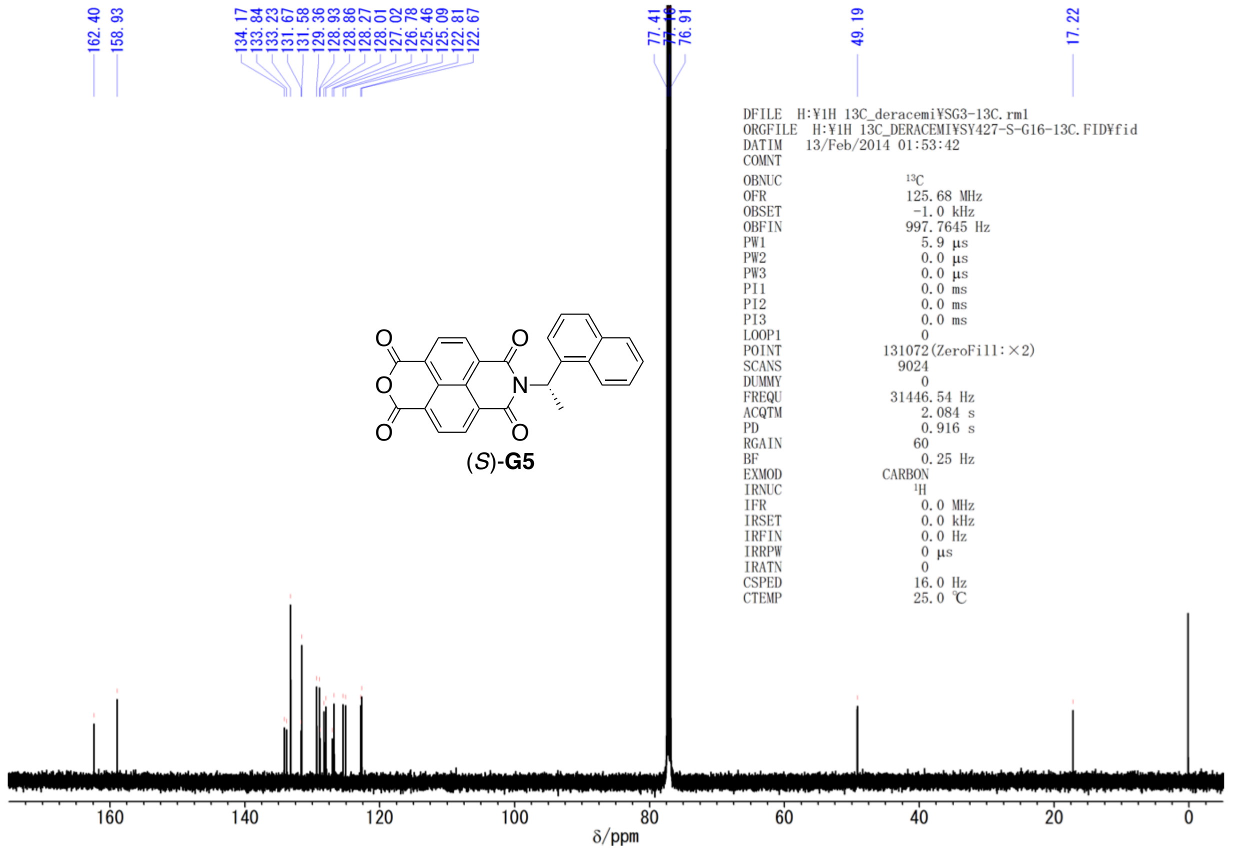

Supplementary Figure 63 | ^{13}C NMR ($CDCl_3$, 125 MHz) spectrum of (*S*)-**G5**.

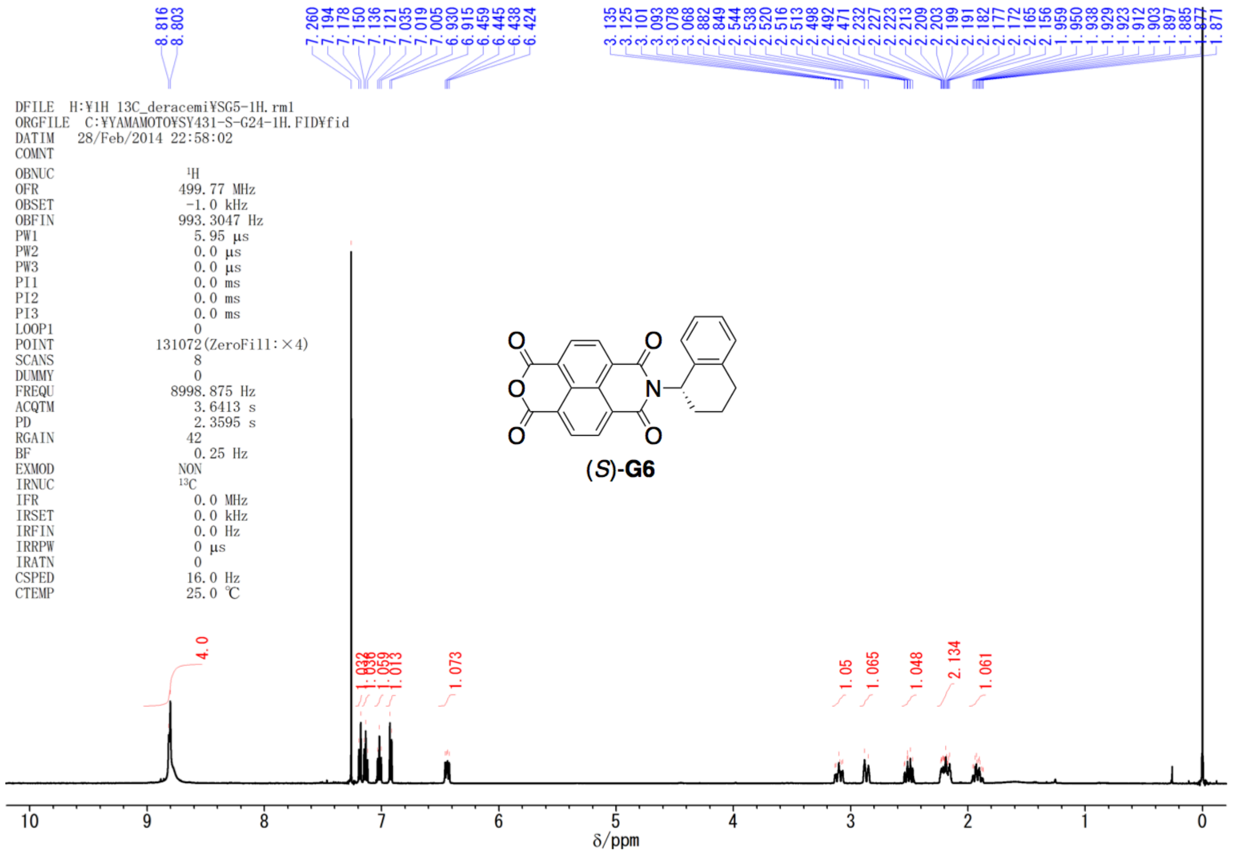

Supplementary Figure 64 | ^{1}H NMR ($CDCl_3$, 500 MHz) spectrum of (*S*)-**G6**.

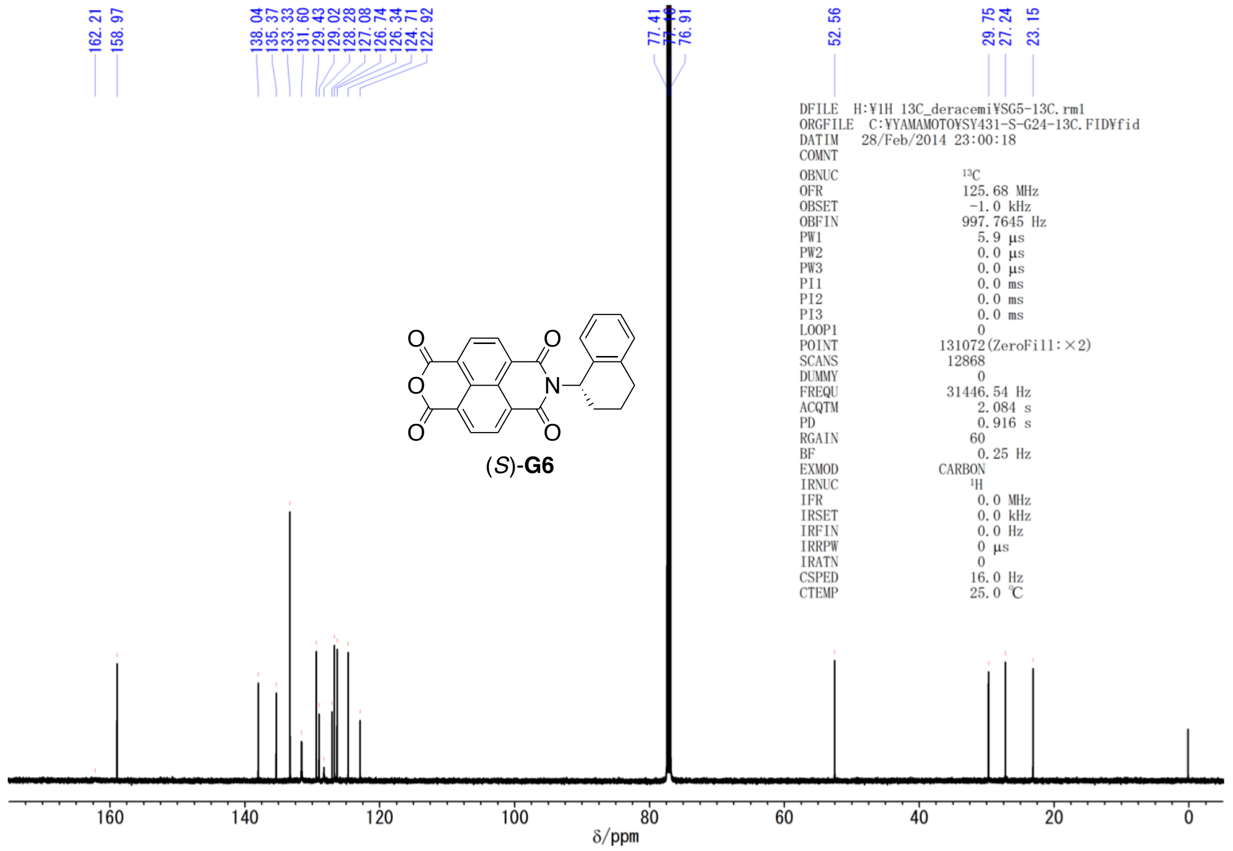

Supplementary Figure 65 | ^{13}C NMR ($CDCl_3$, 125 MHz) spectrum of (*S*)-**G6**.

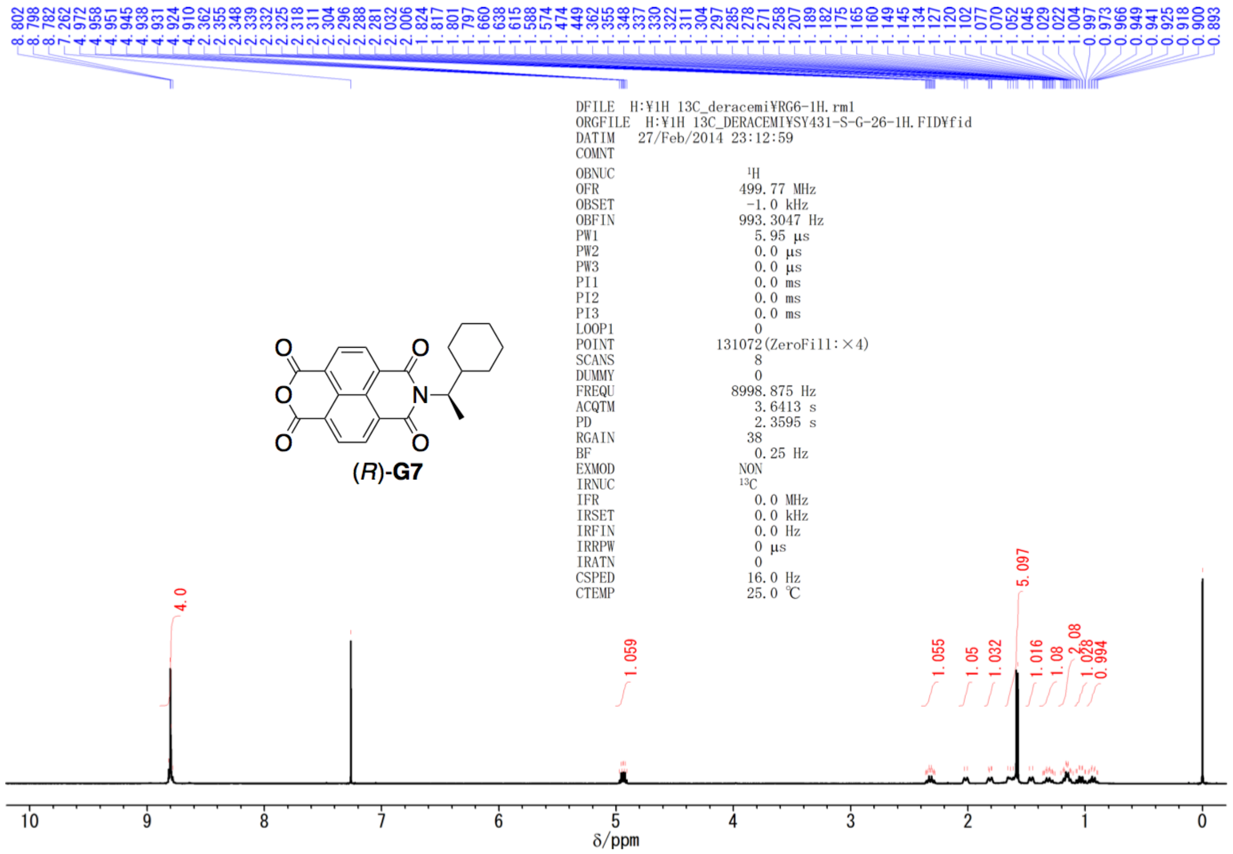

Supplementary Figure 66 | ^{1}H NMR ($CDCl_3$, 500 MHz) spectrum of (*R*)-**G7**.

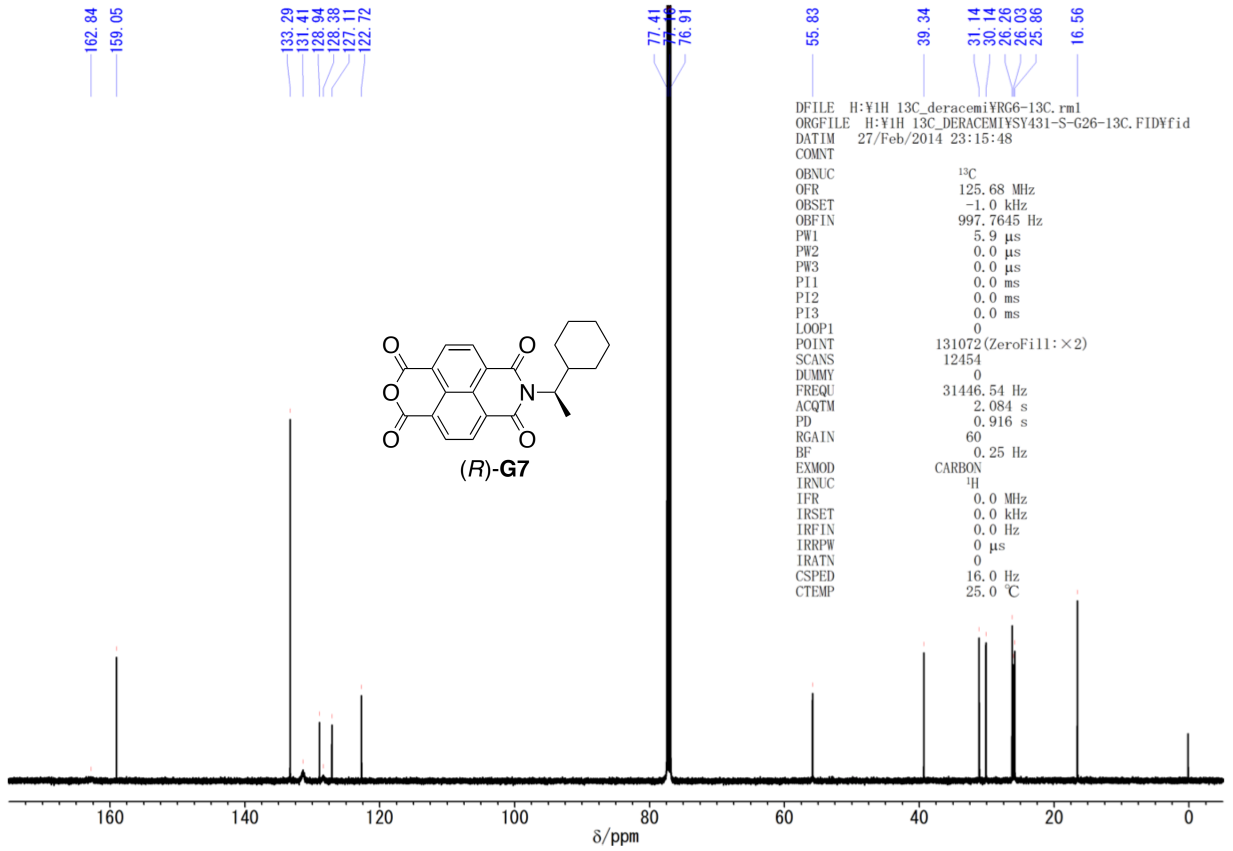

Supplementary Figure 67 | ^{13}C NMR ($CDCl_3$, 125 MHz) spectrum of (*R*)-**G7**.

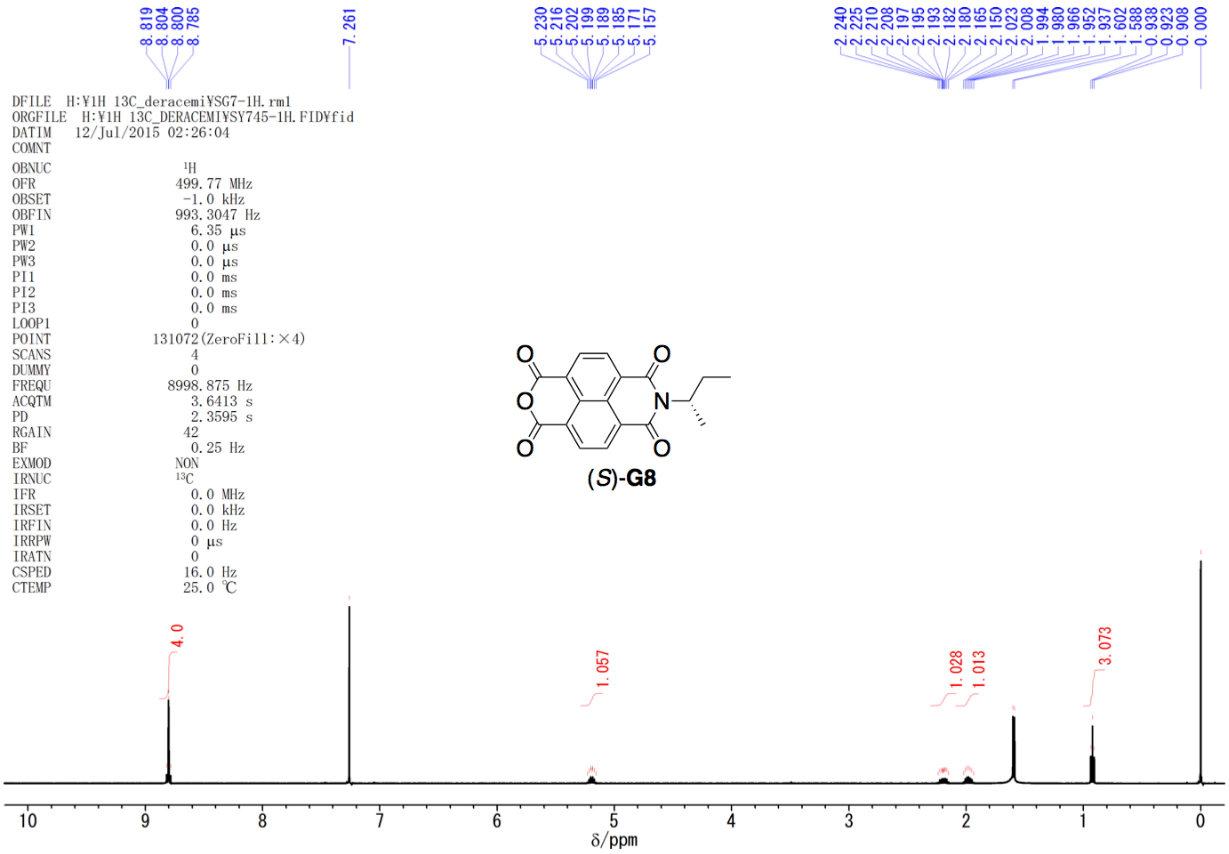

Supplementary Figure 68 | ^{1}H NMR ($CDCl_3$, 500 MHz) spectrum of (*S*)-**G8**.

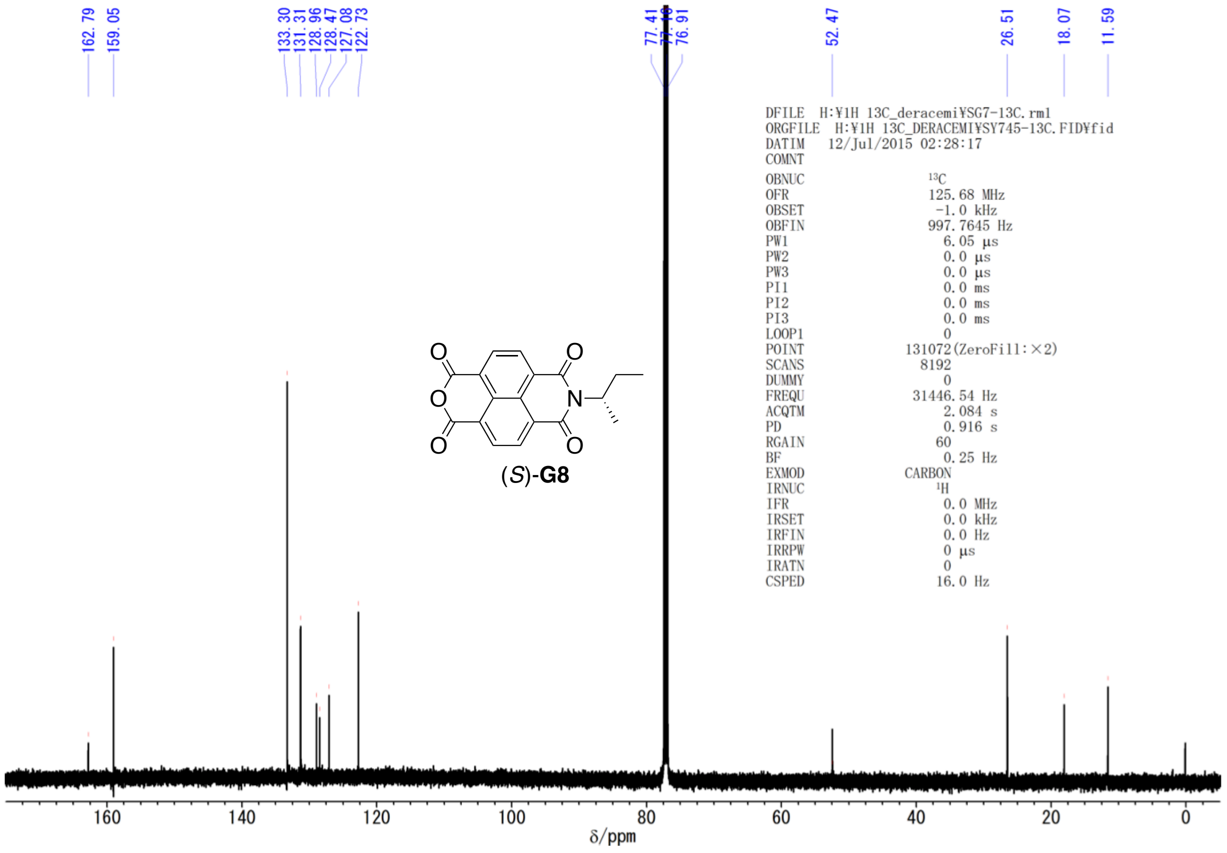

Supplementary Figure 69 | ^{13}C NMR ($CDCl_3$, 125 MHz) spectrum of (*S*)-**G8**.

Supplementary Tables

Supplementary Table 1 | Summary of kinetic and thermodynamic parameters for water-mediated racemization of (*M*)-1_{TBA2} and (*M*)-1_{TBA2}⊃G1 in DMSO containing 1.8 ppm H_2O[a].

Helicate	Temp (ºC)	k_{rac}[b] (s^{-1})	$t_{1/2}$[b] (h)	E_a ($kJmol^{-1}$)	$\Delta G^{‡}_{298}$ ($kJmol^{-1}$)	$\Delta H^{‡}$ ($kJmol^{-1}$)	$\Delta S^{‡}$ ($Jmol^{-1} K^{-1}$)
	70	$(2.07 \pm 0.45) \times 10^{-6}$	46.6				
	80	$(5.87 \pm 0.16) \times 10^{-6}$	16.4				
(*M*)-$\mathbf{1}_{TBA2}$	90	$(1.85 \pm 0.07) \times 10^{-5}$	5.2	111 ± 4	120 ± 1	108 ± 4	-42 ± 11
	100	$(5.02 \pm 0.22) \times 10^{-5}$	1.9				
	110	$(1.06 \pm 0.04) \times 10^{-4}$	0.9				
	70	$(7.40 \pm 0.45) \times 10^{-6}$	13.0				
	80	$(3.08 \pm 0.24) \times 10^{-5}$	3.1				
(*M*)-$\mathbf{1}_{TBA2}$⊃**G1**	90	$(1.16 \pm 0.10) \times 10^{-4}$	0.8	128 ± 5	132 ± 8	125 ± 5	4 ± 20
	100	$(3.46 \pm 0.44) \times 10^{-4}$	0.3				
	110	$(7.99 \pm 1.9) \times 10^{-4}$	0.1				

[a]$[H_2O]$/[(*M*)-$\mathbf{1}_{TBA2}$] = ca. 500.

[b]Average values of three individual measurements.

Supplementary Table 2 | Results of diastereoselective inclusion complexation between (*M*)-1_{TBA2} and racemic guests in various solvents.

Run	Guest	Solvent	Temperature (ºC)	d.e. (%)[a]	ref
1	*rac*-**G2**	CD_3CN	55	8 (*S* rich)	Supplementary Fig.13b
2		CD_3CN	25	8 (*R* rich)	Supplementary Fig.13b
3		CD_3CN	–5	26 (*R* rich)	Supplementary Fig.13b
4		CD_3CN	–35	42 (*R* rich)	Supplementary Fig.13b
5		DMSO-d_6	25	39 (*S* rich)	Supplementary Fig.11a
6		acetone-d_6	25	45 (*S* rich)	Supplementary Fig.11b
7		DMF-d_6	25	48 (*S* rich)	Supplementary Fig.11c
8		THF-d_8	55	54 (*S* rich)	Supplementary Fig.13a
9		THF-d_8	25	66 (*S* rich)	Supplementary Fig.13a
10		THF-d_8	–35	82 (*S* rich)	Supplementary Fig.13a
11		THF-d_8	–75	94 (*S* rich)	Supplementary Fig.13a
12	*rac*-**G3**	CD_3CN	55	16 (*S* rich)	Supplementary Fig.14b
13		CD_3CN	25	6 (*S* rich)	Supplementary Fig.14b
14		CD_3CN	–5	12 (*R* rich)	Supplementary Fig.14b
15		CD_3CN	–20	26 (*R* rich)	Supplementary Fig.14b
16		THF-d_8	55	62 (*S* rich)	Supplementary Fig.14a
17		THF-d_8	25	74 (*S* rich)	Supplementary Fig.14a
18		THF-d_8	–35	98 (*S* rich)	Supplementary Fig.14a
19		THF-d_8	–50	>99 (*S* rich)	Supplementary Fig.14a
20	*rac*-**G4**	CD_3CN	55	20 (*R* rich)	Supplementary Fig.15b
21		CD_3CN	25	26 (*R* rich)	Supplementary Fig.15b
22		CD_3CN	–5	30 (*R* rich)	Supplementary Fig.15b
23		CD_3CN	–35	38 (*R* rich)	Supplementary Fig.15b
24		THF-d_8	40	34 (*S* rich)	Supplementary Fig.15a
25		THF-d_8	25	32 (*S* rich)	Supplementary Fig.15a
26		THF-d_8	–20	26 (*S* rich)	Supplementary Fig.15a
27		THF-d_8	–50	18 (*S* rich)	Supplementary Fig.15a

[a]Estimated by ^{1}H NMR.

Supplementary Table 3 | The stabilization energies (E_{stb}) of the (*S*)- and (*R*)-G2 molecules gained by the inclusion complexation with (*M*)-1^{2-} (SA_{opt}, SB_{opt}, RA_{opt} and RB_{opt}) and the energy differences (ΔE_{stb}) between the most stable structure SA_{opt} and the other structures evaluated by the DFT optimizations with the D3 dispersion correction.

Structure	E_{stb} (kcal mol^{-1})	ΔE_{stb} (kcal mol^{-1})
**SA_{opt}**	–56.2	0.0
**SB_{opt}**	–53.4	+2.8
**RA_{opt}**	–53.5	+2.7
**RB_{opt}**	–54.3	+1.9

Supplementary Table 4| The interaction energies (E_{int}) of the (*S*)- and (*R*)-G2 molecules gained by the inclusion complexation with (*M*)-1^{2-} (SA_{opt}, SB_{opt}, RA_{opt} and RB_{opt}) and the energy differences (ΔE_{int}) between the most stable structure SA_{opt} and the other structures evaluated by the DFT single point calculations without the D3 dispersion correction.

Structure	E_{int} (kcal mol^{-1})	ΔE_{int} (kcal mol^{-1})
**SA_{opt}**	+0.042	0.0
**SB_{opt}**	+1.0	+1.0
**RA_{opt}**	+2.5	+2.5
**RB_{opt}**	+2.0	+1.9

Supplementary Table 5 | Chemical shifts of proton singals of various solvents in $CDCl_3$ and C_6D_6 and their chemical shift differences ($\Delta\delta_{sol}$).

Solvent	δ (in $CDCl_3$)	δ (in C_6D_6)	$\Delta\delta_{sol}$[c]
CH_3CN[a]	2.1	0.58	1.52
DMSO[a]	2.62	1.68	0.94
DMF[a]	8.02<br>2.96<br>2.88	7.63<br>2.36<br>1.86	0.67[d]
acetone[a]	2.17	1.55	0.62
THF[a]	1.85<br>3.76	1.4<br>3.57	0.32[d]
IBN[b]	2.70<br>1.33	1.73<br>0.59	0.86[d]
DMAc[a]	2.09<br>3.02<br>0.94	1.6<br>2.57<br>2.05	0.61[d]
DEF[b]	8.05<br>3.37<br>3.28<br>1.19<br>1.13	7.80<br>3.05<br>2.40<br>0.82<br>0.51	0.49[d]
DIPF[b]	8.21<br>4.19<br>3.61<br>1.29<br>1.26	8.06<br>3.71<br>2.87<br>1.12<br>0.68	0.42[d]

[a]The chemical shifts (ppm) of solvent signals in $CDCl_3$ and C_6D_6 were taken from references 2 and 3, respectively.

[b]The chemical shifts (ppm) of solvent signals in $CDCl_3$ and C_6D_6 were detrmined by measureing the ^1H NMR spectra of the solvents (0.1 vol%) in $CDCl_3$ and C_6D_6, respectively.

[c]$\Delta\delta = \delta$ (solvent signal in $CDCl_3$) – δ (solvent signal in C_6D_6).

[d]Average value.

Supplementary Methods

Instruments and Materials. The melting points were measured on an OptiMelt micromelting point apparatus (SRS, Sunnyvale, USA) and were uncorrected. The IR spectra were recorded on a JASCO FT/IR-680 spectrophotometer (JASCO, Tokyo, Japan). The NMR spectra were measured using a Bruker Ascend 500 (Bruker Biospin, Billerica, MA) or a Varian 500AS (Varian, Palo Alto, CA) spectrometer operating at 500 MHz for ^{1}H and 125 MHz for ^{13}C using tetramethylsilane (TMS) or a residual undeuterated solvent peak as the internal standard. The absorption and CD spectra were measured in a 0.1-, or 1- cm quartz cell using a JASCO V-570 spectrophotometer and a JASCO J-820 or J-1500 spectropolarimeter, respectively. The temperature was controlled with a JASCO PTC-423L apparatus. The fluorescence spectra were measured in a 1-cm quartz cell on a JASCO FP-6500 spectrofluorometer. The electrospray ionization (ESI) mass spectra were recorded using a JEOL JMS-T100CS mass spectrometer (JEOL, Akishima, Japan). The SEC fractionations were performed using an LC-908W-C60 liquid chromatograph (Japan Analytical Industry) equipped with two SEC columns (JAIGEL-1H-40 (4 (i.d.) × 60 cm) and JAIGEL-2H-40 (4 (i.d.) × 60 cm)) in series and UV-visible (JAI UV-3702) and RI (JAI RI-5) detectors; chloroform ($CHCl_3$) was used as the eluent at a flow rate of 12 mL min^{-1}. The analytical SEC measurements were performed with a JASCO PU-980 liquid chromatograph equipped with an SEC column (TSKgel α 2500 (TOSOH, 0.78 (i.d.) × 30 cm)) and a UV-visible detector (375 nm, JASCO UV-2075 plus); *N*,*N*-dimethylformamide (DMF) was used as the eluent at a flow rate of 0.6 mL/min.

All starting materials were purchased from commercial suppliers and were used without further purification unless otherwise noted. Isobutyronitrile (IBN), DMF, *N*,*N*-diethylformamide (DEF), *N*,*N*-diisopropylformamide (DIPF), and *N*,*N*-dimethylacetamide (DMAc) were dried over molecular sieves (3A or 4A) and freshly distilled prior to use. Deuterated solvents (DMSO-d_6, CD_3CN, acetone-d_6, and THF-d_8) were dried over molecular sieves (3A or 4A) and used without distillation. The spiroborate helicate *rac*-$\mathbf{1}_{\mathbf{Na2}}$ and its enantiomers complexed with an achiral tetra-*n*-butylammonium (TBA) cation, (*P*)- and (*M*)-$\mathbf{1}_{\mathbf{TBA2}}$, were prepared according to the previously reported method.[1]

Synthesis of Naphthalene-Based Chiral Guests.

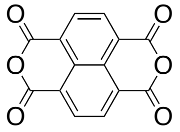

G1

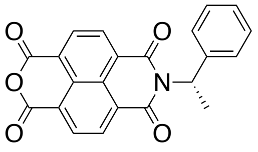

(*S*)-**G2**

(*S*)-**G2.** To a solution of **G1** (500 mg, 1.85 mmol) in DMF (20 mL) was added a solution of (*S*)-1-phenylethylamine (0.23 mL, 1.9 mmol) in DMF (5 mL) at 80 ºC under argon. The reaction mixture was stirred at 150 ºC for 10 min, and then at 100 ºC for 9 h. After cooling to ambient temperature, the solvent was removed under reduced pressure. The resulting brown residue was then suspended in acetone, and the suspension was slowly poured into a MeOH/H_2O (1/1, v/v) mixture under vigorous stirring. The precipitate was collected and dissolved in $CHCl_3$, and the solution was dried over anhydrous Na_2SO_4, filtered, and concentrated under reduce pressure. The crude product was purified by SEC ($CHCl_3$ as eluent), affording (*S*)-**G2** (274 mg, 40% yield) as a pale yellow solid. Mp: 211.9–212.8 °C. IR (KBr, cm^{-1}): 1786 ($\nu_{C=O}$), 1748 ($\nu_{C=O}$), 1710 ($\nu_{C=O}$), 1671 ($\nu_{C=O}$). ^{1}H NMR (500 MHz, $CDCl_3$, 25 °C): δ 8.79 (d, 2H, J = 7.5 Hz, ArH), 8.77 (d, J = 7.5 Hz, 2H, ArH), 7.51 (dd, J = 7.0, 2.0 Hz, 2H, ArH), 7.34 (br dd, 2H, ArH), 7.27 (br tt, 1H, ArH), 6.53 (q, 1H, J = 7.0 Hz, CH), 2.01 (d, 3H, J = 7.0 Hz, CH_3). ^{13}C NMR (125 MHz, $CDCl_3$, 25 °C): δ 162.39, 158.97, 139.77, 133.29, 131.53, 128.93, 128.46, 128.34, 127.64, 127.47, 127.06, 122.85, 51.35, 16.35. Anal. Calcd (%) for ($C_{22}H_{13}NO_5$): C, 71.16; H, 3.53; N, 3.77. Found: C, 71.14; H, 3.49; N, 3.80.

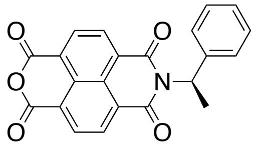

(*R*)-**G2**

(*R*)-**G2.** (*R*)-**G2** was prepared in the same procedure to that for (*S*)-**G2** in 44% yield (182 mg). Mp: 211.6–212.9 °C. IR (KBr, cm^{-1}): 1785 ($\nu_{C=O}$), 1746 ($\nu_{C=O}$), 1710 ($\nu_{C=O}$), 1671 ($\nu_{C=O}$). ^{1}H NMR (500 MHz, $CDCl_3$, 25 °C): δ 8.79 (d, 2H, J = 7.5 Hz, ArH), 8.77 (d, 2H, J = 7.5 Hz, ArH), 7.51 (br dd, 2H, ArH), 7.34 (br dd, 2H, ArH), 7.27 (br tt, 1H, ArH), 6.53 (q, 1H, J = 7.0 Hz, CH), 2.01 (d, 3H, J = 7.0 Hz, CH_3). ^{13}C NMR (125 MHz, $CDCl_3$, 25 °C): δ 162.39, 158.96, 139.78, 133.28, 131.52, 128.94, 128.45, 128.34, 127.64, 127.47, 127.06, 122.86, 51.39, 16.37. Anal. Calcd (%) for ($C_{22}H_{13}NO_5$): C, 71.16; H, 3.53; N, 3.77. Found: C, 71.16; H, 3.41; N, 3.76.

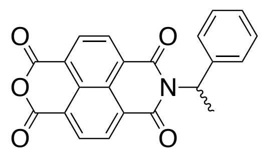

rac-**G2**

rac-**G2.** *rac*-**G2** was also prepared in the same procedure to that for (*S*)- or (*R*)-**G2** (244 mg, 41% yield). Mp: 274.5–276.6 °C. IR (KBr, cm^{-1}): 1783 ($\nu_{C=O}$), 1742 ($\nu_{C=O}$), 1707 ($\nu_{C=O}$), 1671 ($\nu_{C=O}$). ^{1}H NMR (500 MHz, $CDCl_3$, 25 °C): δ 8.79 (d, 2H, J = 7.5 Hz, ArH), 8.77 (d, 2H, J = 7.5 Hz, ArH), 7.52 (br dd, 2H, ArH), 7.35 (br dd, 2H, ArH), 7.27 (br tt, 1H, ArH), 6.53 (q, 1H, J = 7.0 Hz, CH), 2.01 (d, 3H, J = 7.0 Hz, CH_3). ^{13}C NMR (125 MHz, $CDCl_3$, 25 °C): δ 162.39, 158.96, 139.78, 133.28, 131.53, 128.94, 128.46, 128.35, 127.65, 127.48, 127.07, 122.87, 51.37, 16.37. Anal. Calcd (%) for ($C_{22}H_{13}NO_5$): C, 71.16; H, 3.53; N, 3.77. Found: C, 71.16; H, 3.49; N, 3.81.

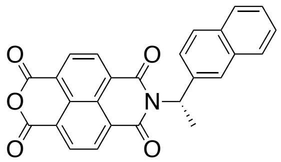

(*S*)-**G3**

(*S*)-**G3.** To a solution of **G1** (500 mg, 1.86 mmol) in DMF (20 mL) was added a solution of (*S*)-1-(2-naphthyl)ethylamine (319 mg, 1.86 mmol) in DMF (10 mL) at 140 ºC under argon and the reaction mixture was stirred at 140 ºC for 40 min. After cooling to ambient temperature, the solvent was removed under reduced pressure. The residue was then dissolved in $CHCl_3$, and the solution was dried over anhydrous Na_2SO_4, filtered, and concentrated under reduce pressure. The crude product was purified by SEC ($CHCl_3$ as eluent), affording (*S*)-**G3** (351 mg, 45% yield) as a yellow solid. Mp: 261.7–262.8 °C. IR (KBr, cm^{-1}): 1785 ($\nu_{C=O}$), 1747 ($\nu_{C=O}$), 1709 ($\nu_{C=O}$), 1670 ($\nu_{C=O}$). ^{1}H NMR (500 MHz, $CDCl_3$, 25 °C): δ 8.78 (d, 2H, J = 8.0 Hz, ArH), 8.76 (d, 2H, J = 8.0 Hz, ArH), 7.98 (s, 1H, ArH), 7.83 (m, 1H, ArH), 7.77 (m, 2H, ArH), 7.58 (m, 1H, ArH), 7.44 (m, 2H, ArH), 6.68 (q, 1H, J = 7.0 Hz, CH), 2.11 (d, 3H, J = 7.0 Hz, CH_3). ^{13}C NMR (125 MHz, $CDCl_3$, 25 °C): δ 162.44, 158.95, 137.17, 133.27, 133.26, 132.80, 131.54, 128.94, 128.33, 128.20, 128.09, 127.71, 127.08, 126.54, 126.47, 126.22, 125.47, 122.88, 51.53, 16.46. Anal. Calcd (%) for ($C_{26}H_{15}NO_5$): C, 74.11; H, 3.59; N, 3.32. Found: C, 74.08; H, 3.46; N, 3.43.

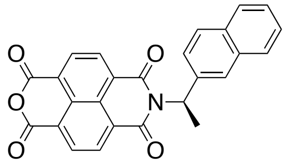

(*R*)-**G3**

(*R*)-**G3.** (*R*)-**G3** was prepared in the same procedure to that for (*S*)-**G3** in 21% yield (99.9 mg). Mp: 261.5–262.8 °C. IR (KBr, cm^{-1}): 1784 ($\nu_{C=O}$), 1747 ($\nu_{C=O}$), 1709 ($\nu_{C=O}$), 1670 ($\nu_{C=O}$). ^{1}H NMR (500 MHz, $CDCl_3$, 25 °C): δ 8.78 (d, 2H, J = 8.0 Hz, ArH), 8.76 (d, 2H, J = 8.0 Hz, ArH), 7.98 (s, 1H, ArH), 7.83 (m, 1H, ArH), 7.76 (m, 2H, ArH), 7.58 (m, 1H, ArH), 7.44 (m, 2H, ArH), 6.68 (q, 1H, J = 7.0 Hz, CH), 2.12 (d, 3H, J = 7.0 Hz, CH_3). ^{13}C NMR (125 MHz, $CDCl_3$, 25 °C): δ 162.30, 158.81, 137.02, 133.13, 133.11, 132.65, 131.39, 128.79, 128.18, 128.05, 127.95, 127.57, 126.93, 126.39, 126.23, 126.07, 125.32, 122.73, 51.38, 16.31. Anal. Calcd (%) for ($C_{26}H_{15}NO_5$): C, 74.11; H, 3.59; N, 3.32. Found: C, 74.14; H, 3.58; N, 3.29.

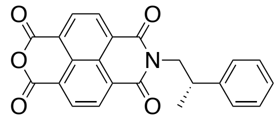

(*S*)-**G4**

(*S*)-**G4.** To a solution of **G1** (500 mg, 1.86 mmol) in DMF (25 mL) was added (*S*)-(–)-β-methylphenethylamine (0.37 mL, 2.6 mmol) at 150 ºC under argon. The reaction mixture was stirred at 180 ºC for 15 min and further at 100 ºC for 27 h. After cooling to ambient temperature, the solvent was removed under reduced pressure. The residue was washed with *n*-hexane, dried under vacuum, and purified by SEC ($CHCl_3$ as eluent), affording (*S*)-**G4** (134 mg, 35% yield) as a pale orange solid. Mp: 258.5–261.6 °C. IR (KBr, cm^{-1}): 1780 ($\nu_{C=O}$), 1736 ($\nu_{C=O}$), 1703 ($\nu_{C=O}$), 1667 ($\nu_{C=O}$). ^{1}H NMR (500 MHz, $CDCl_3$, 25 °C): δ 8.79 (d, 2H, J = 7.5 Hz, ArH), 8.76 (d, 2H, J = 7.5 Hz, ArH), 7.30 (m, 2H, ArH), 7.25 (m, 2H, ArH), 7.16 (m, 1H, ArH), 4.39 (m, 2H, CH_2), 3.44 (m, 1H, CH), 1.37 (d, 3H, J = 7.0 Hz, CH_3). ^{13}C NMR (125 MHz, $CDCl_3$, 25 °C): δ 162.42, 158.96, 143.35, 133.29, 131.39, 128.99, 128.59, 127.83, 127.57, 126.98, 126.95, 123.00, 47.55, 38.19, 18.83. Anal. Calcd (%) for ($C_{23}H_{15}NO_5$): C, 71.68; H, 3.92; N, 3.63. Found: C, 71.67; H, 4.08; N, 3.62.

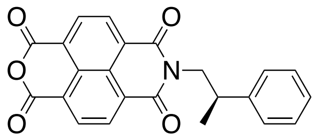

(*R*)-**G4**

(*R*)-**G4.** (*R*)-**G4** was prepared in the same procedure to that for (*S*)-**G4** in 23% yield (101 mg). Mp: 259.4–261.5 °C. IR (KBr, cm^{-1}): 1780 ($\nu_{C=O}$), 1735 ($\nu_{C=O}$), 1703 ($\nu_{C=O}$), 1666 ($\nu_{C=O}$). ^{1}H NMR (500 MHz, $CDCl_3$, 25 °C): δ 8.79 (d, 2H, J = 7.5 Hz, ArH), 8.76 (d, 2H, J = 7.5 Hz, ArH), 7.30 (m, 2H, ArH), 7.24 (m, 2H, ArH), 7.17 (m, 1H, ArH), 4.39 (m, 2H, CH_2), 3.44 (m, 1H, CH), 1.37 (d, 3H, J = 7.0 Hz, CH_3). ^{13}C NMR (125 MHz, $CDCl_3$, 25 °C): δ 162.28, 158.82, 143.21, 133.15, 131.25, 128.85, 128.45, 127.70, 127.44, 126.84, 126.81, 122.86, 47.41, 38.05, 18.69. Anal. Calcd (%) for ($C_{23}H_{15}NO_5$): C, 71.68; H, 3.92; N, 3.63. Found: C, 71.68; H, 3.91; N, 3.65.

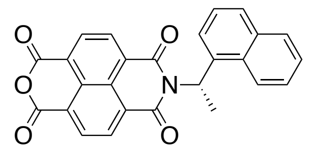

(*S*)-**G5**

(*S*)-**G5.** To a solution of **G1** (300 mg, 1.11 mmol) in DMF (3 mL) was added (*S*)-(–)-1-(1-naphthyl)ethylamine (0.18 mL, 1.1 mmol) at 90 ºC under argon, and the reaction mixture was stirred at 180 ºC for 25 min. After cooling to ambient temperature, the solvent was removed under reduced pressure. The resulting brown residue was then suspended in acetone, and the suspension was slowly poured into 1 M aqueous HCl under vigorous stirring. The precipitate was collected, washed with H_2O and MeOH, and dried under vacuum. A part of the crude product (100 mg) was then purified by SEC ($CHCl_3$ as eluent), affording (*S*)-**G5** (34.2 mg, 33% yield) as a yellow solid. Mp: 310 °C (decomp.). IR (KBr, cm^{-1}): 1785 ($\nu_{C=O}$), 1737 ($\nu_{C=O}$), 1708 ($\nu_{C=O}$), 1672 ($\nu_{C=O}$). ^{1}H NMR (500 MHz, $CDCl_3$, 25 °C): δ 8.75 (s, 4H, ArH), 8.09 (d, 1H, J = 7.5 Hz, ArH), 7.91 (m, 1H, ArH), 7.84 (m, 2H, ArH), 7.61 (dd, 1H, J = 8.0, 7.5 Hz ArH), 7.38 (m, 2H, ArH), 7.07 (q, 1H, J = 7.0 Hz, CH), 2.15 (d, 3H, J = 7.0 Hz, CH_3). ^{13}C NMR (125 MHz, $CDCl_3$, 25 °C): δ 162.40, 158.93, 134.17, 133.84, 133.23, 131.67, 131.58, 129.36, 128.93, 128.86, 128.27, 128.01, 127.02, 126.78, 125.46, 125.09, 122.81, 122.67, 49.19, 17.22. Anal. Calcd (%) for ($C_{26}H_{15}NO_5$): C, 74.11; H, 3.59; N, 3.32. Found: C, 74.08; H, 3.58; N, 3.41.

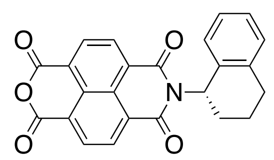

(*S*)-**G6**

(*S*)-**G6.** To a solution of **G1** (300 mg, 1.11 mmol) in DMF (3 mL) was added (*S*)-(+)-1,2,3,4-tetrahydro-1-napthylamine (0.17 mL, 1.1 mmol) at 80 ºC under argon and the reaction mixture was stirred at 170 ºC for 15 min. After cooling to ambient temperature, the solvent was removed under reduced pressure. The resulting brown residue was then suspended in acetone, and the suspension was slowly poured into 1 M aqueous HCl under vigorous stirring. The precipitate was collected and dissolved in $CHCl_3$, and the solution was dried over anhydrous $MgSO_4$, filtered, and concentrated under reduce pressure. The crude product was purified by SEC ($CHCl_3$ as eluent), affording (*S*)-**G6** (274 mg, 40% yield) as a pale yellow solid. Mp: 267.9–272.4 °C. IR (KBr, cm^{-1}): 1786 ($\nu_{C=O}$), 1742 ($\nu_{C=O}$), 1710 ($\nu_{C=O}$), 1671 ($\nu_{C=O}$). ^1H NMR (500 MHz, $CDCl_3$, 25 °C): δ 8.81 (m, 4H, ArH), 7.19 (d, J = 8.0 Hz, 1H, ArH), 7.14 (dd, 1H, J = 7.5, 7.0 Hz, ArH), 7.02 (dd, 1H, J = 8.0, 7.0 Hz, ArH), 6.92 (d, 1H, J = 7.5 Hz, ArH), 6.44 (dd, 1H, J = 15.5, 7.0 Hz, CH), 3.09 (m, 1H, CH_2), 2.86 (m, 1H, CH_2), 2.51 (m, 1H, CH_2), 2.19 (m, 2H, CH_2), 1.92 (m, 1H, CH_2). ^{13}C NMR (125 MHz, $CDCl_3$, 25 °C): δ 162.21, 158.97, 138.04, 135.37, 133.33, 131.60, 129.43, 129.02, 128.28, 127.08, 126.74, 126.34, 124.71, 122.92, 52.56, 29.75, 27.24, 23.15. Anal. Calcd (%) for ($C_{24}H_{15}NO_5$): C, 72.54; H, 3.80; N, 3.52. Found: C, 74.54; H, 3.77; N, 3.54.

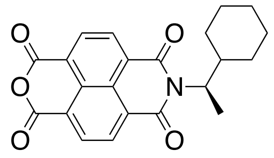

(*R*)-**G7**

(*R*)-**G7.** To a solution of **G1** (300 mg, 1.11 mmol) in DMF (3 mL) was added (*R*)-(–)-1-cyclohexylethylamine (0.12 mL, 1.1 mmol) at 90 ºC under argon and the reaction mixture was stirred at 180 ºC for 15 min. After cooling to ambient temperature, the solvent was removed under reduced pressure. The resulting brown residue was then suspended in acetone, and the suspension was slowly poured into 1 M aqueous HCl under vigorous stirring. The precipitate was collected, washed with *n*-hexane, and dried under vacuum. The crude product was purified by SEC ($CHCl_3$ as eluent), affording (*R*)-**G7** (88.7 mg, 21% yield) as a white solid. Mp: 248.5–249.3 °C. IR (KBr, cm^{-1}): 1788 ($\nu_{C=O}$), 1749 ($\nu_{C=O}$), 1709 ($\nu_{C=O}$), 1667 ($\nu_{C=O}$). ^1H NMR (500 MHz, $CDCl_3$, 25 °C): δ 8.80 (m, 4H, ArH), 4.94 (m, 1H, CH), 2.32 (m, 1H, CH), 2.02 (m, 1H, CH_2), 1.81 (m, 1H, CH_2), 1.57-1.66 (m, 5H, CH_3, CH_2), 1.46 (m, 1H, CH_2), 1.30 (m, 1H, CH_2), 1.16 (m, 2H, CH_2), 1.04 (m, 1H,

CH_2), 0.93 (m, 1H, CH_2). ^{13}C NMR (125 MHz, $CDCl_3$, 25 °C): δ 162.84, 159.05, 133.29, 131.41, 128.94, 128.38, 127.11, 122.72, 55.83, 39.34, 31.14, 30.14, 26.26, 26.03, 25.86, 16.56. Anal. Calcd (%) for ($C_{26}H_{15}NO_5$): C, 70.02; H, 5.07; N, 3.71. Found: C, 70.01; H, 4.95; N, 3.76.

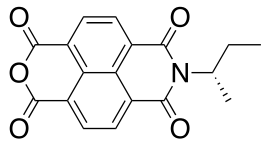

(*S*)-**G8**

(*S*)-**G8.** To a solution of **G1** (349 mg, 1.30 mmol) in DMF (20 mL) was added a solution of (*S*)-(+)-*sec*-butylamine (0.11 mL, 1.1 mmol) in DMF (10 mL) at 140 ºC under argon. The reaction mixture was stirred at 140 ºC for 40 min and further at 100 ºC for 2 h. After cooling to ambient temperature, the solvent was removed under reduced pressure. The resulting brown residue was then suspended in acetone, and the suspension was slowly poured into a MeOH/H_2O (1/1, v/v) mixture under vigorous stirring. The precipitate was collected and dissolved in $CHCl_3$, and the solution was dried over anhydrous Na_2SO_4, filtered, and concentrated under reduce pressure. The crude product was purified by SEC ($CHCl_3$ as eluent), affording (*S*)-**G8** (138 mg, 36% yield) as a white solid. Mp: 217.9–219.8 °C. IR (KBr, cm^{-1}): 1785 ($\nu_{C=O}$), 1742 ($\nu_{C=O}$), 1715 ($\nu_{C=O}$), 1668 ($\nu_{C=O}$). ^1H NMR (500 MHz, $CDCl_3$, 25 °C): δ 8.81 (d, 2H, J = 7.5 Hz, ArH), 8.79 (d, 2H, J = 7.5 Hz, ArH), 5.19 (m, 1H, CH), 2.19 (m, 1H, CH_2), 1.98 (m, 1H, CH_2), 1.59 (d, 3H, J = 7.0 Hz, CH_3), 0.92 (t, 3H, J = 8.0 Hz, CH_3). ^{13}C NMR (125 MHz, $CDCl_3$, 25 °C): δ 162.79, 159.05, 133.30, 131.31, 128.96, 128.47, 127.08, 122.73, 52.47, 26.51, 18.07, 11.59. Anal. Calcd (%) for ($C_{18}H_{13}NO_5$): C, 66.87; H, 4.05; N, 4.33. Found: C, 66.88; H, 3.91; N, 4.33.

Computational Analysis for the Diastereomeric Inclusion Complexes of (*M*)-$\mathbf{1}^{2-}$⊃(*S*)-G2 and (*M*)-$\mathbf{1}^{2-}$⊃(*R*)-G2. We calculated the stabilization energies (E_{stb}) of (*S*)-**G2** and (*R*)-**G2** gained by the inclusion complexation with (*M*)-$\mathbf{1}^{2-}$ in order to obtain further insight into the origin of the diastereoselective encapsulation of racemic guests in (*M*)-$\mathbf{1}^{2-}$. The geometry optimizations were carried out using the self-consistent-charge density-functional tight-binding (SCC-DFTB)[4] method as implemented in the DFTB+ program[5], which can be considered as an approximate density functional theory (DFT)[6,7] with Hamiltonian and overlap matrix elements obtained from DFT using a two-center and minimum valence basis set treatment. We refer to this method as DFTB in the remainder of the text for simplicity. The mio parameter set[4], rscc-materials set[8] and borg set[9] were employed for X and Y (X, Y = C, H, O and N), for B and X (X = C, O and N), and for B and H elements pairs, respectively. The DFTB geometry optimizations were conducted using an electronic temperature of 300 K, and UFF type dispersion correction[10] was included in all calculations. After the DFTB structures were fully optimized, we then re-optimized them again using the RI-TPSS-D3/def-SV(P)[11–13] level of full DFT theory including the D3 dispersion correction[14,15] as implemented in the Turbomole quantum chemistry package[16].

The initial structures of (*M*)-$\mathbf{1}^{2-}$⊃(*S*)-**G2** and (*M*)-$\mathbf{1}^{2-}$⊃(*R*)-**G2** were constructed according to the following procedures (Supplementary Fig. 16): the ^{t}Bu groups of the crystal structure of (*M*)-$\mathbf{1}^{2-}$⊃**G1**[1] were replaced with hydrogen atoms to simplify the calculations. The initial model of (*M*)-$\mathbf{1}^{2-}$⊃**G1** without countercations was then fully optimized by the above-mentioned DFT calculations with the D3 dispersion correction. The **G1** moiety of the resultant energy-minimized structure of (*M*)-$\mathbf{1}^{2-}$⊃**G1** was manually replaced with (*S*)- and (*R*)-**G2**. The relative positions of **G2** and (*M*)-$\mathbf{1}^{2-}$ were determined in order to avoid any unfavorable van der Waals contacts, giving two initial geometries for each of (*S*)- and (*R*)-**G2** in (*M*)-$\mathbf{1}^{2-}$ (**SA** and **SB** for (*M*)-$\mathbf{1}^{2-}$⊃(*S*)-**G2** and **RA** and **RB** for (*M*)-$\mathbf{1}^{2-}$⊃(*R*)-**G2**, respectively), in which the twist angles (ϕ) between the longer axis of the **NMI** moiety of **G2** and the longer axis of one of the two porphyrin rings of (*M*)-$\mathbf{1}^{2-}$ for **SA**, **SB**, **RA** and **RB** were set to be *ca*. –70, –45, –90 and –45°, respectively. The geometry optimizations of (*M*)-$\mathbf{1}^{2-}$⊃(*S*)-**G2** and (*M*)-$\mathbf{1}^{2-}$⊃(*R*)-**G2** without countercations were performed by the above-mentioned DFT calculations with the D3 dispersion correction, affording the corresponding geometry-optimized structures, $\mathbf{SA_{opt}}$, $\mathbf{SB_{opt}}$, $\mathbf{RA_{opt}}$ and $\mathbf{RB_{opt}}$, with the ϕ values of *ca*. –89, –37, –90 and –43, respectively (Supplementary Fig. 16).

The E_{stb} values of the (*S*)-**G2** and (*R*)-**G2** molecules gained by the inclusion complexation with (*M*)-$\mathbf{1}^{2-}$ were evaluated as follows.

$$E_{stb} = E_{complex} - \left(E_{(M)-\mathbf{1}^{2-}} + E_{\mathbf{G2}}\right) \qquad (1)$$

where E_{comlex}, $E_{(M)-\mathbf{1}^{2-}}$ and $E_{\boldsymbol{G2}}$ are the energies of the optimized structures of (*M*)-**1**$^{2-}$⊃**G2**, (*M*)-**1**$^{2-}$ and **G2**, respectively, which were individually obtained by the geometry optimizations with the D3 dispersion correction.

In order to understand the importance of the dispersion interaction, the optimized geometries of the diastereomeric inclusion complexes, (*M*)-**1**$^{2-}$⊃**G2** (**SA$_{opt}$**, **SB$_{opt}$**, **RA$_{opt}$** and **RB$_{opt}$**), obtained above and their extracted (*M*)-**1**$^{2-}$ and **G2** structures were further employed for the single point DFT calculations without any dispersion correction. We also estimated the interaction energies (E_{int}) of the (*S*)-**G2** and (*R*)-**G2** molecules gained by the inclusion complexation with (*M*)-**1**$^{2-}$ without any dispersion correction as follows.

$$E_{int} = E'_{complex} - \left(E'_{(M)-\mathbf{1}^{2-}} + E'_{\boldsymbol{G2}}\right) \qquad (2)$$

where $E'_{complex}$, $E'_{(M)-\mathbf{1}^{2-}}$ and $E'_{\boldsymbol{G2}}$ are the single point calculation energies of the above-mentioned (*M*)-**1**$^{2-}$⊃**G2**, (*M*)-**1**$^{2-}$ and **G2**, respectively.

The optimized geometries of (*M*)-**1**$^{2-}$⊃(*S*)-**G2** (**SA$_{opt}$** and **SB$_{opt}$**) and (*M*)-**1**$^{2-}$⊃(*R*)-**G2** (**RA$_{opt}$** and **RB$_{opt}$**) are shown in Supplementary Fig. 17, and their E_{stb} values are summarized in Supplementary Table 3 and Supplementary Fig. 18a. The E_{stb} value of **SA$_{opt}$** is 1.9–2.8 kcal mol^{-1} lower than those of **SB$_{opt}$**, **RA$_{opt}$** and **RB$_{opt}$**, indicating that (*M*)-**1**$^{2-}$⊃(*S*)-**G2** is more stable than (*M*)-**1**$^{2-}$⊃(*R*)-**G2**, which are in good agreement with the experimental results. The E_{int} values are also summarized in Supplementary Table 4 and Supplementary Fig. 18b. The E_{int} values of both (*M*)-**1**$^{2-}$⊃(*S*)-**G2** and (*M*)-**1**$^{2-}$⊃(*R*)-**G2** were significantly decreased as compared to their corresponding E_{stb} values, suggesting that dispersion interactions such as π–π or CH–π interactions between the host and guest molecules strongly affect the stabilization of the inclusion complexes.

Molecular Modeling of (*M*)-1^{2-}⊃(*S*)-G4 and (*M*)-1^{2-}⊃(*R*)-G4. The initial structures of (*M*)-**1**$^{2-}$⊃(*S*)-**G4** and (*M*)-**1**$^{2-}$⊃(*R*)-**G4** were constructed based on the above-mentioned $\mathbf{SA_{opt}}$ and $\mathbf{RB_{opt}}$ by replacing the chiral pendant group of **G2** with that of **G4**. The initial models were then fully optimized by the DFT calculations using the dispersion corrected B3LYP (B3LYP-D3)[14] functional with the 6-31G* (for H, C, N, and O atoms) and the 6-31+G* (for B atoms) basis sets in *Gaussian 16* software[17]. Computer resources for the DFT calculations were provided by the Information Technology Center of Nagoya University. The resultant energy-minimized structures are depicted in Supplementary Fig. 19.

General Procedure for Exchange Spectroscopy (EXSY) Experiments. The EXSY (2D NOESY) measurements[18] of (*M*)-$\mathbf{1_{TBA2}}$ complexed with (*S*)-**G2** were conducted at –25 °C in THF-d_8 at various mixing times (τ_m) to estimate the exchange rate constant (k_{ex}) between the two rotational conformers resulting from slow rotation of the (*S*)-1-phenylethyl group around the N-C$^{\alpha}$ bond of (*S*)-**G2** sandwiched between the bisporphyrin of (*M*)-$\mathbf{1_{TBA2}}$ at –25 °C (Supplementary Figs 21a and 24). The exchange rate constant (k_{ex}) was determined by the following equations:

$$\mathrm{A} \underset{k_{BA}}{\overset{k_{AB}}{\rightleftarrows}} \mathrm{B}$$

$$k_{ex} = k_{AB} + k_{BA}$$

$$= (1/\tau_m) \ln[(r + 1)/(r - 1)] \qquad (3)$$

where r is defined as the following equation.

$$r = 4X_AX_B(I_{AA} + I_{BB})/(I_{AB} + I_{BA}) - (X_A - X_B)^2 \qquad (4)$$

where X_A and X_B are the mole fractions of A and B, respectively, I_{AB} and I_{BA} are the peak volumes of the cross peaks between the signals A and B, respectively, and I_{AA} and I_{BB} are the peak volumes of the diagonal signals.

The k_{ex} value was estimated from the initial slope of the linear plots of $\ln[(r + 1)/(r - 1)]$ as a function of τ_m (Supplementary Fig. 29).

Supplementary References

1. Yamamoto, S., Iida, H. & Yashima, E. Guest-induced unidirectional dual rotary and twisting motions of a spiroborate-based double-stranded helicate containing a bisporphyrin unit. *Angew. Chem., Int. Ed.* **52**, 6849-6853 (2013).
2. Gottlieb, H. E., Kotlyar, V. & Nudelman, A. NMR chemical shifts of common laboratory solvents as trace impurities. *J. Org. Chem.,* **62**, 7512-7515 (1997).
3. Fulmer, G. R. *et al*. NMR chemical shifts of trace impurities: Common laboratory solvents, organics, and gases in deuterated solvents relevant to the organometallic chemist. *Organometallics* **29**, 2176–2179 (2010).
4. Elstner, M. *et al.* Self-consistent-charge density-functional tight-binding method for simulations of complex materials properties. *Phys. Rev. B* **58,** 7260–7268 (1998).
5. Aradi, B., Hourahine, B. & Frauenheim, T. DFTB+, a sparse matrix-based implementation of the DFTB method. *J. Phys. Chem. A* **111,** 5678–5684 (2007).
6. Hohenberg, P. & Kohn, W. Inhomogeneous Electron Gas. *Phys. Rev.* **136,** B864–B871 (1964).
7. Kohn, W. & Sham, L. J. Self-consistent equations including exchange and correlation effects. *Phys. Rev.* **140,** (1965).
8. Lukose, B., Kuc, A., Frenzel, J. & Heine, T. On the reticular construction concept of covalent organic frameworks. *Beilstein J. Nanotechnol.* **1,** 60–70 (2010).
9. Grundkötter-Stock, B. *et al.* SCC-DFTB Parametrization for Boron and Boranes. *J. Chem. Theory Comput.* **8,** 1153–1163 (2012).
10. Rappe, A. K., Casewit, C. J., Colwell, K. S., Goddard, W. A. & Skiff, W. M. UFF, a full periodic table force field for molecular mechanics and molecular dynamics simulations. *J. Am. Chem. Soc.* **114,** 10024–10035 (1992).
11. Eichkorn, K., Weigend, F., Treutler, O. & Ahlrichs, R. Auxiliary basis sets for main row atoms and transition metals and their use to approximate Coulomb potentials. *Theor. Chem. Accounts Theory, Comput. Model. (Theoretica Chim. Acta)* **97,** 119–124 (1997).
12. Tao, J., Perdew, J. P., Staroverov, V. N. & Scuseria, G. E. Climbing the Density Functional Ladder: Nonempirical Meta–Generalized Gradient Approximation Designed for Molecules and Solids. *Phys. Rev. Lett.* **91,** 146401 (2003).
13. Eichkorn, K., Treutler, O., Öhm, H., Häser, M. & Ahlrichs, R. Auxiliary basis sets to approximate Coulomb potentials (Chem. Phys. Letters 240 (1995) 283-290). *Chem. Phys. Lett.* **242,** 652–660 (1995).

14. Grimme, S., Antony, J., Ehrlich, S. & Krieg, H. A consistent and accurate ab initio parametrization of density functional dispersion correction (DFT-D) for the 94 elements H-Pu. *J. Chem. Phys.* **132,** 154104 (2010).
15. Grimme, S., Ehrlich, S. & Goerigk, L. Effect of the damping function in dispersion corrected density functional theory. *J. Comput. Chem.* **32,** 1456–1465 (2011).
16. Furche, F. *et al.* Turbomole. *Wiley Interdiscip. Rev. Comput. Mol. Sci.* **4,** 91–100 (2014).
17. Gaussian 16, Revision B.01, M. J. Frisch, *et al.*, Gaussian, Inc., Wallingford CT, 2016.
18. Perrin, C. L. & Dwyer, T. J., Application of two-dimensional NMR to kinetics of chemical exchange. *Chem. Rev.* **90**, 935–967 (1990).
